# Substituent-controlled construction of A_4_B_2_-hexaphyrins and A_3_B-porphyrins: a mechanistic evaluation

**DOI:** 10.3762/bjoc.19.135

**Published:** 2023-12-06

**Authors:** Seda Cinar, Dilek Isik Tasgin, Canan Unaleroglu

**Affiliations:** 1 Department of Chemistry, Hacettepe University, Beytepe Campus 06800, Ankara, Turkey; 2 Ankara Hacı Bayram Veli University, Chemistry Department, Polatli Campus, 06900 Polatli, Ankara, Turkey; 3 Inter-Curricular Courses Department, Çankaya University, Central Campus, 06790 Etimesgut, Ankara, Turkey

**Keywords:** A_4_B_2_-hexaphyrin, A_3_B-porphyrin, *N*-tosylimine, Cu(OTf)_2_ catalysis, HRESI–TOF analysis

## Abstract

A substituent-dependent construction of novel A_3_B-porphyrins along with A_4_B_2_-hexaphyrins was realized by the reactions of *N*-tosylimines and *meso*-aryl-substituted tripyrranes in the presence of Cu(OTf)_2_ as the catalyst. The reaction mechanism of the presented method was studied on model reactions by electrospray-ionization time-of-flight (HRESI–TOF) mass spectral analysis in a timely manner. The analytical results indicated that the observed azafulvene-ended di- and tripyrrolic intermediates are responsible for the formation of porphyrinogen and hexaphyrinogen forms.

## Introduction

Porphyrins and expanded porphyrins have found widespread applications in supramolecular chemistry [1–4]. Expanded porphyrins are utilized as building blocks in the fields of near-infrared (NIR) dyes [5], nonlinear optical (NLO) materials [2], and photosensitizers in photodynamic therapy [1], however, their synthesis is still a challenge for chemists. Hexaphyrins are one of the most investigated structures among expanded porphyrins owing to their structural stability, flexibility, or complexing ability with transition metals [6–12]. *meso*-Aryl-substituted dipyrromethanes or tripyrranes are the most commonly used starting materials in hexaphyrin syntheses [13–16]. Osuka et al. made significant contributions to the selective synthesis of expanded porphyrins and their chemistry with regard to their aromaticity and coordination properties. They used *meso*-aryl-substituted dipyrromethanes and aldehydes in the synthesis of A_3_B_3_-type hexaphyrins [13] and 5,10-diaryl-substituted tripyrranes in A_4_B_2_-hexaphyrin synthesis [6,17–20]. Similarly, the syntheses of A*_m_*B*_n_*-type hexaphyrins, octaphyrins, or higher expanded porphyrins were handled by improved methods in recent years with the use of tripyrranes or bilanes and aldehydes [7,14,21–25].

**Scheme 1:**
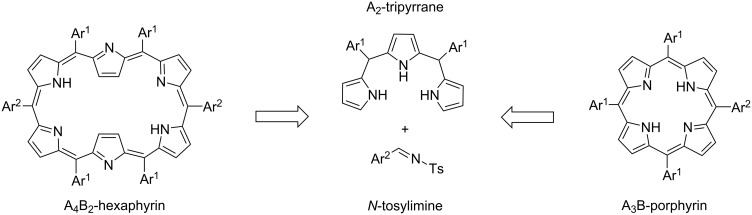
Retrosynthetic method for A_4_B_2_-hexaphyrin and A_3_B-porphyrin synthesis.

In our previous studies, we used *N*-tosylimines throughout the syntheses of several porphyrinic compounds which emphasized the usability of *N*-tosylimines with dipyrromethanes, tripyrranes, or bilanes instead of aldehydes in the synthesis of porphyrins and contracted/expanded porphyrins [26–28]. It was shown that the reaction of *meso*-pentafluorophenyl-substituted *N*-tosylimine and 5,10-bis(pentafluorophenyl)tripyrromethane formed A_6_-hexaphyrin as the main product along with the inevitable formation of side products, A_4_-porphyrin and higher expanded porphyrins [28]. *meso*-Phenyloligopyrroles having electron-rich substituents at the 2-, 4-, or 6-positions were screened in the literature. To the best of our knowledge, hexaphyrin synthesis from the least substituted aryls appears to be not much studied. In the following study, we focused on the use of less hindered variety of precursors in hexaphyrin and porphyrin synthesis via the Cu(OTf)_2_-catalyzed reaction of tripyrrane and tosylimine according to the retrosynthetic method given in Scheme 1. Here, we present the substituent-dependent selective construction of A_4_B_2_-hexaphyrins and A_3_B-porphyrins with good yields without the formation of expanded counterparts. Beyond the synthesis, for better understanding of the product formation, mass spectral analyses of model reactions were investigated by time-dependent electrospray-ionization time-of-flight (HRESI–TOF) technique.

## Results and Discussion

Reactions of *meso*-pentafluorophenyl-substituted A_2_-tripyrrane **1** and *N*-tosylimines **2** were performed in the presence of Cu(OTf)_2_ (Table 1). Initially, the unsubstituted phenyl-bearing *N*-tosylimine **2a** was reacted with tripyrrane **1** in CH_2_Cl_2_ under previously reported conditions [28], however, the desired hexaphyrin could not been isolated. Under these conditions, only product **3a**, which is defined as A_3_B-type porphyrin was isolated with 15% yield (Table 1, entry 1). Then, we run the reactions of A_2_-tripyrrane **1** with mesityl-containing tosylimine **2b** and 2,6-dichlorobenzylidene-substituted substrate **2c**. The desired A_4_B_2_-hexaphyrins **4b** and **4c** were obtained in 17% and 16% yield, respectively (Table 1, entries 2 and 3). Next, to elucidate the role of substituents present in the aromatic part of the *N*-tosylimines, the monohalogenated *N*-tosylimines **2d**–**f** and *N*-tosylimine **2g** with a strongly electron-withdrawing CF_3_ substituent in the 4-position were subjected to the reaction with tripyrrane **1**. These *para*-substituted *N*-tosylimines provided the A_4_B_2_-hexaphyrins **4d**–**g** (Table 1, entries 4–7), with the A_4_B_2_-hexaphyrin **4d** isolated with 18% yield. The products **4e**–**g** were obtained in 7–10% yield and their formation was corroborated by HRMS spectral analysis (Figures S64–S66 in Supporting Information File 1). In these reactions, the A_3_B-porphyrins concomitantly formed in yields between 9–17%. When *p*-methoxy- and *p*-hydroxy-substituted *N*-tosylimines **2h** and **2i** were used in this reaction, substrate **2h** gave only the A_3_B-porphyrin while the imine **2i** did not form any product (Table 1, entries 8 and 9). To further evaluate the scope of the reaction, heteroaryl-bearing tosylimines were also tested. The thiophene-substituted tosylimine **2j** gave hexaphyrin **4j** in 17% yield and porphyrin **3j** in 10% yield, whereas the indole-bearing tosylimine gave only A_3_B-porphyrins but no A_4_B_2_-hexaphyrin (Table 1, entries 10 and 11). Signals of trace amounts of A_2_B_2_-type porphyrins were detected in the mass spectra of some of the products. ^1^H NMR analysis of the synthesized hexaphyrins proved that the spectra were in consistence with [26]hexaphyrin aromaticity [29]. Several other metal triflates such as Zn(OTf)_2_, Gd(OTf)_3_, and Yb(OTf)_3_ were also tested as catalysts in the reaction of 4-fluorophenyl-substituted tosylimine **2d** and tripyrrane **1** and lower yields of the A_4_B_2_-hexaphyrins and A_3_B-porphyrins were obtained compared to the reaction catalyzed by Cu(OTf)_2_ (Table S2 in Supporting Information File 1).

**Table 1:** Synthesis of A_3_B-porphyrins and A_4_B_2_-hexaphyrins.

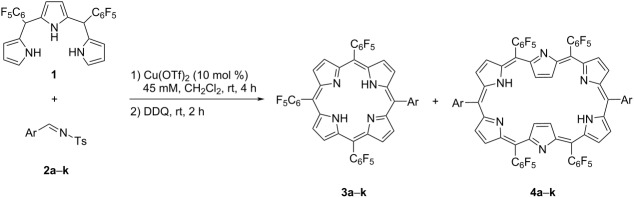				

Entry	*N*-Tosylimine **2**	Ar	Yield^a^ (%) **3a**–**h**,**j**,**k**	Yield^a^ (%) **4b**–**g**,**j**

1	**a**	C_6_H_5_	15	–
2	**b**	2,4,6-(CH_3_)_3_C_6_H_2_	17	17
3	**c**	2,6-Cl_2_-C_6_H_3_	13	16
4	**d**	4-FC_6_H_4_	17	18
5	**e**	4-ClC_6_H_4_	9	7^b^
6	**f**	4-BrC_6_H_4_	16	10^b^
7	**g**	4-CF_3_C_6_H_4_	13	10^b^
8	**h**	4-(OCH_3_)C_6_H_4_	22	–
9	**i**	4-(OH)C_6_H_4_	–	–
10	**j**	thiophen-2-yl	10	17
11	**k**	indol-3-yl	22	–

^a^Isolated yields after flash column chromatography; ^b^identified by HRMS analysis, NMR spectra could not be recorded due to low solubility.

The synthesis of A_3_B-porphyrins is effortful and only few studies have been reported involving the use of A_3_-bilanes [30–31] or dipyrromethane–dicarbinols [32], the modification of A_4_-porphyrins [33], or the reaction of pyrrole with different aldehydes [34]. In the present work, the applied synthetic method provided the A_3_B-porphyrins in a single-step reaction from bispentafluorophenyl-substituted tripyrrane **1** and variously substituted *N*-tosylimines **2** along with the targeted [26]hexaphyrins. Additionally, each reaction was also run with aldehydes to compare the effectiveness of *N*-tosylimines and aldehydes on this system. In most cases, the yields were lower than those in the reactions with *N*-tosylimines for both A_4_B_2_-hexaphyrins and A_3_B-porphyrins (Table S1 in Supporting Information File 1).

Until now, we have investigated the effect of substituents present in the aryl substituent of the *N*-tosylimines on the product formation. At this point, we chose 5,10-bis(4-trifluoromethylphenyl)tripyrromethane (**5**) as a representative example to investigate the role of the tripyrrane on the reaction. A series of reactions of tripyrrane **5** with tosylimines **2c**,**d**,**f**,**h**,**l**,**m** were performed. In this case, tripyrrane **5** principally formed A_3_B-porphyrins (Table 2, entries 1–6) and in some cases A_2_B_2_-porphyrins, but disfavored the formation of A_4_B_2_-hexaphyrins. As outlined in Table 2, the reactions of *N*-tosylimines **2d**,**f**,**l**,**m** with tripyrrane **5** resulted in the formation of A_3_B-porphyrins **6b**,**c**,**e**,**f** in yields ranging between 12–28%, respectively, where the corresponding A_2_B_2_-porphyrins were formed in trace amounts (Table 2, entries 2, 3, 5, and 6). A_3_B-porphyrin **6a** was isolated as the sole product with 13% yield (Table 2, entry 1). In the case of *N*-tosylimine **2h**, the reaction gave A_3_B-porphyrin **6d** and *trans*-A_2_B_2_-porphyrin **7d** with 21% and 10% yield, respectively (Table 2, entry 4).

**Table 2:** Synthesis of A*_m_*B*_n_*-type porphyrins with electron-deficient tripyrrane **5**.

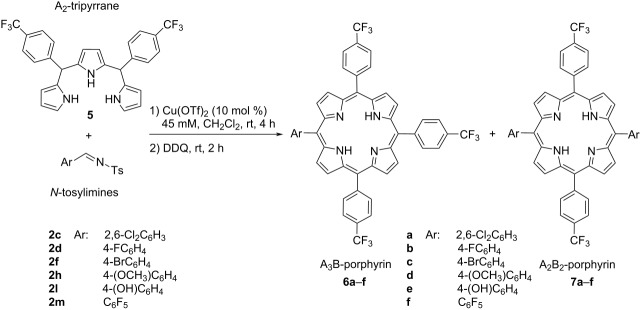					

Entry	*N*-Tosylimine	A_3_B-porphyrin	Yield^a^ (%)	A_2_B_2_-porphyrin	Yield^a^ (%)

1	**2c**	**6a**	13	**7a**	–
2	**2d**	**6b**	28	**7b**	trace^b^
3	**2f**	**6c**	15	**7c**	trace^b^
4	**2h**	**6d**	21	**7d**	10
5	**2l**	**6e**	12	**7e**	trace^b^
6	**2m**	**6f**	12	**7f**	trace^b^

^a^Isolated yields after flash column chromatography; ^b^identified by HRMS analysis.

In this work, the role of substituents on tripyrranes and *N*-tosylimines on product formation has been shown and the synthesis of A_3_B-porphyrins and a variety of A_4_B_2_-hexaphyrins has been achieved. The presence of the bulky electron-withdrawing pentafluorophenyl group in tripyrranes controls the formation of A_4_B_2_-hexaphyrins as mentioned by Osuka and Suzuki [13], besides the formation of A_3_B-porphyrins. On the other hand, electron-withdrawing but less bulky (4-trifluoromethylphenyl) groups on tripyrrane **5** led to predominant formation of the A_3_B-porphyrin even when it was reacted with mono-, di-, or penta-substituted aryl *N*-tosylimino substrates (Table 2).

To elucidate the product diversity and to follow the progress of the reaction, a series of mass spectral analysis of the reaction mixture of 4-fluorophenyl-substituted *N*-tosylimine **2d** and tripyrrane **1** has been conducted at 0 °C. Samples were taken from the reaction medium at certain time intervals within 2 hours and examined by ESI LC–MS.

**Figure 1:**
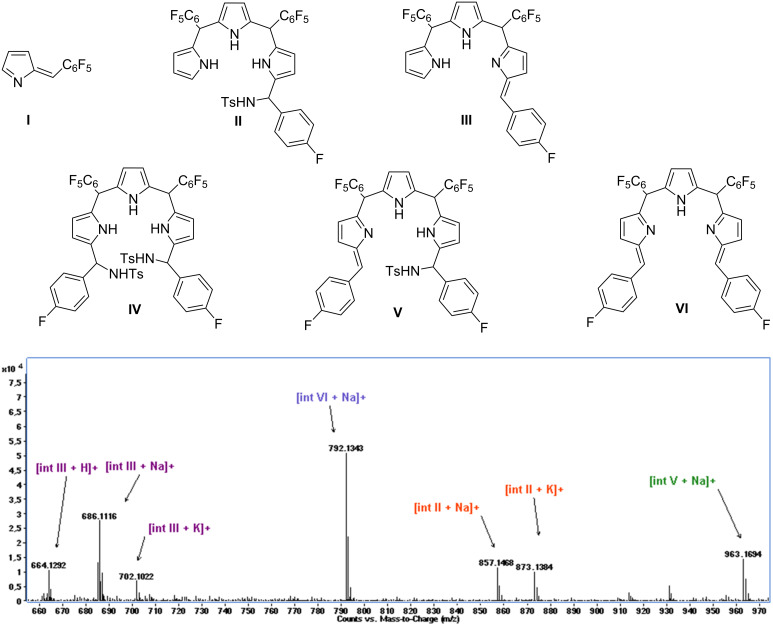
Mass spectrum of the reaction mixture of **1** and **2d** at 30 min at 0 °C with assigned intermediates (positive ion mode).

Throughout the high-resolution electrospray-ionization time-of-flight (HRESI–TOF) mass analysis of the reaction mixture at 0 °C, the following peaks were observed: *m/z* = 246.0366 ([M + H]^+^ calcd for C_11_H_5_F_5_N, 246.0337), *m*/*z* = 857.1468 ([M + Na]^+^ calcd for C_40_H_25_F_11_N_4_O_2_SNa, 857.1415), *m*/*z* = 664.1292 ([M + H]^+^ calcd for C_33_H_17_F_11_N_3_, 664.1241), *m*/*z* = 1134.2119 ([M + Na]^+^ calcd for C_54_H_37_F_12_N_5_O_4_S_2_Na, 1134.1988), *m*/*z* = 963.1694 ([M + Na]^+^ calcd for C_47_H_28_F_12_N_4_O_2_SNa, 963.1634), *m*/*z* = 792.1343 ([M + Na]^+^ calcd for C_40_H_19_F_12_N_3_Na, 792.1280), corresponding to the intermediates **I**–**VI**, respectively (Figure 1, Figures S46 and S47 in Supporting Information File 1). At the very first two minutes of the reaction run at 0 °C no mass signals attributable to reaction intermediates were observed but only signals of the starting materials **1** and **2d** both in the negative and positive ion mode. After two minutes, tripyrrane sulfonamide **II** and azafulvene **I** mass peaks were observed. Later on, tripyrrolic intermediates **III** and **VI** predominated and the mass peak of **IV** was observed with poor intensity in the spectra (Figure 1 and Figure S47 in Supporting Information File 1).

In our previous works, we have shown that the reaction of pyrrole with *N*-tosylimines leads to pyrrole sulfonamides as the main products [35]. In another work, in the synthesis of dipyrromethane structures, we have proven the formation of azafulvene intermediates by Cu(OTf)_2_-appended elimination of sulfonamide groups from pyrrolic sulfonamides [36]. Here in this work, during the reaction at 0 °C, intermediates **I**–**VI** were detected (Figure 1). The primary intermediates **II** and **IV** are formed by the addition of tripyrrane **1** to tosylimine **2d**. Further elimination of *N*-tosyl group(s) from these intermediates gives azafulvene-ended secondary intermediates **III**, **V**, and **VI**. The observed intermediates **I**–**VI** having sulfonamide or azafulvene ends are in accordance with our previous findings [26,35–36]. In addition, the observation of azafulvene **I** could be attributed to the fragmentation of tripyrrane **1**, intermediates **II** or **III** as proposed in Figure S75 in Supporting Information File 1. These structures (**I**–**VI**) could be said to be responsible for the selective formation of porphyrin and hexaphyrin products. When the temperature was increased to rt, porphyrinogen forms of A_3_B-porphyrin and A_4_B_2_-hexapyhrin were predominately observed (Figure S48 in Supporting Information File 1).

**Scheme 2:**
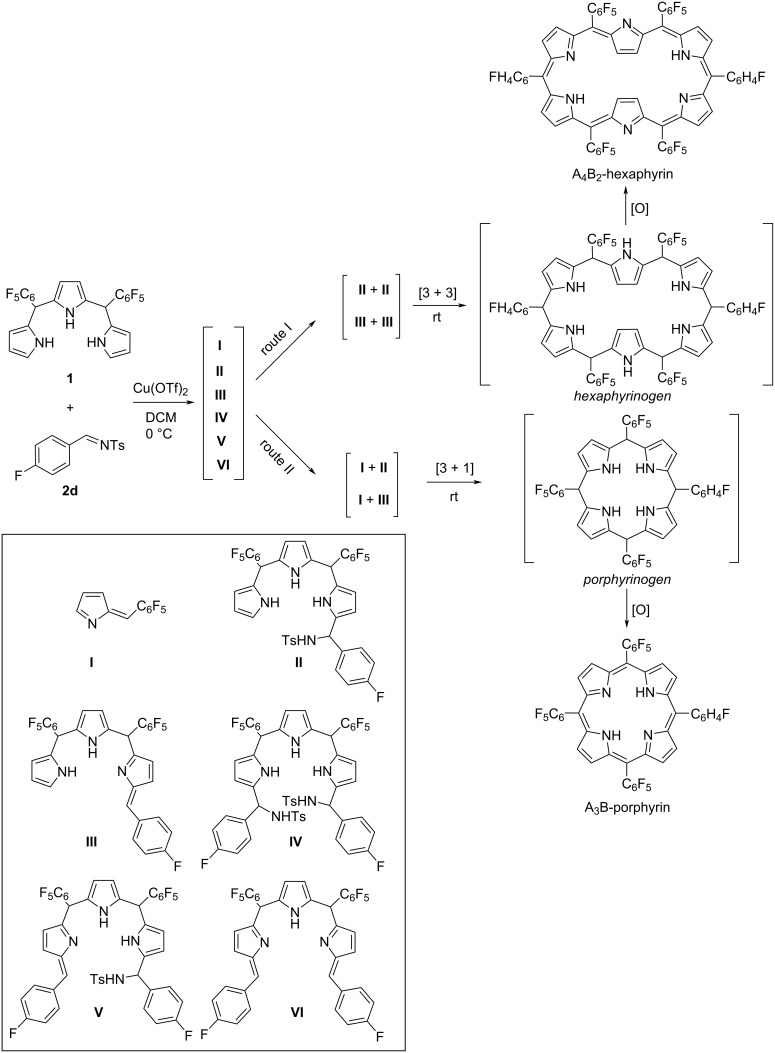
A suggested reaction pathway for the formation of A_4_B_2_-hexaphyrins and A_3_B-porphyrins.

According to high-resolution electrospray-ionization time-of-flight (HRESI–TOF) analysis, at the beginning of the reaction, mass peaks of intermediates **II** and **IV** arose as a result of tripyrrane **1** addition to *N*-tosylimine **2d**. Further eliminations of sulfonamide groups from **II** and **IV** formed the intermediates **III**, **V** and **VI**. A plausible reaction pathway for the formation of A_4_B_2_-hexaphyrin and A_3_B-porphyrin was suggested taking into account the combination of these detected intermediates (Scheme 2). In route I, [3 + 3] reactions of intermediates **II** or **III**, at rt form hexaphyrinogen and the subsequent oxidation gives A_4_B_2_-hexaphyrin. Similarly, [3 + 1] reactions of intermediates **I** and **II** or **I** and **III** provide the formation of porphyrinogen and their oxidation gives A_3_B-porphyrin as indicated in route II. The presence of starting material **1** in the reaction medium can provide the formation of A_4_B_2_-hexaphyrin through the reaction with the intermediates **IV**, **V**, or **VI**. These routes are considered as less likely to happen where intermediates **II** and **III** dominate the reaction medium according to the mass analysis as stated before. For this reason, in Scheme 2 below, more probable cyclization pathways have been displayed.

**Figure 2:**
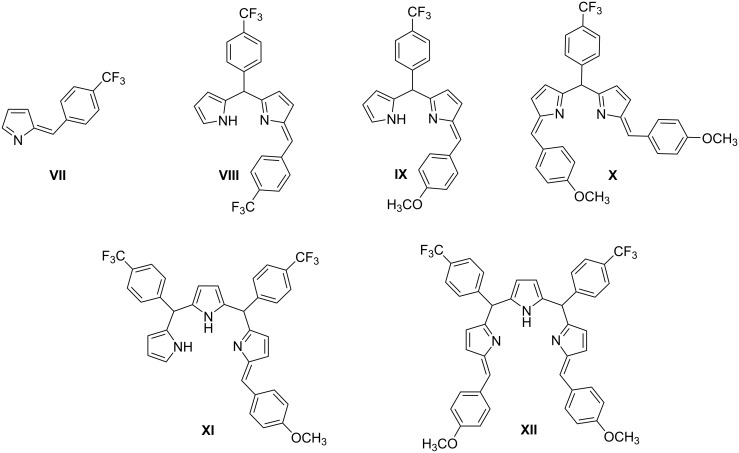
Intermediates in the reaction mixture of **5** and **2h** at 30 min at 0 °C.

A similar LC–MS analysis was made for the reaction of tripyrrane **5** and 4-methoxyphenyl-substituted tosylimine **2h** at 0 °C which mainly formed A_3_B-porphyrins. This time, the primary tosylated intermediates were not detected, instead *N*-tosyl eliminated azafulvene-ended secondary intermediates **VII**–**XII** (Figure 2) were observed, respectively, at *m*/*z* = 224.0627 ([M + H]^+^ calcd for C_12_H_9_F_3_N, 224.0682), *m*/*z* = 445.1150 ([M − H]^−^ calcd for C_24_H_15_F_6_N_2_, 445.1145), *m*/*z* = 409.1411 ([M + H]^+^ calcd for C_24_H_20_F_3_N_2_O, 409.1522), *m*/*z* = 527.1795 ([M + H]^+^ calcd for C_32_H_26_F_3_N_2_O_2_, 527.1941), *m*/*z* = 630.1985 ([M − H]^−^ calcd for C_36_H_26_F_6_N_3_O, 630.1986), and *m*/*z* = 750.2364 ([M + H]^+^ calcd for C_44_H_34_F_6_N_3_O_2_, 750.2550) (Figure S49 in Supporting Information File 1). Although the positive ion peaks of tripyrrolic intermediates **XI** and **XII** were observed, any hexaphyrin products did form from this set of reactions. Yet, A_3_B-porphyrins were clearly and selectively formed over A_2_B_2_-porphyrins, even the positive ion peaks of dipyrrolic intermediates **VIII**, **IX**, and **X** have been observed (Figure S49 in Supporting Information File 1). A reaction pathway for the predominant formation of A_3_B-porphyrin considering the reaction of tripyrrane **5** and tosylimine **2h** was also proposed and is given in Figure S50 of Supporting Information File 1, in which only the azafulvene-ended intermediates **VII**–**XII** were detected.

## Conclusion

In conclusion, a set of A_4_B_2_-hexaphyrins and A_3_B-porphyrins were selectively synthesized through the Cu(OTf)_2_-catalyzed reactions of *N*-tosylimines and tripyrranes under mild reaction conditions. With these reactions, it has been shown that the C_6_F_5_ group led to the formation of hexaphyrin and porphyrins as well as the monosubstituted aryl-bearing *N*-tosylimines, but a 4-(CF_3_)C_6_H_4_ group led only to the formation of porphyrin compounds.

A mechanistic perspective for the formation of porphyrinic products was acquired via a set of high-resolution mass analyses of selected model reactions. The results indicated that azafulvene-ended tripyrrolic intermediates **III**, **V**, and **VI** or sulfonamide-ended intermediates **II** and **IV** along with monopyrrolic fragment **I** derives the formation of porphyrins and hexaphyrins. This study offers an insight to the design of A_4_B_2_-hexaphyrins and A_3_B-porphyrins by utilizing the substituents on tripyrranes and *N*-tosylimines.

## Experimental

**General method:** All reagents and solvents were purchased from Sigma-Aldrich, Fisher Scientific, or Acros Organics and were used without further purification. ^1^H NMR (400 MHz), ^13^C NMR (100 MHz), and ^19^F NMR (376 MHz) spectra were recorded on a Bruker 400, Ultra Shield high-performance digital FT-NMR spectrometer. Data for ^1^H NMR, ^13^C NMR, and ^19^F NMR are reported as follows: chemical shift (δ, ppm), multiplicity (s = singlet, d = doublet, t = triplet, m = multiplet, q= quartet, bs = broad singlet, dd = doublet of doublets, td = triplet of doublets, qd = quartet of doublets), coupling constant, number of atoms. UV–vis absorption spectra were recorded on a Mapada Instruments UV3100PC spectrophotometer. Mass spectra were recorded on an Agilent 1200/6210 high-resolution mass time-of-flight (TOF) LC–MS spectrometer. Reactions were followed by thin-layer chromatography (TLC, Kieselgel 60, F254, Merck) with visualization under UV light. Products were purified by silica gel flash column chromatography (0.05–0.63 mm, 230–400 mesh ASTM, E.Merck). *N*-Tosylimines **2a**–**m** and 5,10-bis(pentafluorophenyl)tripyrromethane (**1**) were synthesized according to the previously reported literature procedures [35,37].

### Synthesis of porphyrin compounds **3a–h**,**j**,**k** and **4b–g**,**j**

*N*-Tosylimine **2** (0.090 mmol) and Cu(OTf)_2_ (0.0090 mmol) were dissolved in CH_2_Cl_2_ (0.5 mL) and stirred at room temperature for 30 minutes under N_2_ atmosphere. To this mixture was added a solution of 5,10-bis(pentafluorophenyl)tripyrromethane (**1**, 0.090 mmol) in CH_2_Cl_2_ (1.5 mL) and the mixture was stirred at rt for 4 h. Afterwards, DDQ (0.180 mmol) was added to this solution and stirred for another 2 h. The resulting solution was eluted through a short silica gel column with EtOAc and the solvent was removed under reduced pressure. The residue was purified by silica gel column chromatography (EtOAc/hexane 1:50) or preparative thin-layer chromatography on silica gel (Silica gel 60, F254, Merck) where applicable to obtain A_3_B-porphyrins and A_4_B_2_-hexaphyrins. Yields of porphyrins **3a**–**k** were between 9–22% and the yields of hexaphyrins **4b**–**g**,**j** were between 7–18%.

### Synthesis of tripyrrane **5**

5,10-Bis(4-trifluoromethylphenyl)tripyrromethane (**5**) was obtained as side product of dipyrromethane synthesis by the condensation of pyrrole and 4-(trifluoromethyl)benzaldehyde. A typical procedure involves 4-(trifluoromethyl)benzaldehyde (28.7 mmol) and pyrrole (143.6 mmol) in 3 mL:197 mL HCl/H_2_O. The resulting mixture was controlled by TLC and after 4 h, the mixture was extracted with EtOAc (50 mL × 3). The reaction crude was then purified by flash column chromatography (EtOAc/hexane 1:10) to give compound **5** in 20% yield.

### Synthesis of porphyrin compounds **6a–f** and **7d**

*N*-Tosylimine **2** (0.097 mmol) and Cu(OTf)_2_ (0.0097 mmol) were dissolved in CH_2_Cl_2_ (0.5 mL) and stirred at room temperature for 30 min under N_2_ atmosphere. To this mixture was then added a solution of 5,10-bis(4-trifluoromethylphenyl)tripyrromethane (**5**, 0.097 mmol) in CH_2_Cl_2_ (1.5 mL) and stirred at rt for 4 h. Afterwards, DDQ (0.195 mmol) was added to this solution and stirred for another 2 h. The resulting solution was eluted through a short silica gel column with EtOAc and the solvent was removed under reduced pressure. The residue was purified by silica gel column chromatography (EtOAc/hexane 1:50) or preparative thin-layer chromatography on silica gel (silica gel 60, F254, Merck) where applicable to obtain A_3_B-porphyrins **6a–f**, and **7d** in yields between 10–28%.

## Supporting Information

File 1 Analytical data and copies of spectra.
