# Supplementary material for: Substituent-controlled construction of A4B2-hexaphyrins and A3B-porphyrins: a mechanistic evaluation
Source: Beilstein J Org Chem. 2023 Dec 6;19:1832–40. doi: 10.3762/bjoc.19.135 (PMC10714476; doi:10.3762/bjoc.19.135)
Supplement: File 1 — Analytical data and copies of spectra. [file Beilstein_J_Org_Chem-19-1832-s001.pdf]

BEILSTEIN JOURNAL OF ORGANIC CHEMISTRY

Supporting Information

for

Substituent-controlled construction of A_4B_2-hexaphyrins and A_3B-porphyrins: a mechanistic evaluation

Seda Cinar, Dilek Isik Tasgin and Canan Unaleroglu

Beilstein J. Org. Chem. **2023,** *19,* 1832–1840. doi:10.3762/bjoc.19.135

Analytical data and copies of spectra

License and Terms: This is a supporting information file under the terms of the Creative Commons Attribution License (https://creativecommons.org/licenses/by/4.0). Please note that the reuse, redistribution and reproduction in particular requires that the author(s) and source are credited and that individual graphics may be subject to special legal provisions.
The license is subject to the *Beilstein Journal of Organic Chemistry* terms and conditions: (https://www.beilstein-journals.org/bjoc/terms)

Table of contents

1. Experimental section S1

2. Table S1. Reaction of tripyrrane **1** and aldehydes S6

3. Table S2. Catalyst effect on the product formation of reaction of **1** and **2d** S7

4. NMR spectra S8

5. LC MS-TOF (ESI) spectra of reaction intermediates S31

6. LC MS-TOF (ESI) spectra of compounds S33

1. Experimental section

*5,10,15-Tris(pentafluorophenyl)-20-phenylporphyrin (**3a**).* Purple solid (8 mg, 15%); UV–vis (CH_2Cl_2): λ_{max}[nm] (logε)= 413.0 (5.393), 508.5 (4.207), 537.0 (3.629), 583.0 (3.741), 638.5 (3.092); ^{1}H NMR (400 MHz, $CDCl_3$) δ: -2.82 (s, 2H), 7.80 – 7.88 (m, 3H), 8.24 (d, *J*= 6.6 Hz, 2H), 8.85 (d, *J*= 3.9 Hz, 2H), 8.92 (s, 4H), 9.00 (d, *J*= 4.2 Hz, 2H); ^{19}F NMR (376 MHz, $CDCl_3$) δ: -136.55 (dd, J_1= 23.9 Hz, J_2= 8.4 Hz, 2F), -136.62 (dd, J_1= 23.4 Hz, J_2= 8.4 Hz, 4F), -151.72 (m, 3F), -161.59 (m, 6F); HRMS (ESI-TOF) m/z calcd. for $C_{44}H_{16}F_{15}N_4$ $[M+H]^+$: 885.1130, found: 885.1151.

*5,10,15-Tris(pentafluorophenyl)-20-(2,4,6-trimethylphenyl)porphyrin (**3b**).* Purple solid (10 mg, 17%), UV–vis ($CHCl_3$): λ_{max}[nm] (logε)= 415(5.678), 510(4.528), 586(4.138); ^{1}H NMR (400 MHz, $CDCl_3$) δ: -2.80 (s, 2H), 1.83 (s, 6H), 2.65 (s, 3H), 7.31 (s, 2H), 8.77 (s, 2H), 8.82 (s, 2H), 8.89 (s, 4H); ^{19}F NMR (376 MHz, $CDCl_3$) δ: -136.52 (td, J_1= 24.7 Hz, J_2= 8.0 Hz, 6F), -151.72 (m, 3F), -161.58 (qd, J_1= 23.8 Hz, J_2= 8.0 Hz, 6F); HRMS (ESI-TOF) m/z calcd. for $C_{47}H_{22}F_{15}N_4$ $[M+H]^+$: 927.1605, found: 927.1610.

*5,10,15-Tris(pentafluorophenyl)-20-(2,6-dichlorophenyl)porphyrin (**3c**).* Purple solid (8 mg, 13%), UV–vis ($CHCl_3$): λ_{max}[nm] (logε)= 414(5.311), 509(4.067), 590(4.021); ^{1}H NMR (400 MHz, $CDCl_3$) δ: -2.82 (s, 2H), 7.74 – 7.79 (m, 1H), 7.83-7.85 (m, 2H), 8.81 – 8.84 (m, 4H), 8.89 (s, 4H); ^{19}F NMR (376 MHz, $CDCl_3$) δ: -136.34 (dd, J_1= 23.8 Hz, J_2= 8.5 Hz, 4F), -136.51 (dd, J_1= 23.8 Hz, J_2= 8.0 Hz, 2F), -151.53 (m, 3F), -161.51 (m, 6F); HRMS (ESI-TOF) m/z calcd. for $C_{44}H_{14}Cl_2F_{15}N_4$ $[M+H]^+$: 953.0350, found: 953.0364.

*5,10,15-Tris(pentafluorophenyl)-20-(4-fluorophenyl)porphyrin (**3d**).* Purple solid (10 mg, 17%), UV–vis (CH_2Cl_2): λ_{max}[nm] (logε)= 413.5 (5.798), 509.0 (4.648), 540.0 (3.952), 584.0 (4.164), 639.0 (3.499); ^{1}H NMR (400 MHz, $CDCl_3$) δ: -2.96 (s, 2H), 7.40 – 7.45 (m, 2H), 8.11

(bs, 2H), 8.76 (s, 2H), 8.82 (s, 4H), 8.87 (s, 2H); ^{19}F NMR (376 MHz, $CDCl_3$) δ: -113.58 (s, 1F), -136.51 (dd, J_1= 23.7 Hz, J_2= 8.0 Hz, 2F), -136.65 (dd, J_1= 23.4 Hz, J_2= 8.3 Hz, 4F), -151.63 (m, 3F), -161.56 (m, 6F); HRMS (ESI-TOF) m/z calcd. for $C_{44}H_{15}F_{16}N_4$ $[M+H]^+$: 903.1036, found: 903.1013.

*5,10,15-Tris(pentafluorophenyl)-20-(4-chlorophenyl)porphyrin (**3e**).* Purple solid (6 mg, 9%), UV–vis (CH_2Cl_2): λ_{max}[nm] (logε)= 414.0 (5.607), 509.0 (4.405), 539.5 (3.778), 584.5 (3.936), 639.0 (3.304); ^{1}H NMR (400 MHz, $CDCl_3$) δ: -2.89 (s, 2H), 7.79 (d, *J*= 8.1 Hz, 2H), 8.15 (d, *J*= 8.1 Hz, 2H), 8.83 (d, *J*= 4.8 Hz, 2H), 8.90 (s, 4H), 8.95 (d, *J*= 4.8 Hz, 2H); ^{19}F NMR (376 MHz, $CDCl_3$) δ: -136.51 (dd, J_1= 24.4 Hz, J_2= 9.3 Hz, 2F), -136.65 (dd, J_1= 24.2 Hz, J_2= 8.7 Hz, 4F), -151.64 (m, 3F), -161.58 (m, 6F); HRMS (ESI-TOF) m/z calcd. for $C_{44}H_{15}ClF_{15}N_4$ $[M+H]^+$: 919.0740, found: 919.0720.

*5,10,15-Tris(pentafluorophenyl)-20-(4-bromophenyl)porphyrin (**3f**).* Purple solid (10 mg, 16%), UV–vis (CH_2Cl_2): λ_{max}[nm] (logε)= 413.5 (5.670), 508.5 (4.471), 540.0 (3.825), 583.0 (4.009), 639.0 (3.400); ^{1}H NMR (400 MHz, $CDCl_3$) δ: -2.88 (s, 2H), 7.94 (d, *J*= 8.3 Hz, 2H), 8.09 (d, *J*= 8.1 Hz, 2H), 8.83 (s, 2H), 8.89 (s, 4H), 8.94 (s, 2H); ^{19}F NMR (376 MHz, $CDCl_3$) δ: -136.51 (dd, J_1= 23.6 Hz, J_2= 8.7 Hz, 2F), -136.65 (dd, J_1= 23.8 Hz, J_2= 8.1 Hz, 4F), -151.64 (m, 3F), -161.56 (m, 6F); HRMS (ESI-TOF) m/z calcd. for $C_{44}H_{15}BrF_{15}N_4$ $[M+H]^+$: 963.0235, found: 963.0239.

*5,10,15-Tris(pentafluorophenyl)-20-(4-trifluoromethylphenyl)porphyrin (**3g**).* Purple solid (9 mg, 13%); UV–vis (CH_2Cl_2): λ_{max}[nm] (logε)= 413.0 (5.631), 508.0 (4.465), 537.0 (3.907), 582.5 (4.063), 638.0 (3.639); ^{1}H NMR (400 MHz, $CDCl_3$) δ: -2.88 (s, 2H), 8.08 (d, *J*= 7.9 Hz, 2H), 8.35 (d, *J*= 7.5 Hz, 2H), 8.84 (s, 2H), 8.90 (s, 6H); ^{19}F NMR (376 MHz, $CDCl_3$) δ: -62.10 (s, 3F), -136.50 (dd, J_1= 23.5 Hz, J_2= 7.9 Hz, 2F), -136.64 (dd, J_1= 23.6 Hz, J_2= 7.9 Hz, 4F), -151.44 (m, 3F), -161.43 (m, 6F); HRMS (ESI-TOF) m/z calcd. for $C_{45}H_{15}F_{18}N_4$ $[M+H]^+$: 953.1004, found: 953.0982.

*5,10,15-Tris(pentafluorophenyl)-20-(4-methoxyphenyl)porphyrin (**3h**).* Purple solid (14 mg, 22%); UV–vis (CH_2Cl_2): λ_{max}[nm] (logε)= 416.0 (5.032), 510.5 (3.813), 543.0 (3.121), 586.0 (3.335), 641.0 (2.653); ^{1}H NMR (400 MHz, $CDCl_3$) δ: -2.83 (s, 2H), 4.11 (s, 3H), 7.32 (d, *J*= 8.4 Hz, 2H), 8.12 (d, *J*= 8.4 Hz, 2H), 8.81 (d, *J*= 4.0 Hz, 2H), 8.88 (s, 4H), 9.01 (d, *J*= 4.3 Hz, 2H); ^{19}F NMR (376 MHz, $CDCl_3$) δ: -136.49 (dd, J_1= 23.7 Hz, J_2= 8.3 Hz, 2F), -136.62 (dd, J_1= 23.6 Hz, J_2= 8.2 Hz, 4F), -151.78 (m, 3F), -161.62 (m, 6F); HRMS (ESI-TOF) m/z calcd. for $C_{45}H_{18}F_{15}N_4O$ $[M+H]^+$: 915.1241, found: 915.1244.

*5,10,15-Tris(pentafluorophenyl)-20-(2-thienyl)porphyrin (**3j**).* Purple solid (6 mg, 10%), UV–vis (CH_2Cl_2): λ_{max}[nm] (logε)= 415.0 (5.495), 510.5 (4.276), 585.0 (3.802); ^{1}H NMR (400 MHz, $CDCl_3$) δ: -2.85 (s, 2H), 7.53 – 7.55 (m, 1H), 7.92 (d, *J*= 5.2 Hz, 1H), 7.96 (d, *J*= 3.2 Hz, 1H), 8.83 (d, *J*= 4.3 Hz, 2H), 8.88 (s, 4H), 9.19 (d, *J*= 4.4 Hz, 2H); ^{19}F NMR (376 MHz, $CDCl_3$) δ: -136.48 (dd, J_1= 23.4 Hz, J_2= 8.1 Hz, 2F), -136.60 (dd, J_1= 23.6 Hz, J_2= 8.1 Hz, 4F), -151.62 (m, 3F), -161.52 (m, 6F); HRMS (ESI-TOF) m/z calcd. for $C_{42}H_{14}F_{15}N_4S$ $[M+H]^+$: 891.0694, found: 891.0674.

*5,10,15-Tris(pentafluorophenyl)-20-(3-indolyl)porphyrin (**3k**).* Purple solid (14 mg, 22%); UV–vis (CH_2Cl_2): λ_{max}[nm] (logε)= 415.0 (5.279), 514.5 (4.387), 542.0 (4.152), 588.0 (4.074), 644.0 (3.821); ^{1}H NMR (400 MHz, $CDCl_3$) δ: -2.68 (s, 2H), 7.19 (t, *J*= 7.6 Hz, 1H), 7.44 (t, *J*= 7.7 Hz, 1H), 7.53 (d, *J*= 8.0 Hz, 1H), 7.76 (d, *J*= 8.3 Hz, 1H), 8.02 (s, 1H), 8.75 (d, *J*= 4.1 Hz, 2H), 8.87 (s, 4H), 8.91 (s, 1H), 9.16 (d, *J*= 4.5 Hz, 2H); ^{19}F NMR (376 MHz, $CDCl_3$) δ: -136.44 (m, 6F), -151.93 (m, 3F), -161.65 (m, 6F); HRMS (ESI-TOF) m/z calcd. for $C_{46}H_{17}F_{15}N_5$ $[M+H]^+$: 924.1239, found: 924.1248.

*5,20-Bis(2,4,6-trimethylphenyl)-10,15,25,30-tetrakis(pentafluorophenyl)[26]hexaphyrin (**4b**).* Purple solid (11 mg, 17%), UV–vis ($CHCl_3$): λ_{max}[nm] (logε)= 572.5(4.779), 722(4.066), 901(3.878), 1035(3.825); ^{1}H NMR (400 MHz, $CDCl_3$) δ: -2.77 (s, 4H), 2.02 (s, 12H), 2.71 (s, 6H), 7.42 (s, 4H), 9.08 (d, *J*= 4.7 Hz, 4H), 9.38 (d, *J*= 4.6 Hz, 4H); ^{19}F NMR (376 MHz, $CDCl_3$) δ: -136.91 (m, 8F), -153.59 (m, 4F), -163.44 (m, 8F); HRMS (ESI-TOF) m/z calcd. for $C_{72}H_{37}F_{20}N_6$ $[M+H]^+$: 1365.2755, found: 1365.2748. Inner NH signal was not observed.

*5,20-Bis(2,6-dichlorophenyl)-10,15,25,30-tetrakis(pentafluorophenyl)[26]hexaphyrin (**4c**).* Purple solid (10 mg, 16%), UV–vis ($CHCl_3$): λ_{max}[nm] (logε)= 572.5(4.919), 716.5(4.052), 896.5(3.662), 1025.5(3.562); ^{1}H NMR (400 MHz, $CDCl_3$) δ: -2.62 (s, 4H), -2.37 (bs, 2H), 7.86-7.89 (m, 2H), 7.93-7.96 (m, 4H), 9.04 (d, *J*= 4.8 Hz, 4H), 9.41 (d, *J*= 4.8 Hz, 4H); ^{19}F NMR (376 MHz, $CDCl_3$) δ: -136.74 (d, *J*= 21.1 Hz, 8F), -153.34 (t, *J*= 20.0 Hz, 4F), -163.27 (t, *J*= 19.5 Hz, 8F); HRMS (ESI-TOF) m/z calcd. for $C_{66}H_{21}Cl_4F_{20}N_6$ $[M+H]^+$: 1417.0257, found: 1417.0244.

*5,20-Bis(4-fluorophenyl)-10,15,25,30-tetrakis(pentafluorophenyl)[26]hexaphyrin (**4d**).* Purple solid (10 mg, 18%), UV–vis ($CHCl_3$): λ_{max}[nm] (logε)= 572.5(4.675), 721(3.780), 782.5(3.631), 900(3.487), 1023(3.302); ^{1}H NMR (400 MHz, $CDCl_3$) δ: -2.67 (s, 4H), -2.24 (bs, 2H), 7.54 – 7.58 (m, 4H), 8.27 – 8.30 (m, 4H), 9.10 (d, *J*= 4.2 Hz, 4H), 9.33 (d, *J*= 4.1 Hz, 4H); ^{19}F NMR (376 MHz, $CDCl_3$) δ: -112.57 (s, 2F), -137.03 (d, *J*= 20.9 Hz, 8F), -153.25 (m, 4F), -

163.24 (m, 8F); HRMS (ESI-TOF) m/z calcd. for $C_{66}H_{23}F_{22}N_6$ $[M+H]^+$: 1317.1627, found: 1317.1638.

*5,20-Bis(4-chlorophenyl)-10,15,25,30-tetrakis(pentafluorophenyl)[26]hexaphyrin (**4e**)*. Purple solid (4 mg, 7%), UV–vis ($CHCl_3$): λ_{max}[nm] (logε)= 573.5(4.672), 714.5(4.041), 896.5(3.794); HRMS (ESI-TOF) m/z calcd. for $C_{66}H_{23}Cl_2F_{20}N_6$ $[M+H]^+$: 1349.1036, found: 1349.1015.

*5,20-Bis(4-bromophenyl)-10,15,25,30-tetrakis(pentafluorophenyl)[26]hexaphyrin (**4f**)*. Purple solid (6 mg, 10%), UV–vis ($CHCl_3$): λ_{max}[nm] (logε)= 573.5(4.627), 716.5(3.926), 901(3.548); HRMS (ESI-TOF) m/z calcd. for $C_{66}H_{23}Br_2F_{20}N_6$ $[M+H]^+$: 1437.0026, found: 1436.9979.

*5,20-Bis(4-trifluoromethylphenyl)-10,15,25,30-tetrakis(pentafluorophenyl)[26] hexaphyrin (**4g**)*. Purple solid (7 mg, 10%), UV–vis ($CHCl_3$): λ_{max}[nm] (logε)= 571.5(4.301), 718(3.337), 903(3.113); HRMS (ESI-TOF) m/z calcd. for $C_{68}H_{23}F_{26}N_6$ $[M+H]^+$: 1417.1564, found: 1417.1558.

*5,20-Bis(2-thienyl)-10,15,25,30-tetrakis(pentafluorophenyl)[26]hexaphyrin (**4j**)*. Purple solid (10 mg, 17%), UV–vis ($CHCl_3$): λ_{max}[nm] (logε)= 616.5(4.889), 898(3.939), 1050(3.810); ^{1}H NMR (400 MHz, $CDCl_3$) δ: 4.55 (d, J= 3.7 Hz, 2H), 4.92 (t, J= 4.6 Hz, 2H), 5.35 (d, J= 4.8 Hz, 2H), 7.39 (s, 4H), 7.73 (s, 4H), 8.16 (d, J= 4.5 Hz, 4H); ^{19}F NMR (376 MHz, $CDCl_3$) δ: -136.95 (dd, J_1= 23.7 Hz, J_2= 7.3 Hz, 4F), -137.53 (d, J= 24.1 Hz, 4F), -151.59 (t, J= 20.7 Hz, 4F), -160.68 (td, J_1= 22.3 Hz, J_2= 7.8 Hz, 4F), -160.88 (td, J_1= 22.5 Hz, J_2= 7.8 Hz, 4F); HRMS (ESI-TOF) m/z calcd. for $C_{62}H_{21}F_{20}N_6S_2$ $[M+H]^+$: 1293.0944, found: 1293.0939. Data is convenient with the literature [1] findings.

*5,10-Bis(4-trifluoromethylphenyl)tripyrromethane (**5**)*. Black solid (800 mg, 20%); R_f = 0.13 (1:6, EtOAc:hexane); ^{1}H NMR (400 MHz, $CDCl_3$) δ ppm: 5.43 (s, 2H), 5.74 (d, J= 2.6 Hz, 2H), 5.85 (s, 2H), 6.13 – 6.16 (m, 2H), 6.71 (s, 2H), 7.29 (d, J= 8.2 Hz, 4H), 7.56 (d, J= 8.2 Hz, 4H), 7.81 (bs, 1H), 7.96 (bs, 2H); ^{13}C NMR (100 MHz, $CDCl_3$) δ ppm: 43.9, 107.6, 107.9, 108.6, 117.7, 124.1 (q, J= 273 Hz), 125.6 (q, J= 3.7 Hz), 128.7, 129.4 (q, J= 30.9 Hz), 131.3, 131.8, 146.0; ^{19}F NMR (376 MHz, $CDCl_3$) δ ppm: -62.18 (s, 6F); HRMS (ESI): *m/z* for $C_{28}H_{20}F_6N_3$ $[M-H]^-$ calcd: 512.1567; found: 512.1547.

*5,10,15-Tris(4-trifluoromethylphenyl)-20-(2,6-dichlorophenyl)porphyrin (**6a**)*. Purple solid (9 mg, 13%), UV–vis ($CHCl_3$): λ_{max}[nm] (logε)= 418(5.503), 513(4.099), 543(3.686); ^{1}H NMR (400 MHz, $CDCl_3$) δ: -2.76 (s, 2H), 7.73 – 7.77 (m, 1H), 7.82 – 7.84 (m, 2H), 8.04 (d, J= 7.8 Hz, 6H), 8.32 – 8.37 (m, 6H), 8.72 (d, J= 4.6 Hz, 2H), 8.79 (s, 4H), 8.82 (d, J= 4.4 Hz, 2H);

^{19}F NMR (376 MHz, $CDCl_3$) δ: -62.01 (s, 9F); HRMS (ESI-TOF) m/z calcd. for $C_{47}H_{26}Cl_2F_9N_4$ $[M+H]^+$: 887.1385, found: 887.1354.

5,10,15-Tris(4-trifluoromethylphenyl)-20-(4-fluorophenyl)porphyrin ***(6b)***. Purple solid (17.0 mg, 28%), UV–vis (CH_2Cl_2): λ_{max}[nm] (logε)= 416.0 (5.809), 512.5 (4.577), 547.0 (4.210), 588.0 (4.102), 644.0 (3.821); ^1H NMR (400 MHz, $CDCl_3$) δ: -2.82 (s, 2H), 7.48 – 7.52 (m, 2H), 8.07 (d, *J*= 7.9 Hz, 6H), 8.17 – 8.20 (m, 2H), 8.37 (d, *J*= 7.6 Hz, 6H), 8.83 (s, 6H), 8.88 (s, 2H); ^{19}F NMR (376 MHz, $CDCl_3$) δ: -62.01 (s, 9F), -114.27 (d, *J*= 47.1 Hz, 1F); HRMS (ESI-TOF) m/z calcd. for $C_{47}H_{27}F_{10}N_4$ $[M+H]^+$: 837.2071, found: 837.2070.

5,10,15-Tris(4-trifluoromethylphenyl)-20-(4-bromophenyl)porphyrin ***(6c)***. Purple solid (10.0 mg, 15%), UV–vis ($CHCl_3$): λ_{max}[nm] (logε)= 418.5 (5.375), 513.0 (4.076), 546.0 (3.602), 592.0 (3.329), 645.0 (3.176); ^1H NMR (400 MHz, $CDCl_3$) δ: -2.92 (s, 2H), 7.84 (d, *J*= 8.0 Hz, 2H), 7.97 – 8.02 (m, 8H), 8.35 (d, *J*= 7.7 Hz, 6H), 8.73 (s, 6H), 8.79 (s, 2H); ^{19}F NMR (376 MHz, $CDCl_3$) δ: -62.02 (s, 9F); HRMS (ESI-TOF) m/z calcd. for $C_{47}H_{27}BrF_9N_4$ $[M+H]^+$: 897.1270, found: 897.1270.

5,10,15-Tris(4-trifluoromethylphenyl)-20-(4-methoxyphenyl)porphyrin ***(6d)***. Purple solid (13 mg, 21%); UV–vis (CH_2Cl_2): λ_{max}[nm] (logε)= 418.0 (5.395), 514.5 (4.039), 549.0 (3.560), 589.0 (3.664), 645.0 (3.197); ^1H NMR (400 MHz, $CDCl_3$) δ: -2.90 (s, 2H), 4.03 (s, 3H), 7.23 (d, *J*= 8.4 Hz, 2H), 7.96 (d, *J*= 7.7 Hz, 6H), 8.05 (d, *J*= 8.4 Hz, 2H), 8.26 (d, *J*= 7.7 Hz, 6H), 8.72 (s, 6H), 8.85 (s, 2H); ^{19}F NMR (376 MHz, $CDCl_3$) δ: -62.01 (s, 9F); HRMS (ESI-TOF) m/z calcd. for $C_{48}H_{30}F_9N_4O$ $[M+H]^+$: 849.2270, found: 849.2238.

5,10,15-Tris(4-trifluoromethylphenyl)-20-(4-hydroxyphenyl)porphyrin ***(6e)***. Purple solid (7.5 mg, 12%); UV–vis (CH_2Cl_2): λ_{max}[nm] (logε)= 418.0 (5.744), 514.0 (4.428), 549.0 (4.067), 589.0 (3.949), 645.0 (3.768); ^1H NMR (400 MHz, $CDCl_3$) δ: -2.82 (s, 2H), 8.03 – 8.09 (m, 10H), 8.34 (d, *J*= 7.8 Hz, 6H), 8.80 (s, 6H), 8.93 (d, *J*= 4.5 Hz, 2H); ^{19}F NMR (376 MHz, $CDCl_3$) δ: -62.00 (s, 9F); HRMS (ESI-TOF) m/z calcd. for $C_{47}H_{28}F_9N_4O$ $[M+H]^+$: 835.2119, found: 835.2098.

5,10,15-Tris(4-trifluoromethylphenyl)-20-(pentafluorophenyl)porphyrin ***(6f)***. Purple solid (8 mg, 12%); UV–vis (CH_2Cl_2): λ_{max}[nm] (logε)= 414.5 (5.450), 510.5 (4.204), 543.0 (3.715), 587.0 (3.742), 642.0 (3.314); ^1H NMR (400 MHz, $CDCl_3$) δ: -2.84 (s, 2H), 8.07 (d, *J*= 7.6 Hz, 6H), 8.35 (d, *J*= 7.3 Hz, 6H), 8.81 (s, 6H), 8.89 (s, 2H); ^{19}F NMR (376 MHz, $CDCl_3$) δ: -62.05 (d, *J*= 8.1 Hz, 9F), -136.85 (m, 2F), -151.74 (m, 1F), -161.64 (m, 2F); HRMS (ESI-TOF) m/z calcd. for $C_{47}H_{23}F_{14}N_4$ $[M+H]^+$: 909.1694, found: 909.1698.

*5,15-Bis(4-trifluoromethylphenyl)-10,20-bis(4-methoxyphenyl)porphyrin (**7d**).* Purple solid (6 mg, 10%); UV–vis (CH_2Cl_2): λ_{max}[nm] (logε)= 419.5 (5.431), 516.0 (4.091), 552.0 (3.814), 592.0 (3.623), 647.0 (3.504); ^{1}H NMR (400 MHz, $CDCl_3$) δ: -2.77 (s, 2H), 4.13 (s, 6H), 7.33 (d, *J*= 8.2 Hz, 4H), 8.06 (d, *J*= 7.8 Hz, 4H), 8.15 (d, *J*= 8.2 Hz, 4H), 8.37 (d, *J*= 7.6 Hz, 4H), 8.81 (s, 4H), 8.93 (s, 4H); ^{19}F NMR (376 MHz, $CDCl_3$) δ: -61.99 (s, 6F); HRMS (ESI-TOF) m/z calcd. for $C_{48}H_{33}F_6N_4O_2$ $[M+H]^+$: 811.2502, found: 811.2486.

2. Table S1. Reaction of tripyrrane 1 and aldehydes

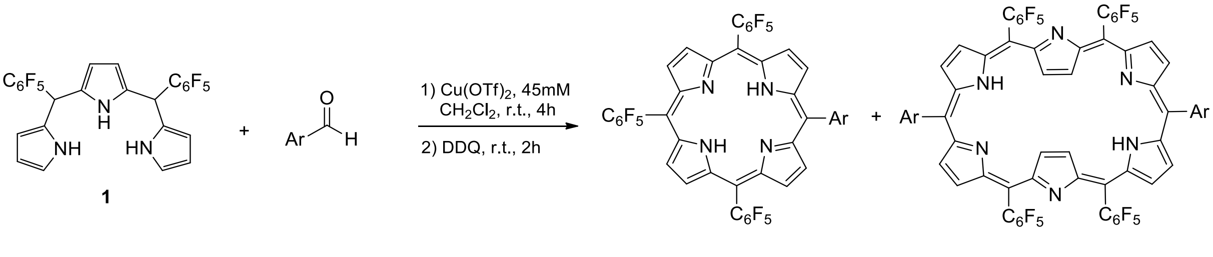

Entry	Aldehyde	Ar	Yield[a] (%) **3a–h,j,k**	Yield[a] (%) **4a–g,j**
1	**a**	C_6H_5	5	7
2	**b**	$2,4,6(CH_3)_3C_6H_2$	10	11
3	**c**	$2,6\text{-}Cl_2\text{-}C_6H_3$	9	9
4	**d**	$4\text{-}FC_6H_4$	8	20
5	**e**	$4\text{-}ClC_6H_4$	10	12
6	**f**	$4\text{-}BrC_6H_4$	11	12
7	**g**	$4\text{-}CF_3C_6H_4$	9	10
8	**h**	$4\text{-}(OCH_3)C_6H_4$	11	-
9	**i**	$4\text{-}(OH)C_6H_4$	-	-
10	**j**	2-Thiophenyl	13	10
11	**k**	3-Indolyl	13	-

[a] isolated yields after flash column chromatography

3. Table S2. Catalyst effect on the product formation of reaction of 1 and 2d

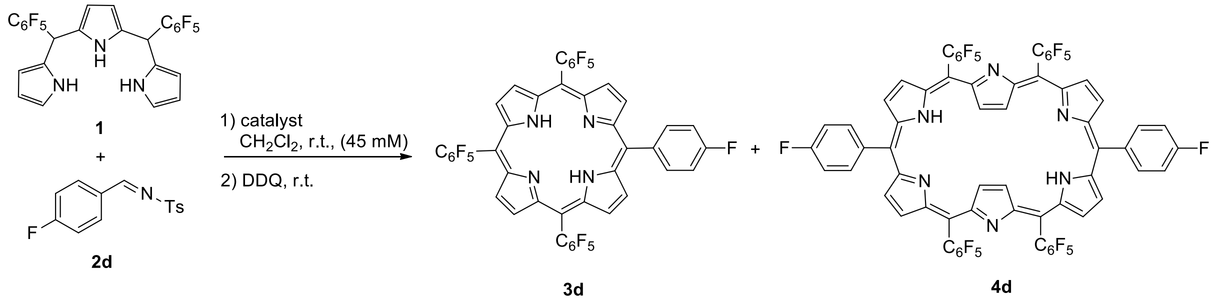

Entry	Catalyst	% Yield 3d	% Yield 4d
1	$Zn(OTf)_2$	8	10
2	$Gd(OTf)_3$	trace	trace
3	$Yb(OTf)_3$	10	13

4. NMR spectra

Peaks between 0.5–2 ppm in ^1H NMR spectra are due to solvent residue [2].

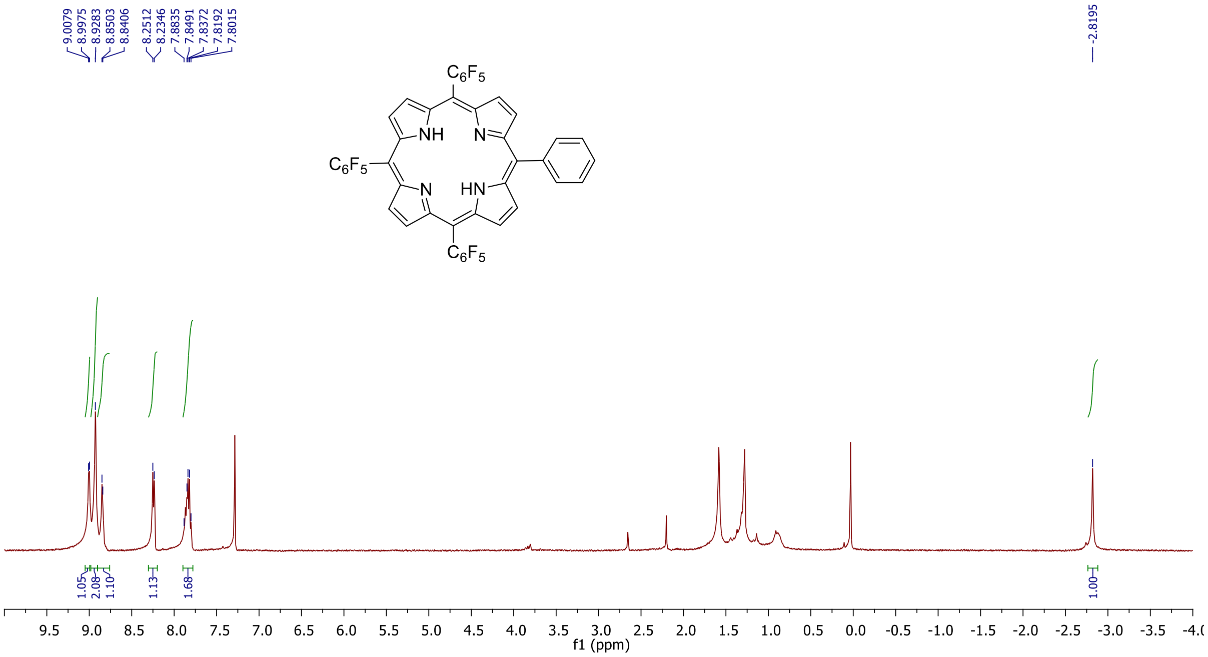

Figure S1. ^1H NMR of 3a ($CDCl_3$)

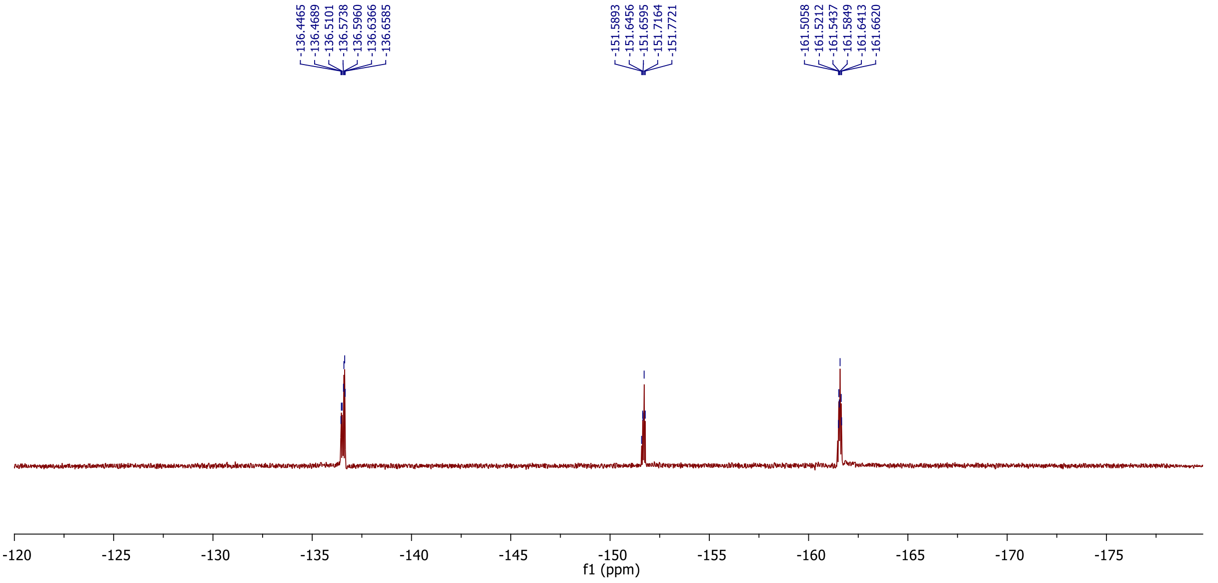

Figure S2. ^{19}F NMR of 3a ($CDCl_3$)

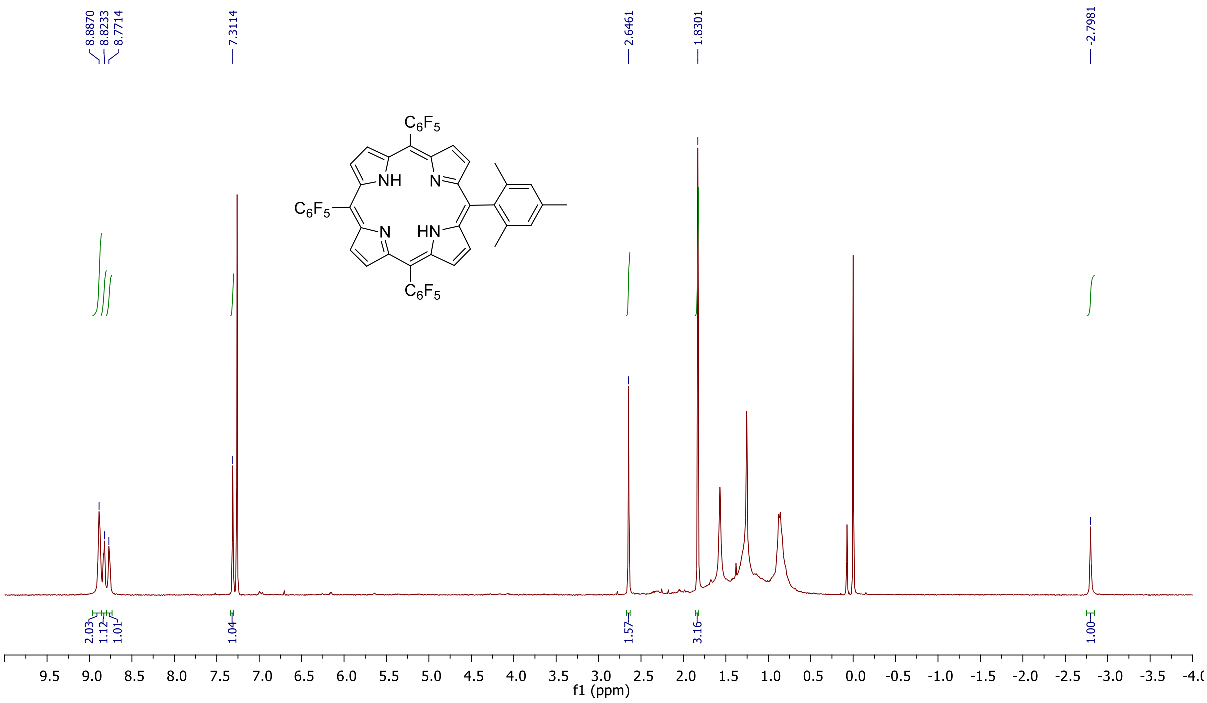

Figure S3. ^{1}H **NMR of 3b ($CDCl_3$)**

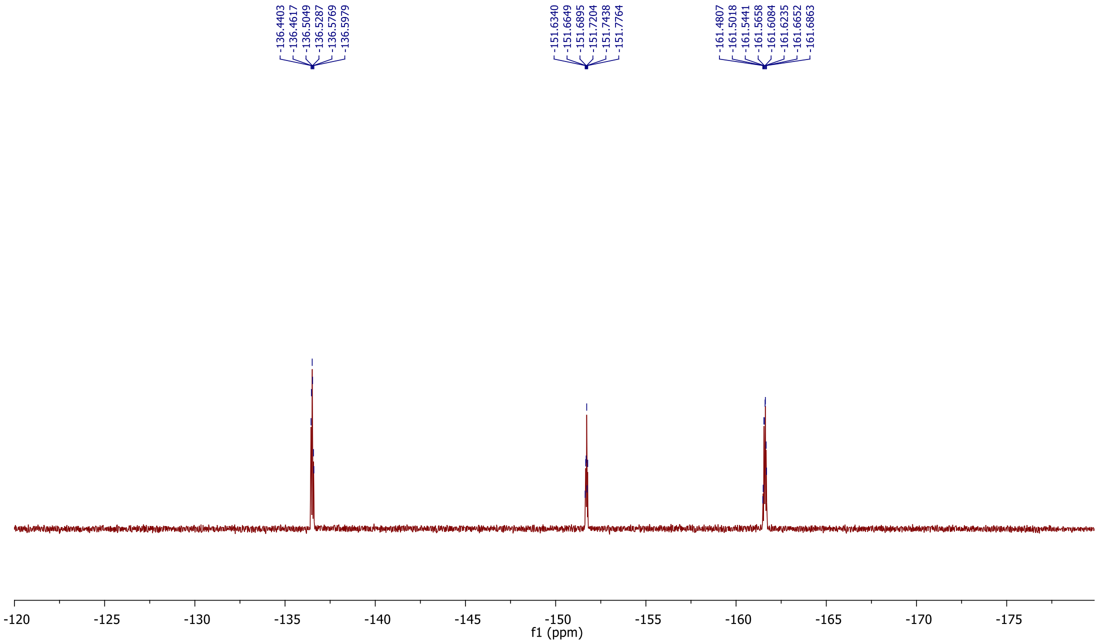

Figure S4. ^{19}F **NMR of 3b ($CDCl_3$)**

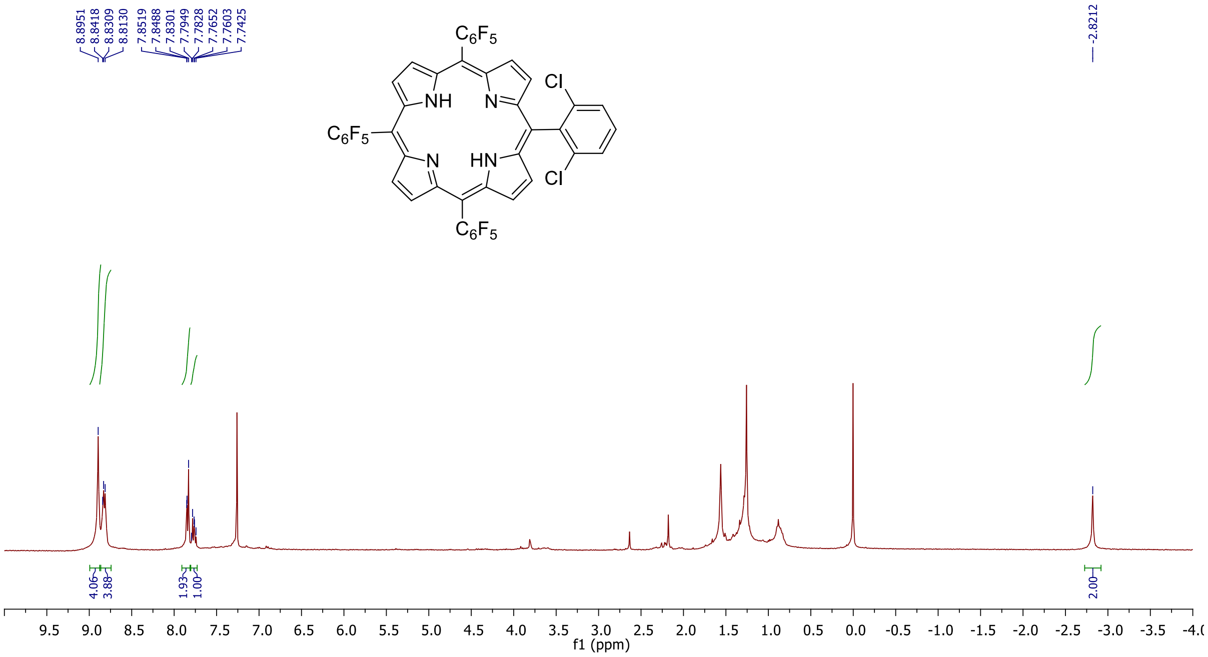

Figure S5. ^{1}H NMR of 3c ($CDCl_3$)

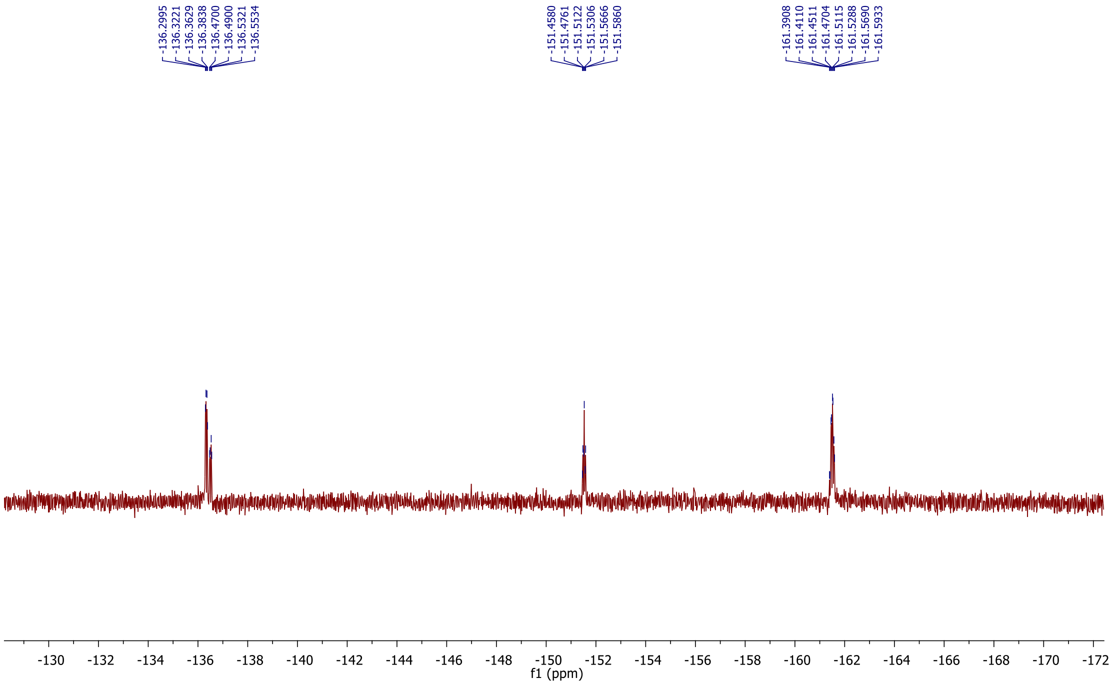

Figure S6. ^{19}F NMR of 3c ($CDCl_3$)

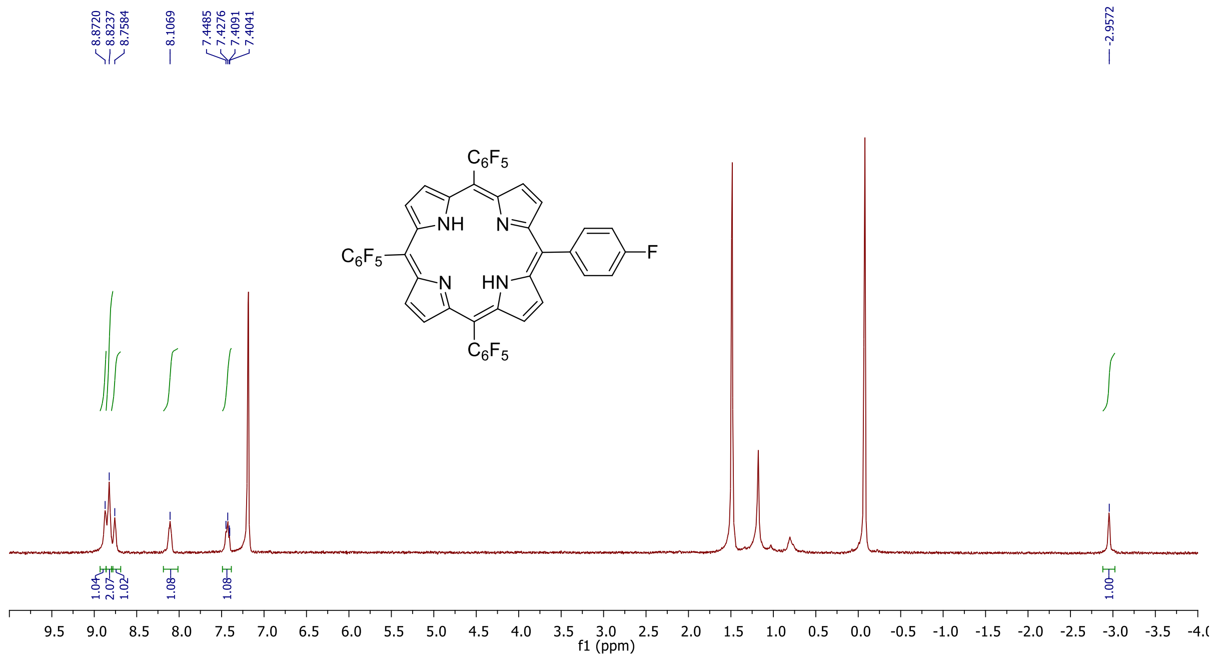

Figure S7. ^{1}**H NMR of 3d (CDCl$_3$)**

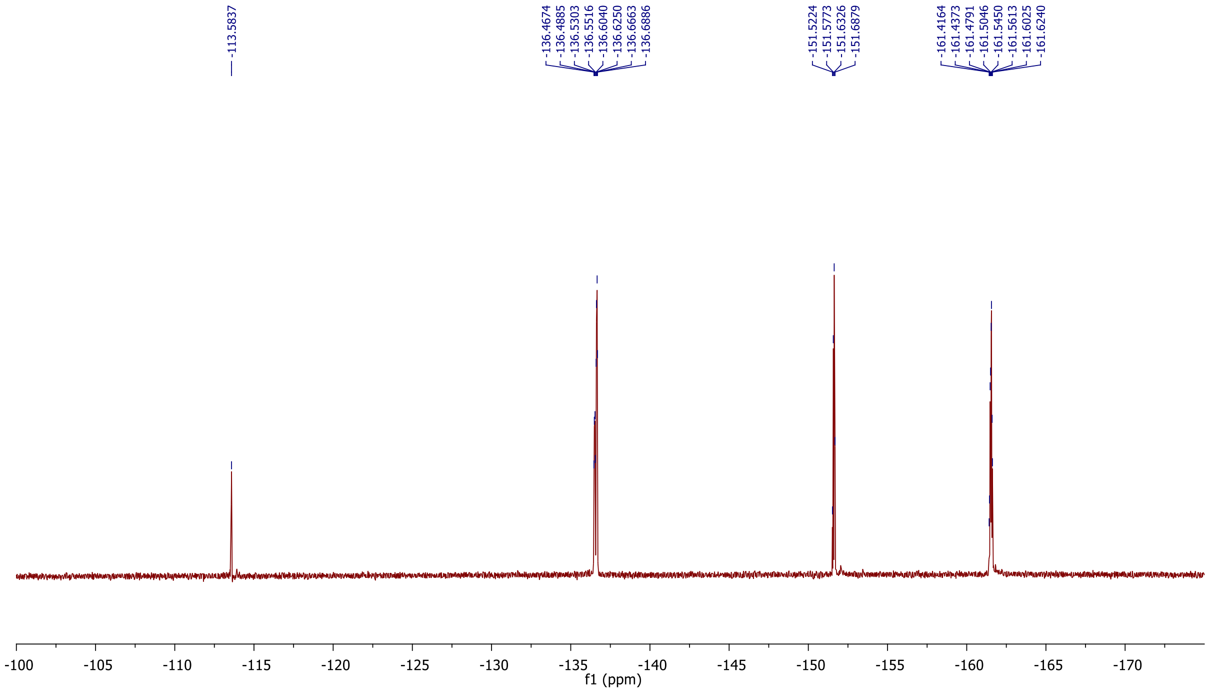

Figure S8. ^{19}**F NMR of 3d (CDCl$_3$)**

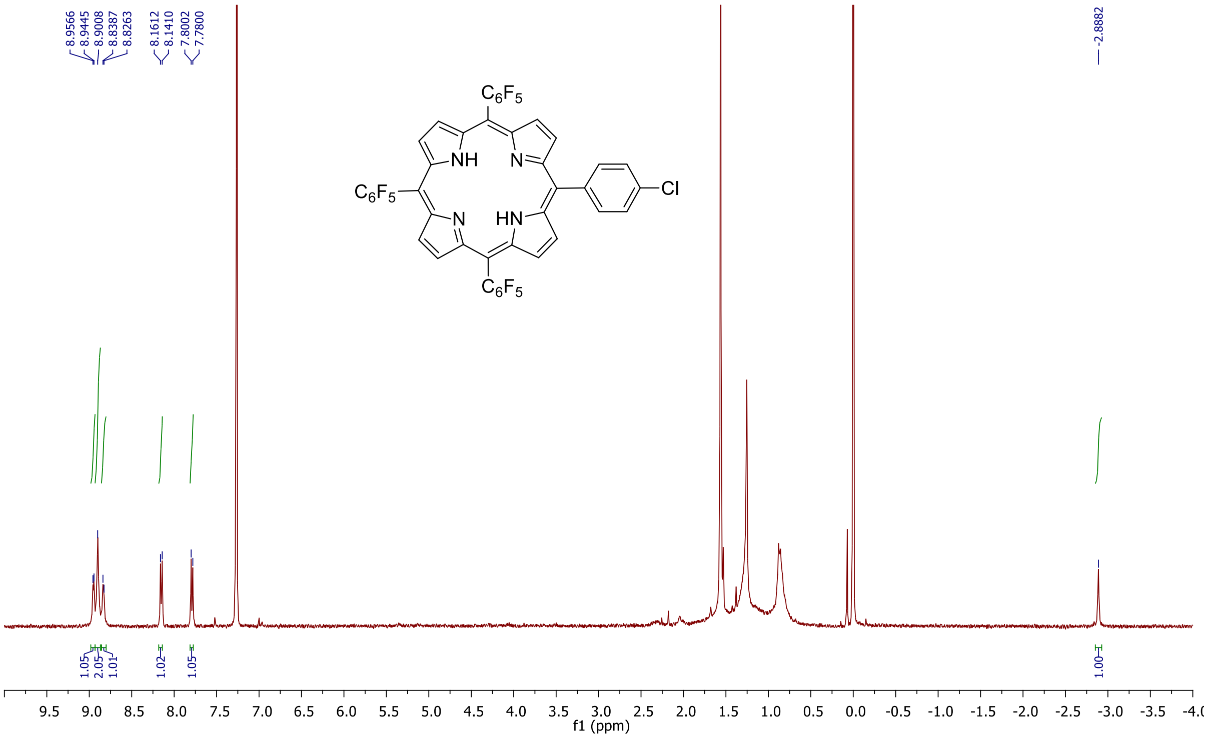

Figure S9. ^{1}H **NMR of 3e ($CDCl_3$)**

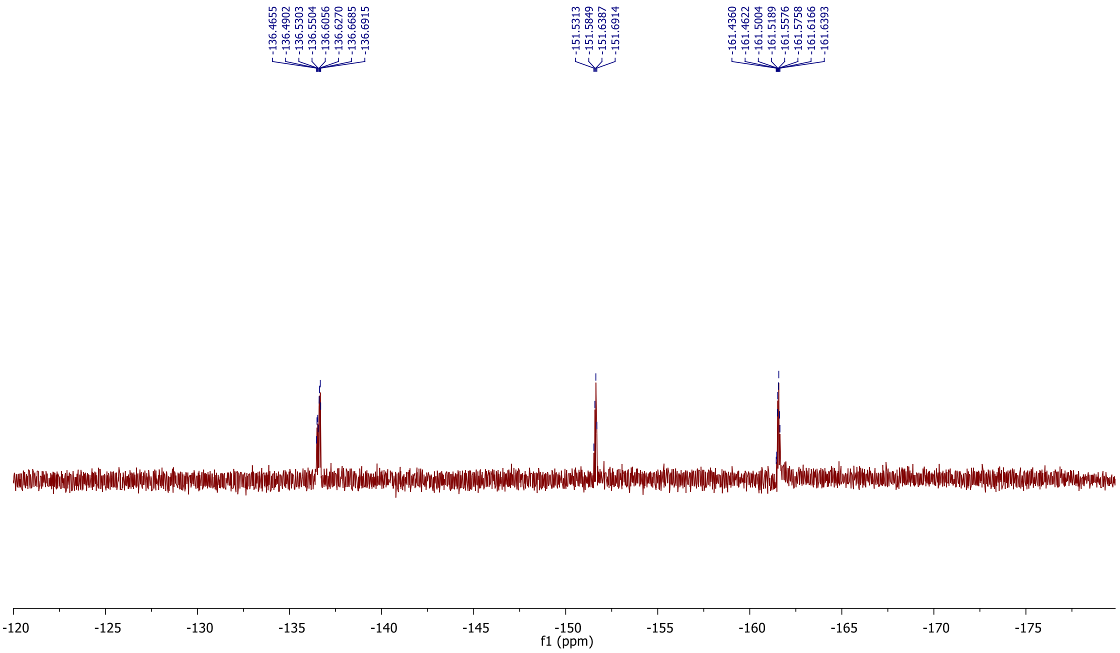

Figure S10. ^{19}F **NMR of 3e ($CDCl_3$)**

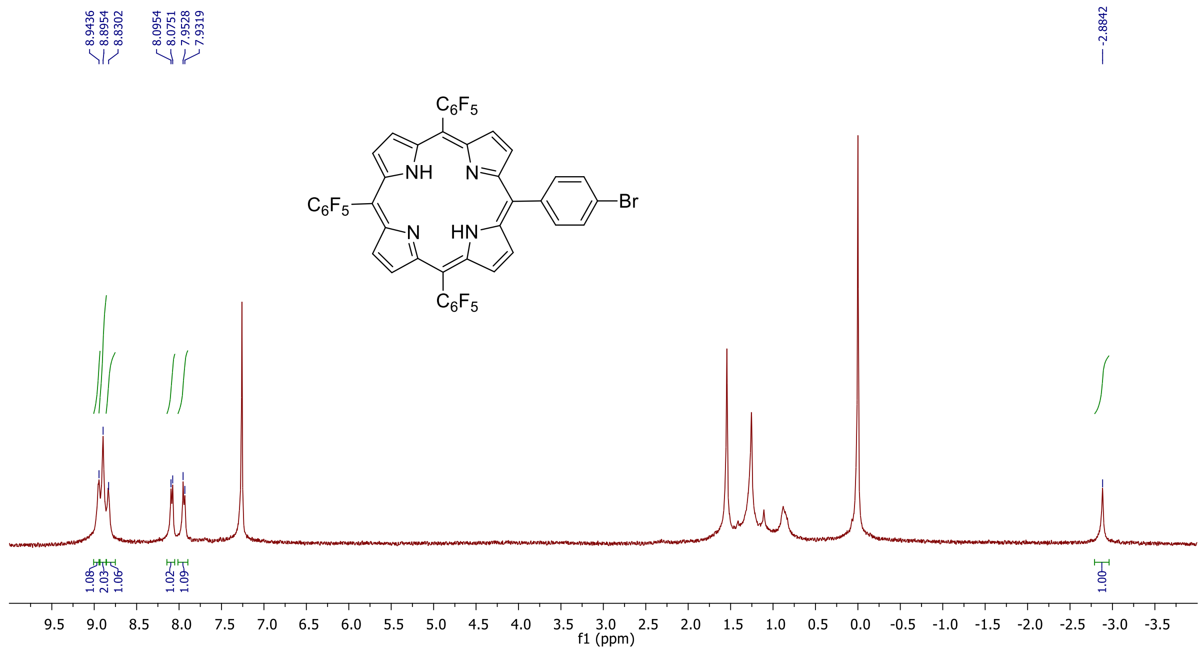

Figure S11. ^{1}H **NMR of 3f (CDCl$_3$)**

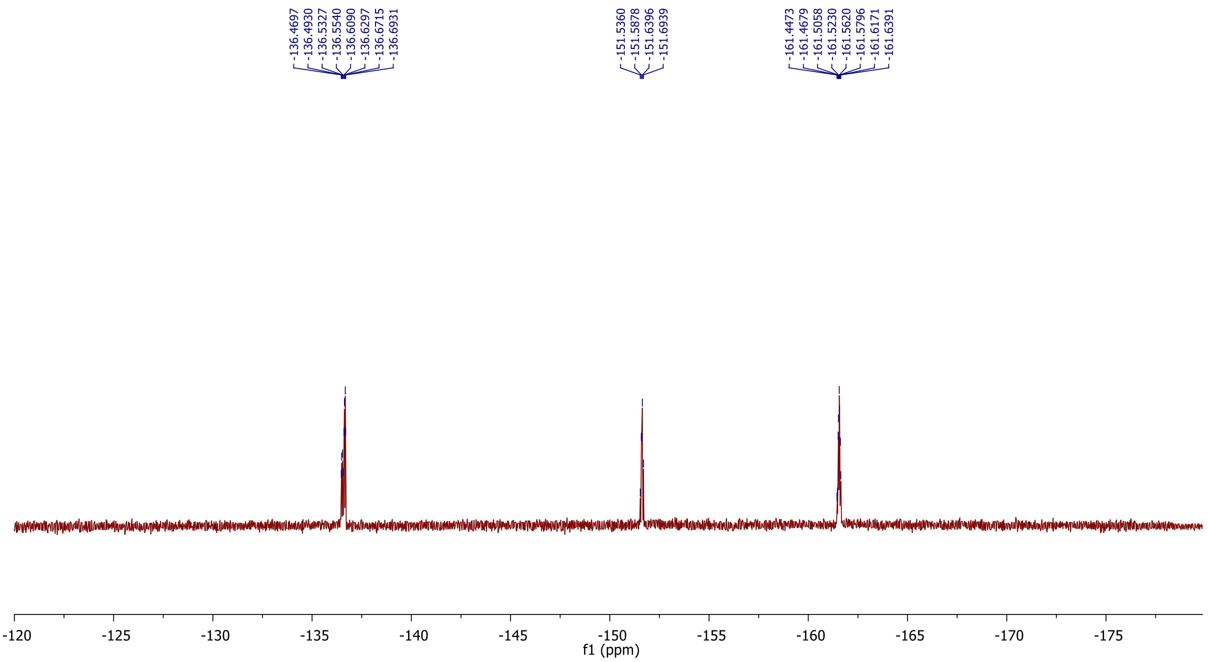

Figure S12. ^{19}F **NMR of 3f (CDCl$_3$)**

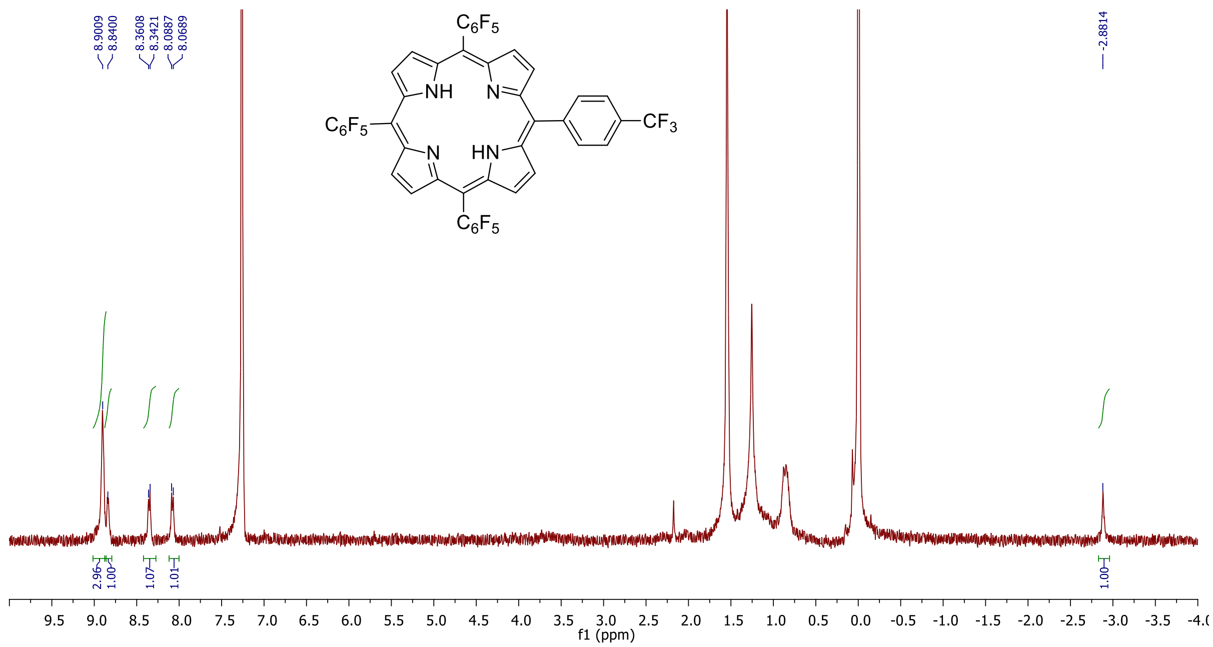

Figure S13. ^{1}H **NMR of 3g ($CDCl_3$)**

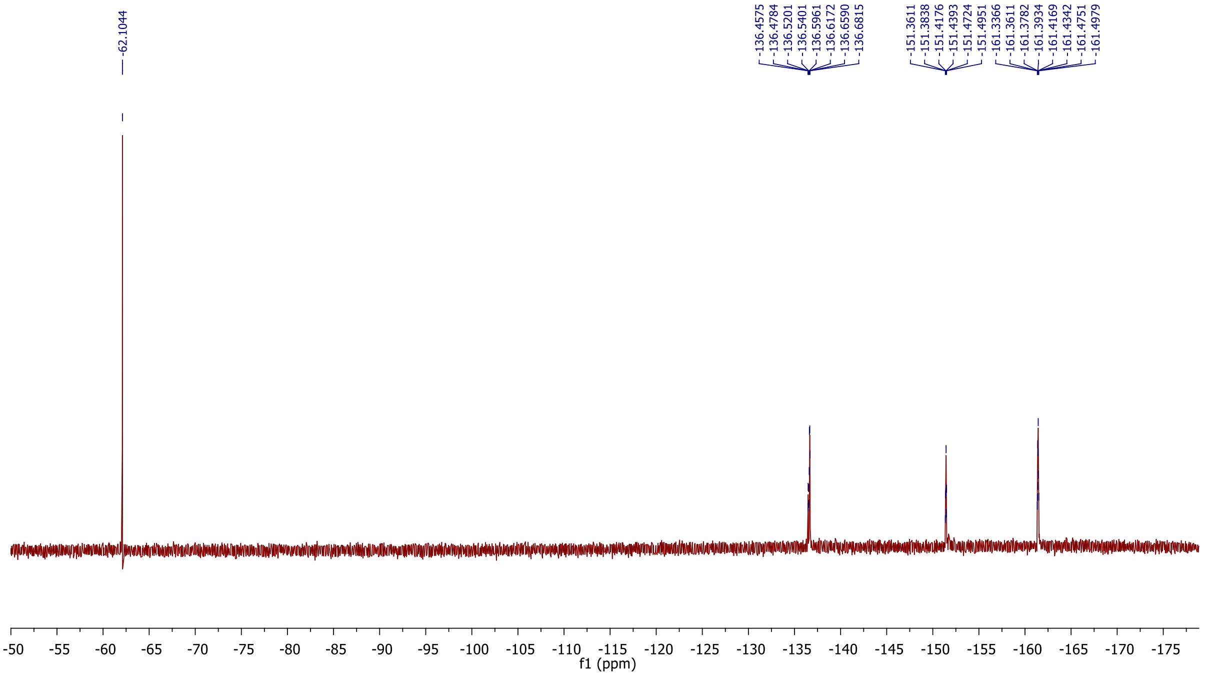

Figure S14. ^{19}F **NMR of 3g ($CDCl_3$)**

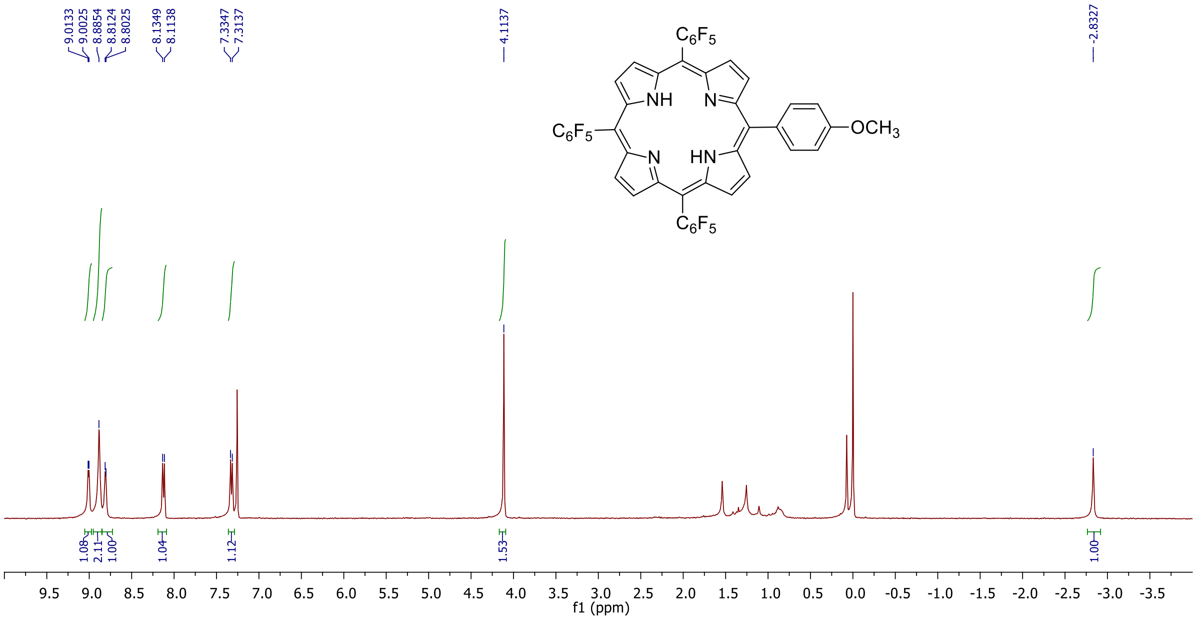

Figure S15. ^{1}H NMR of 3h ($CDCl_3$)

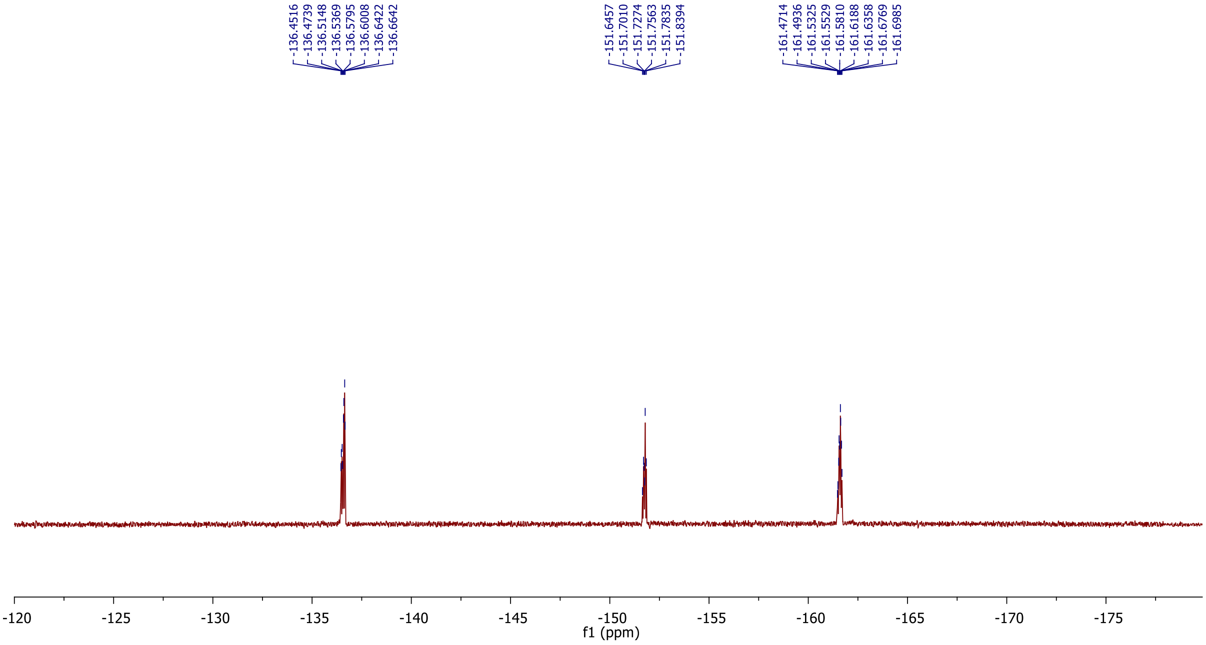

Figure S16. ^{19}F NMR of 3h ($CDCl_3$)

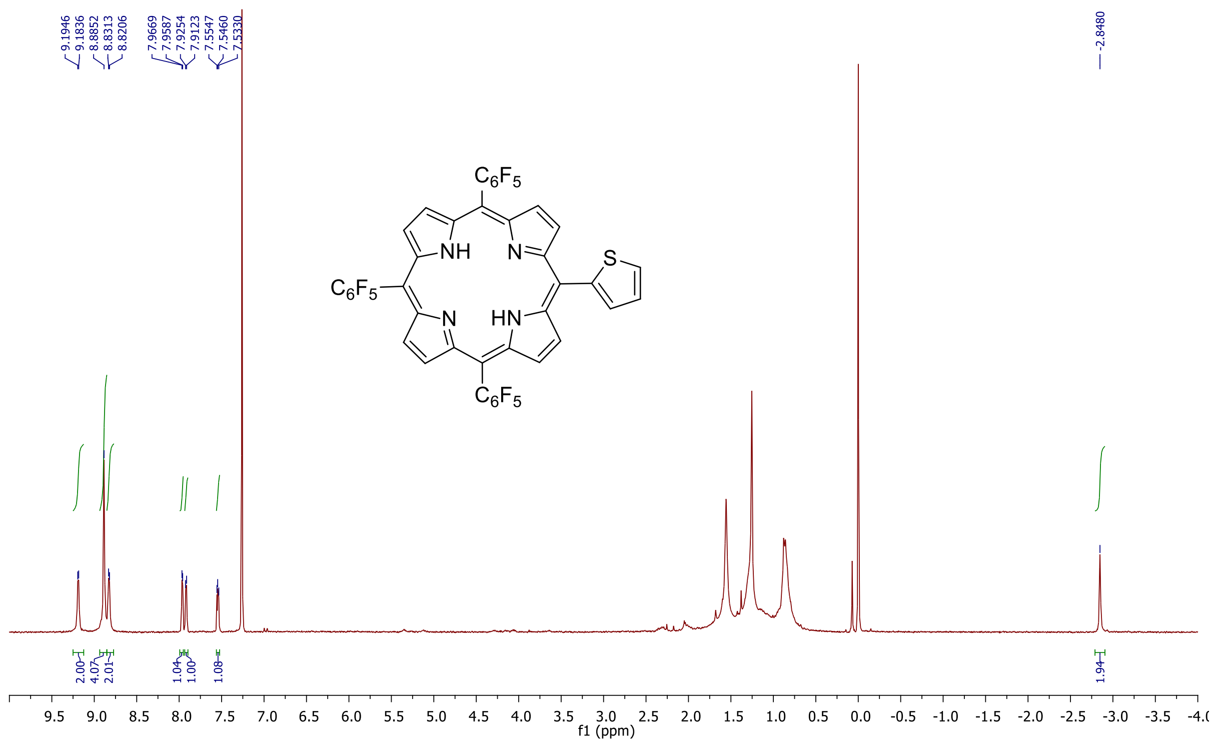

Figure S17. ^{1}H **NMR of 3j ($CDCl_3$)**

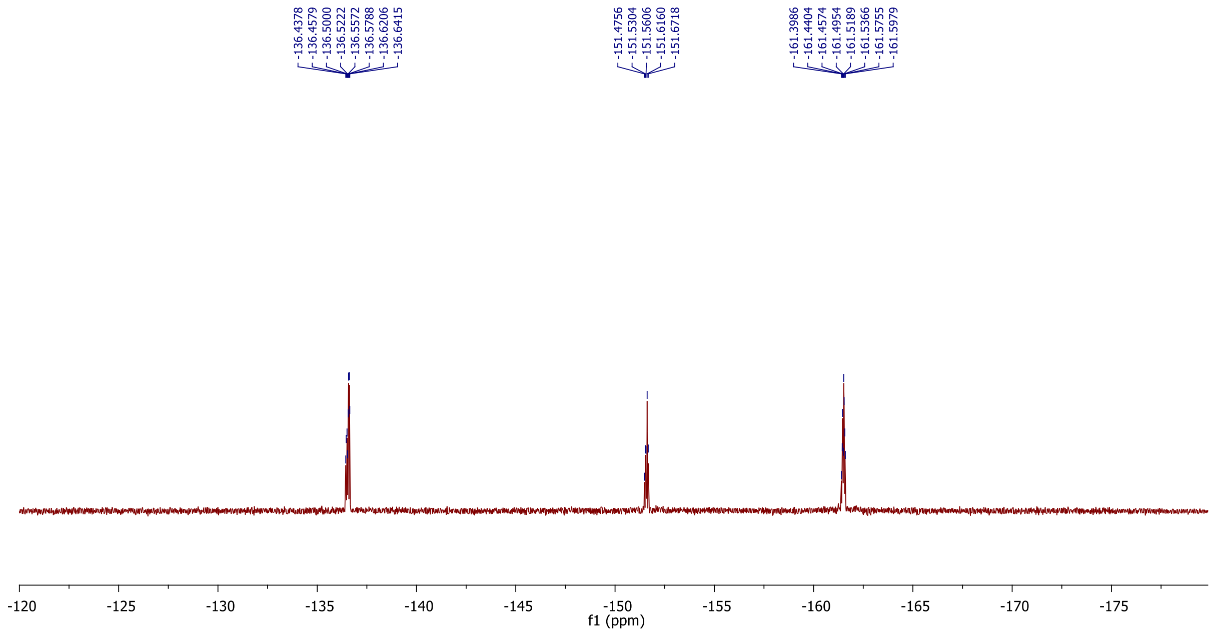

Figure S18. ^{19}F **NMR of 3j ($CDCl_3$)**

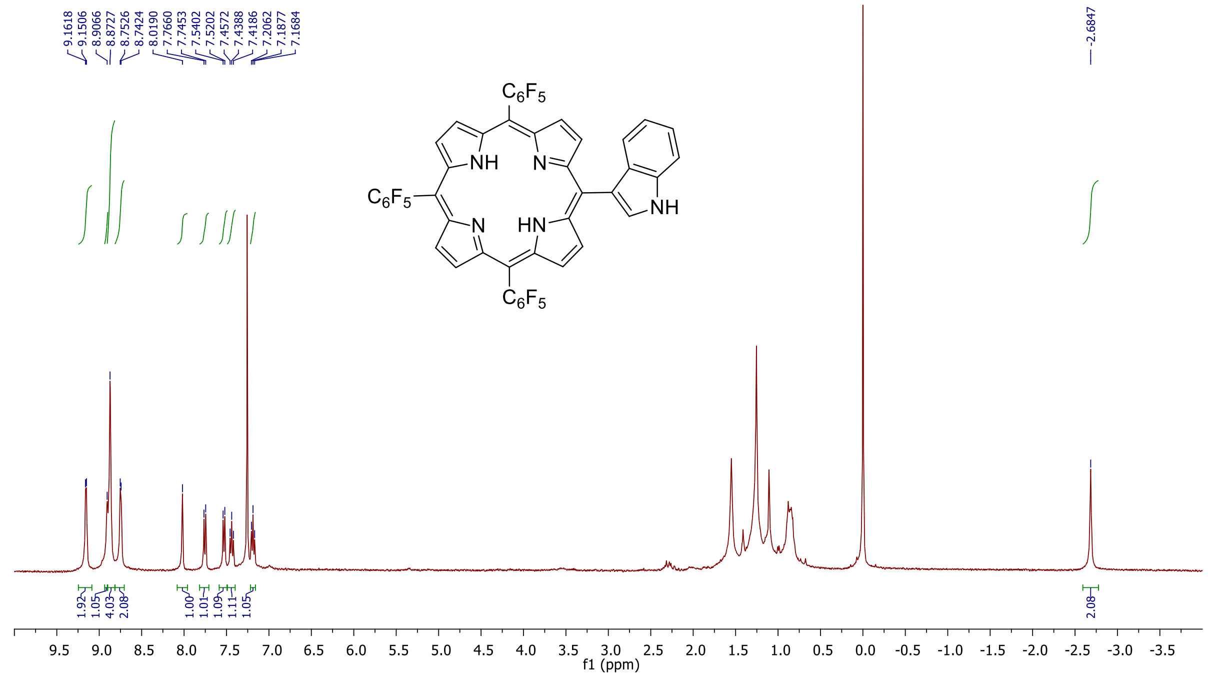

Figure S19. **^{1}H NMR of 3k ($CDCl_3$)**

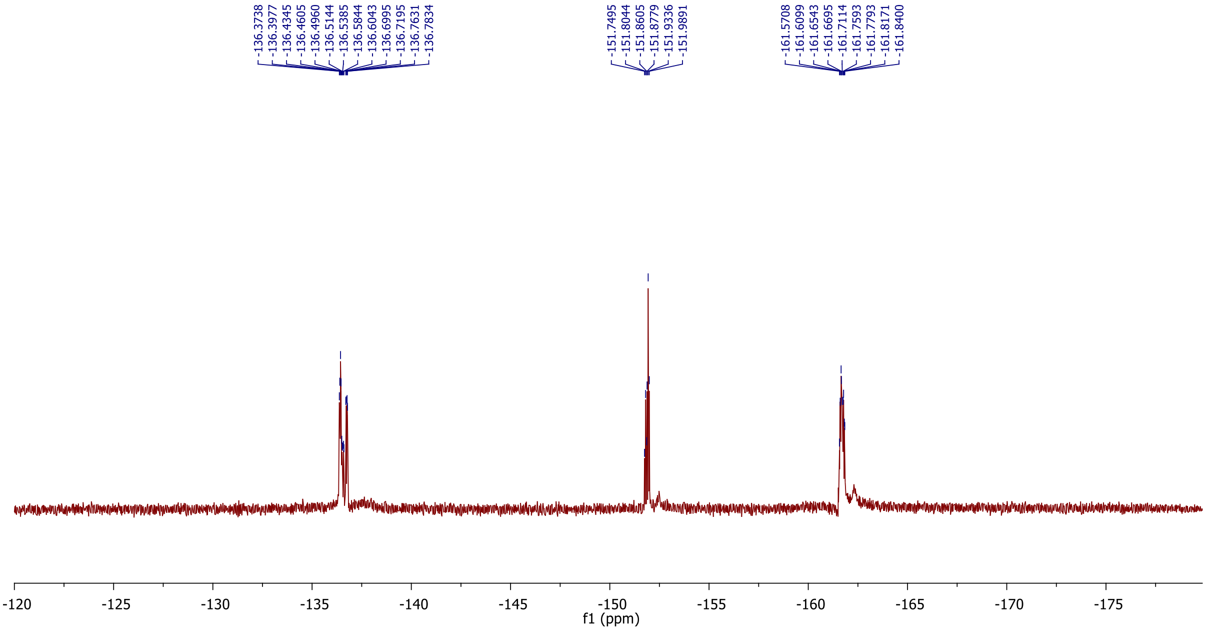

Figure S20. **^{19}F NMR of 3k ($CDCl_3$)**

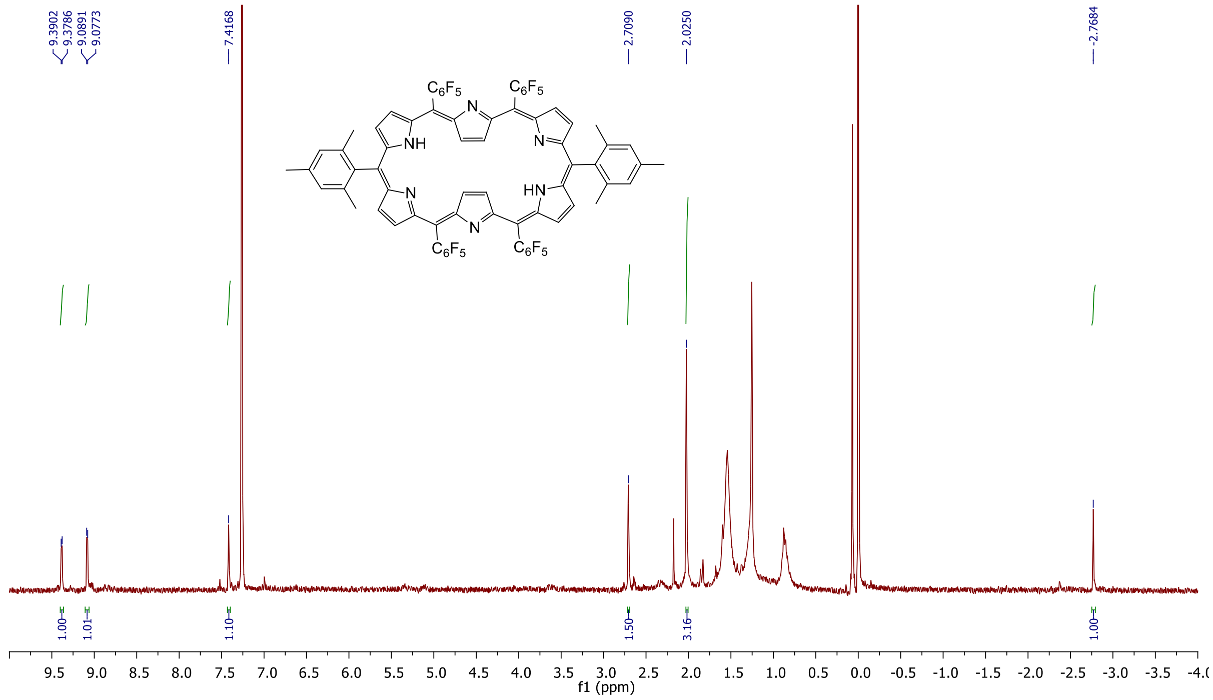

Figure S21. ^{1}**H NMR of 4b (CDCl$_3$)**

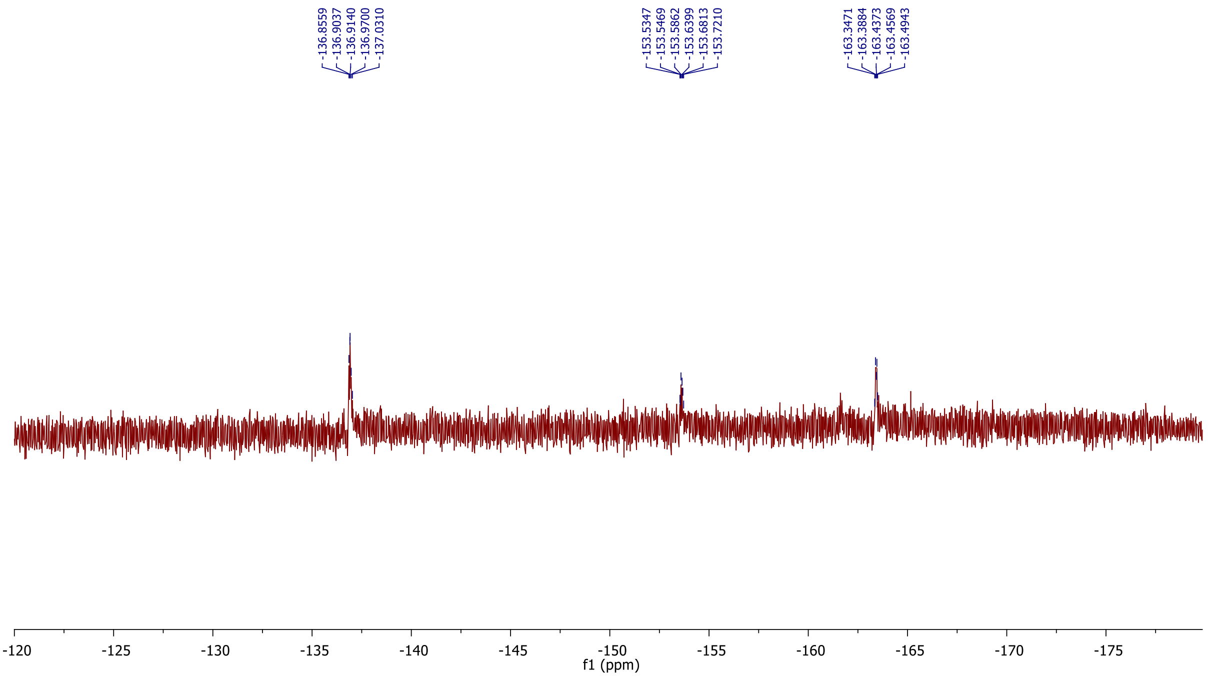

Figure S22. ^{19}**F NMR of 4b (CDCl$_3$)**

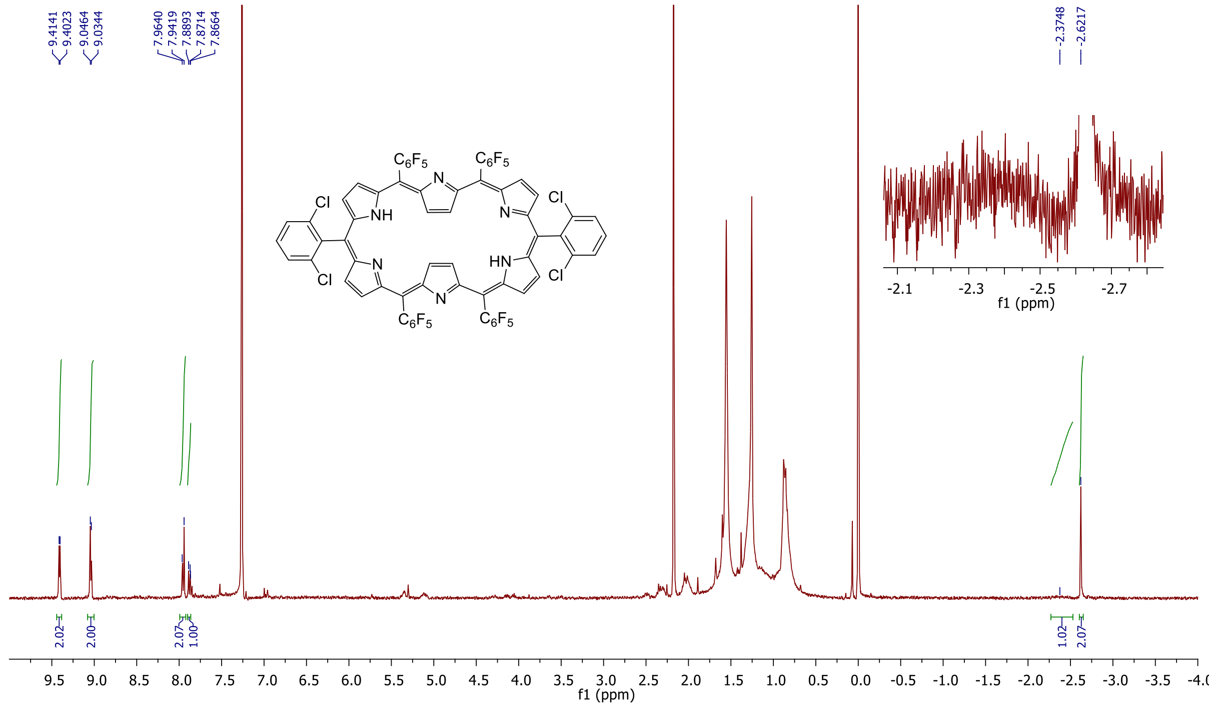

Figure S23. ^{1}H NMR of 4c ($CDCl_3$)

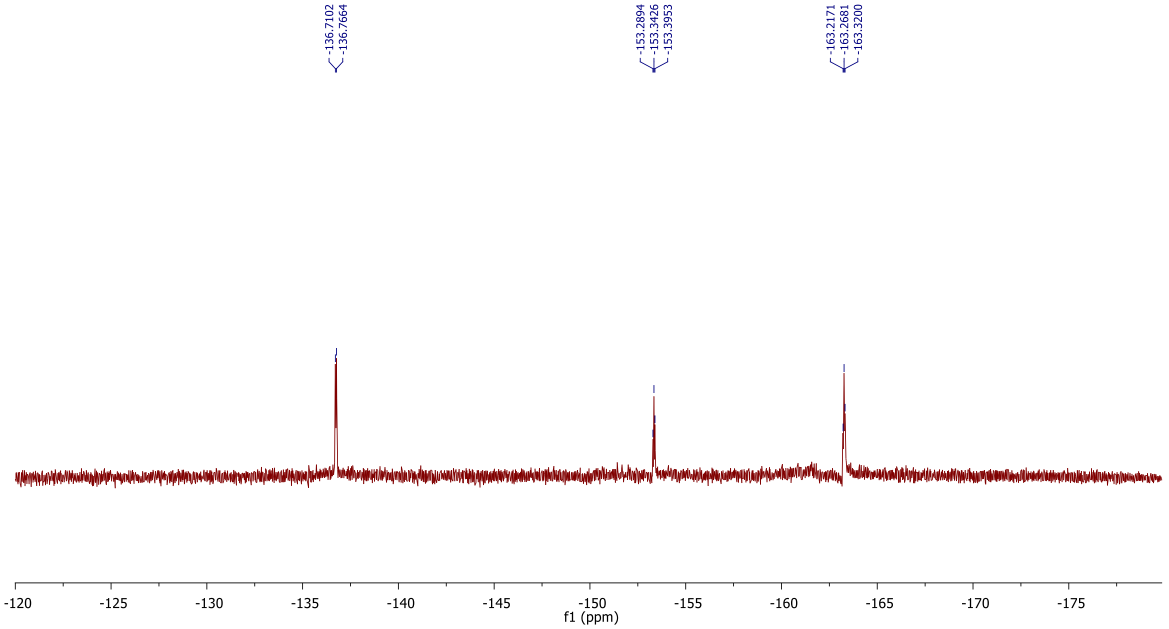

Figure S24. ^{19}F NMR of 4c ($CDCl_3$)

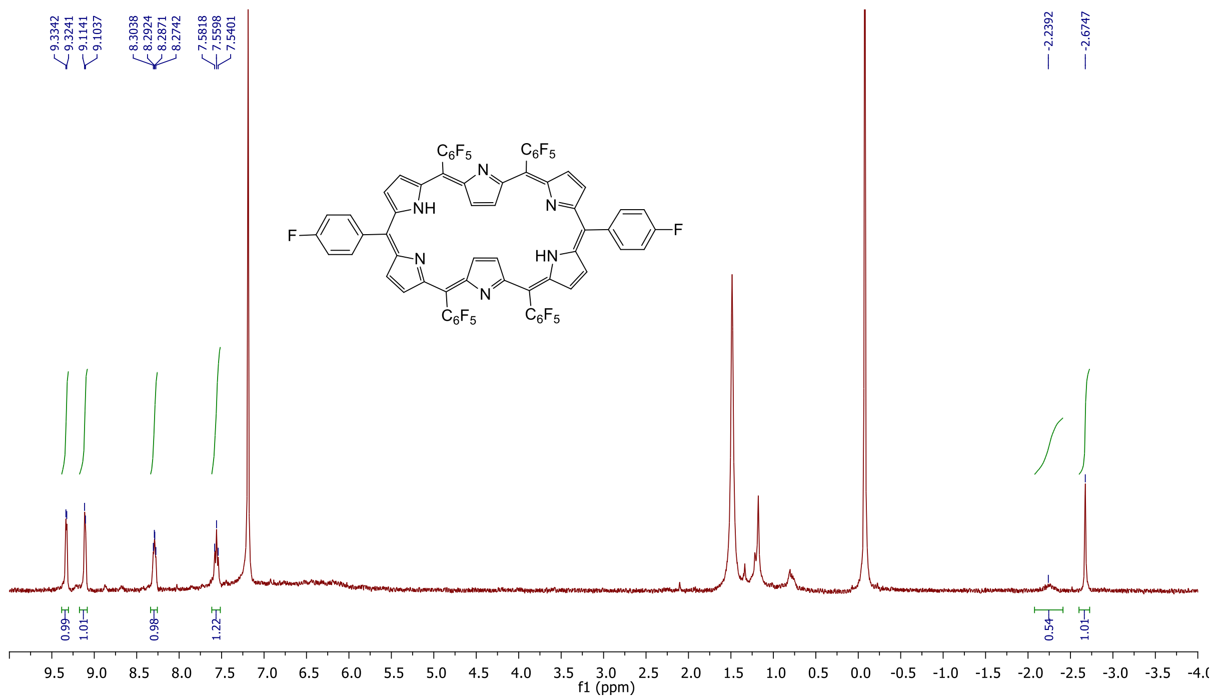

Figure S25. **^{1}H NMR of 4d ($CDCl_3$)**

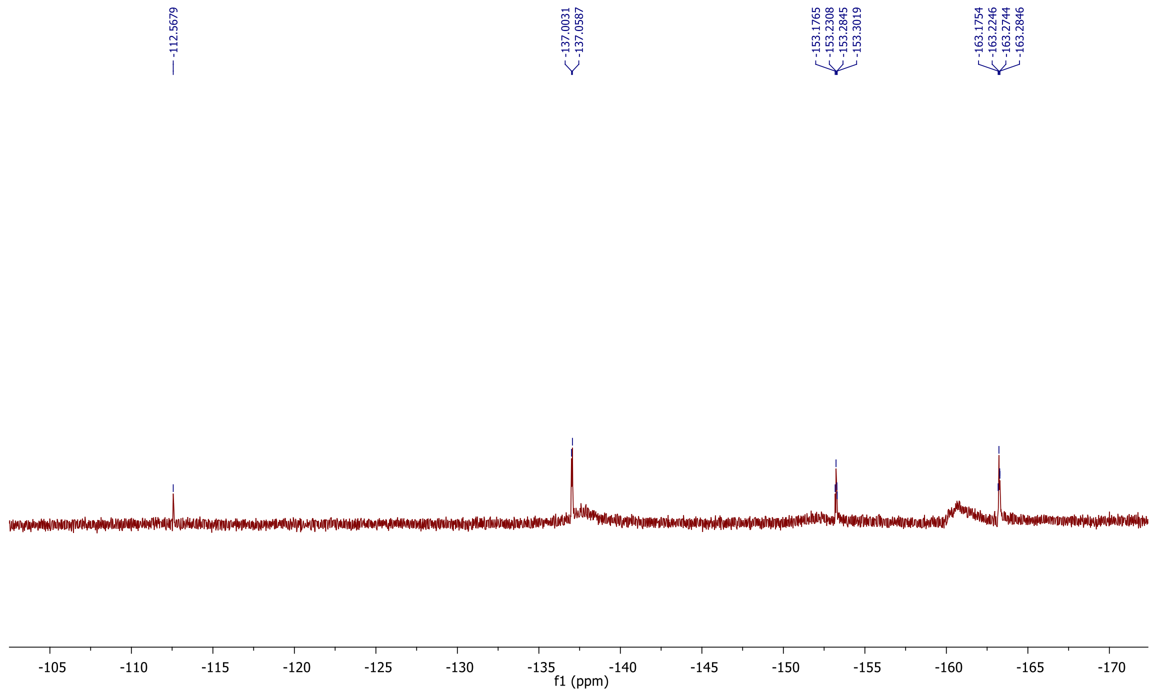

Figure S26. **^{19}F NMR of 4d ($CDCl_3$)**

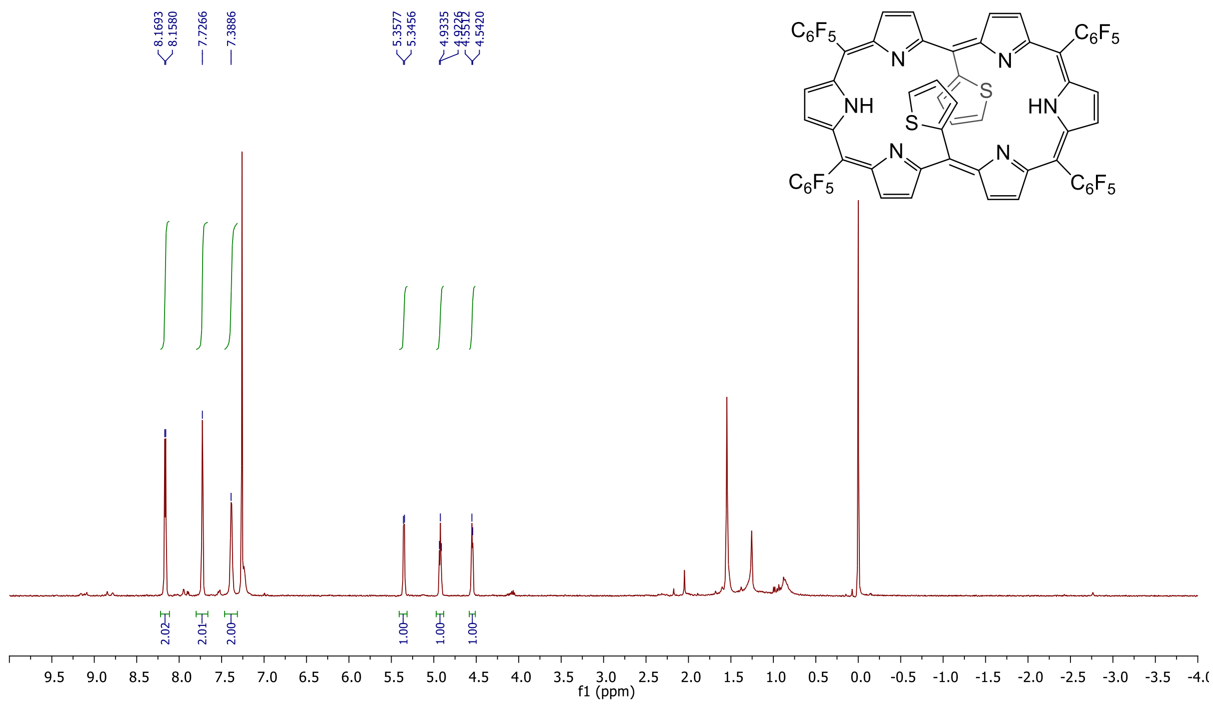

Figure S27. ^{1}H NMR of 4j ($CDCl_3$)

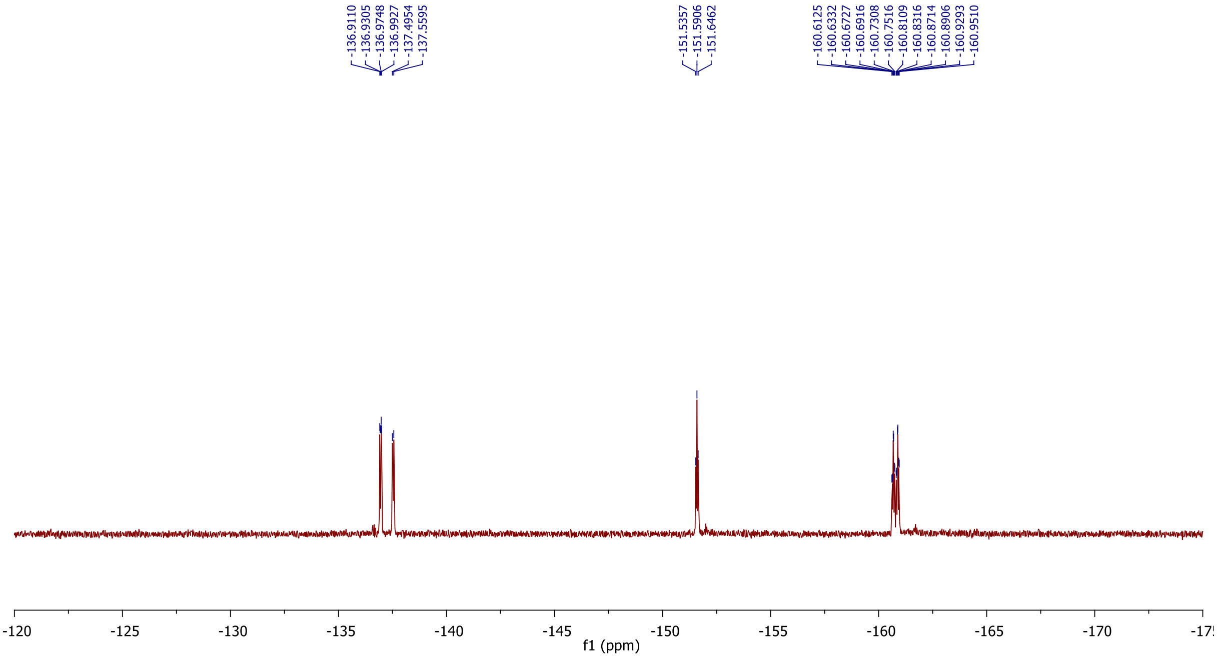

Figure S28. ^{19}F NMR of 4j ($CDCl_3$)

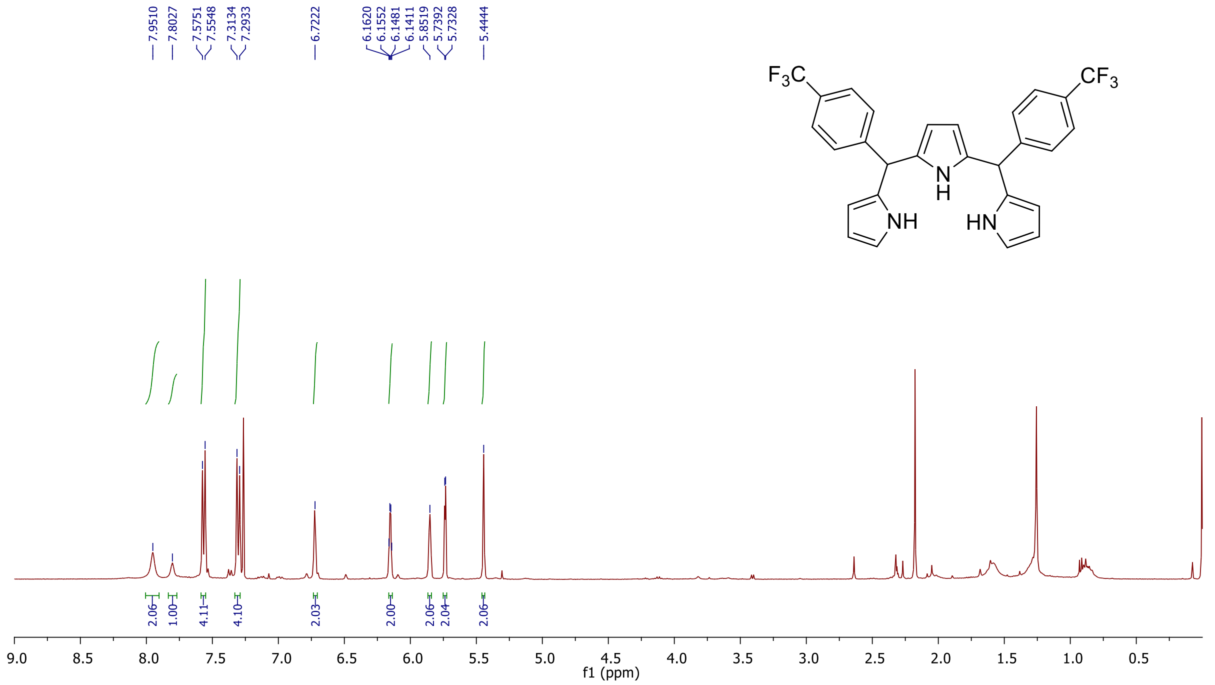

Figure S29. ^{1}H **NMR of 5 ($CDCl_3$)**

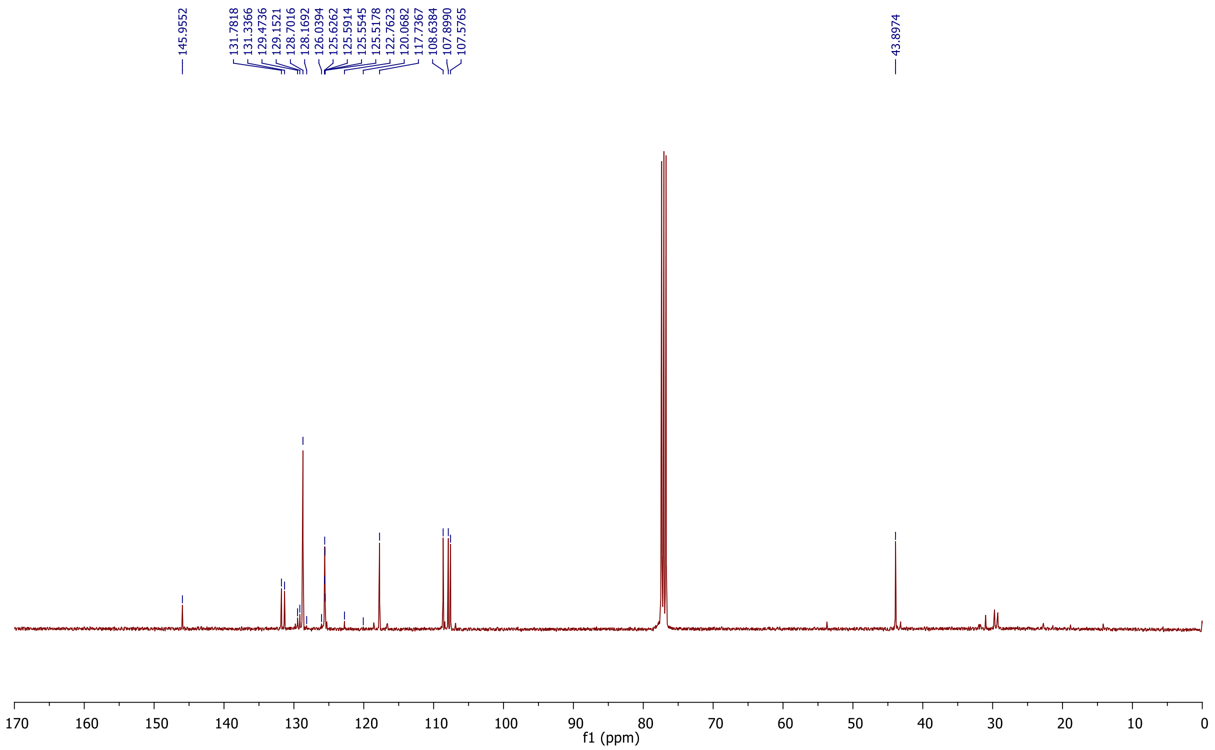

Figure S30. ^{13}C **NMR of 5 ($CDCl_3$)**

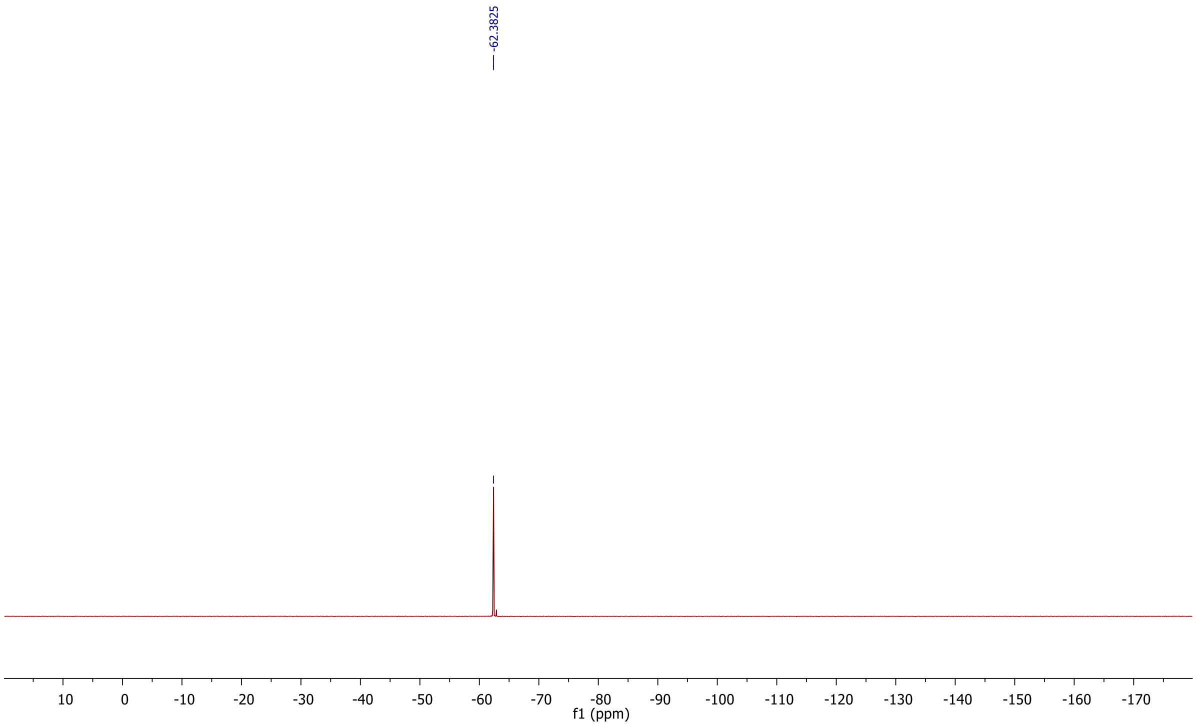

Figure S31. ^{19}F **NMR of 5 ($CDCl_3$)**

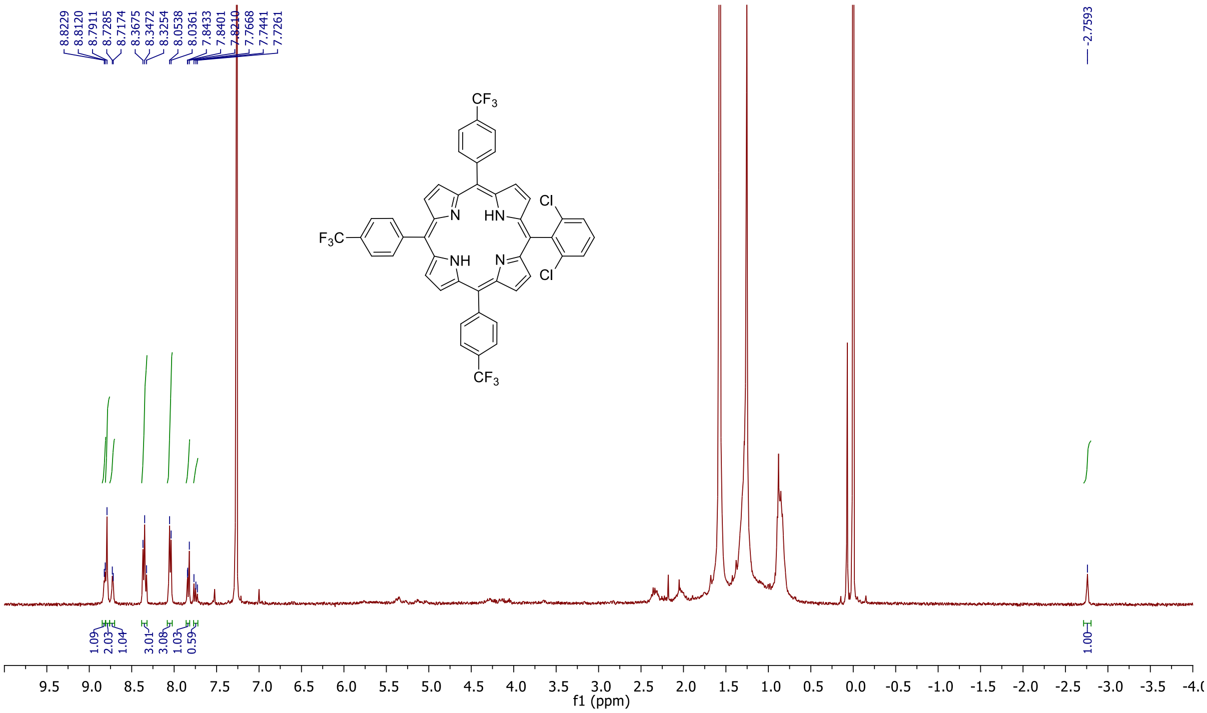

Figure S32. ^{1}**H NMR of 6a (CDCl**$_3$**)**

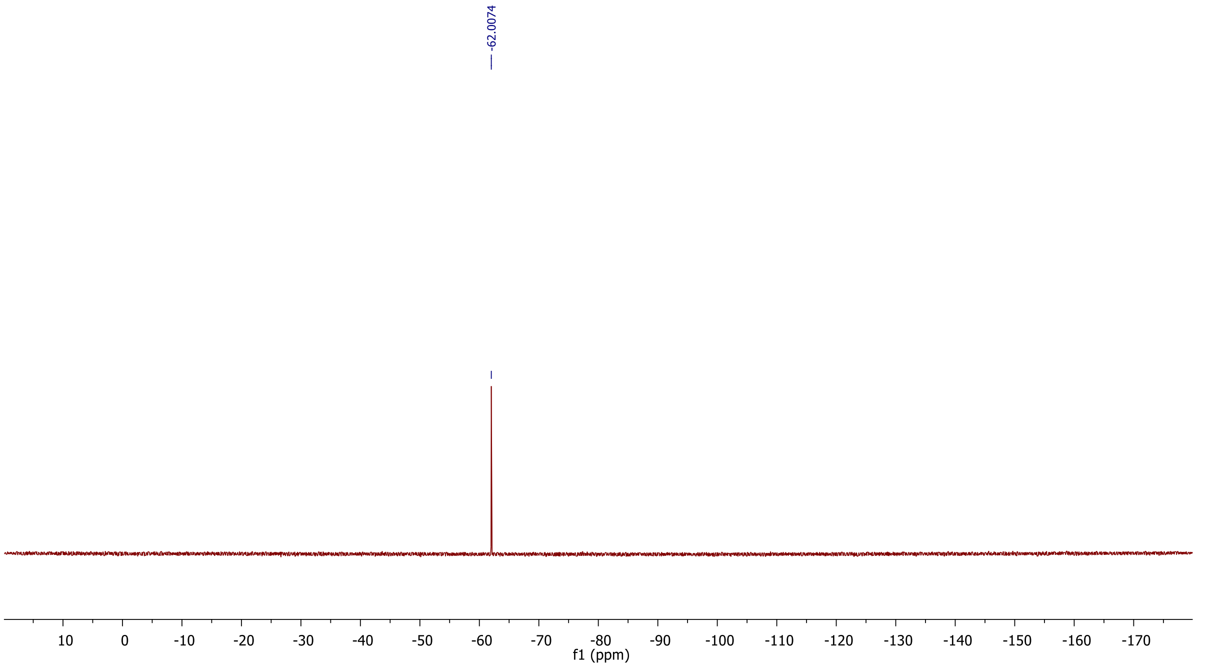

Figure S33. ^{19}**F NMR of 6a (CDCl**$_3$**)**

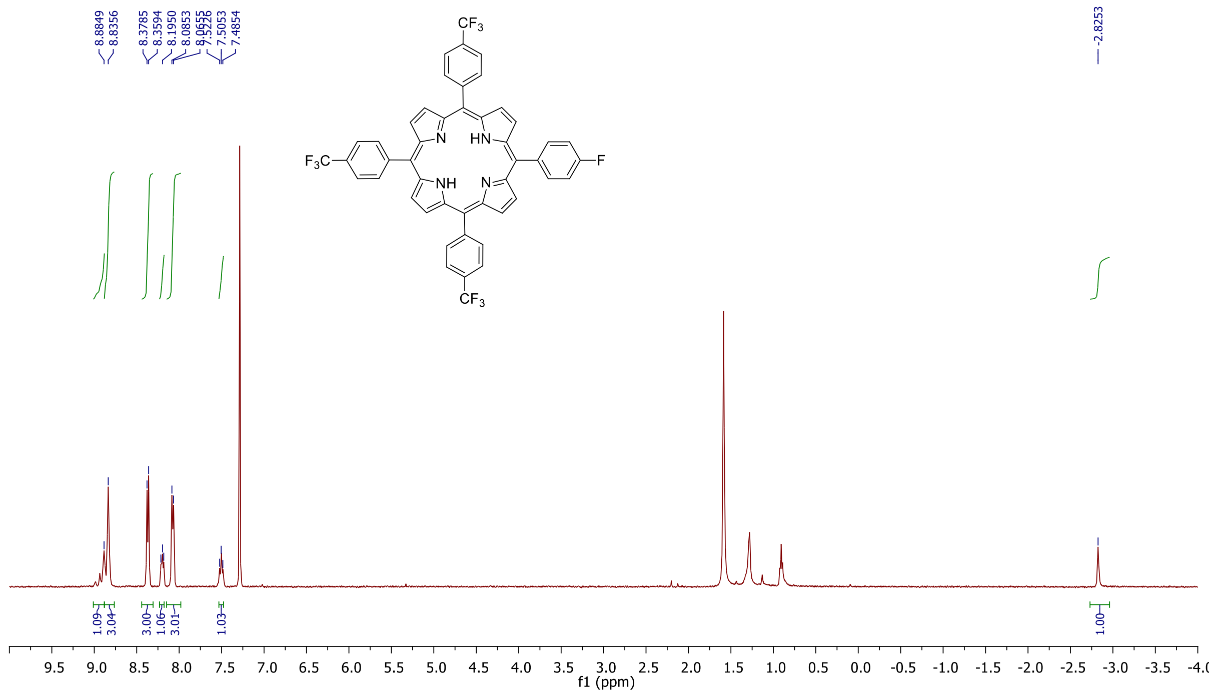

Figure S34. ^{1}H NMR of 6b ($CDCl_3$)

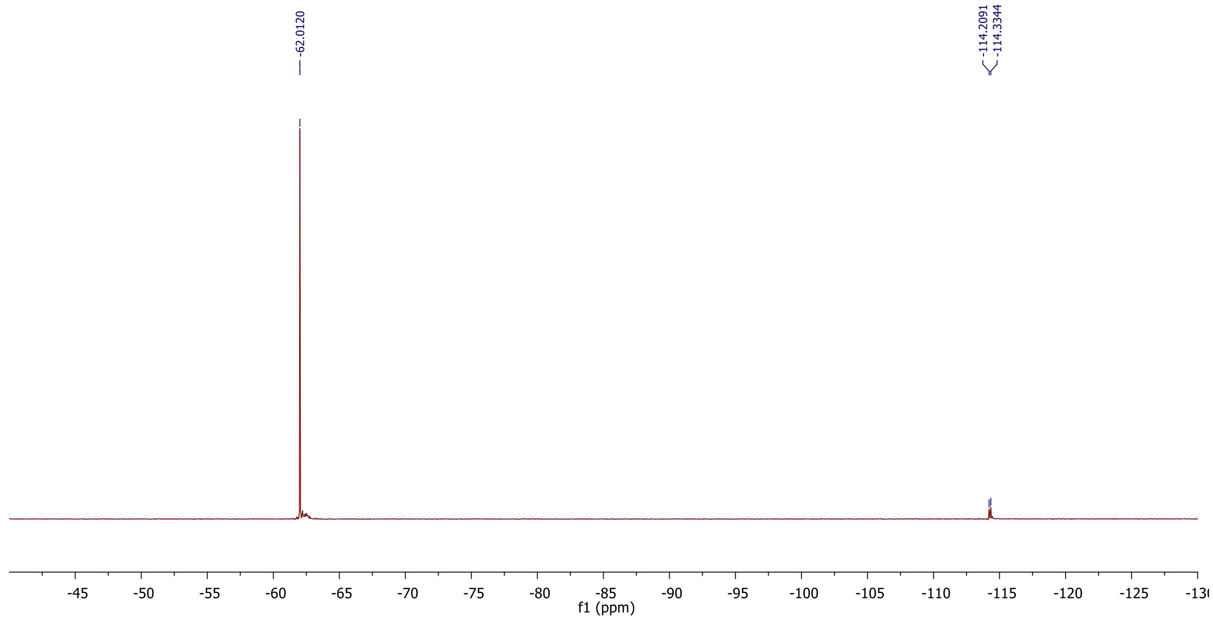

Figure S35. ^{19}F NMR of 6b ($CDCl_3$)

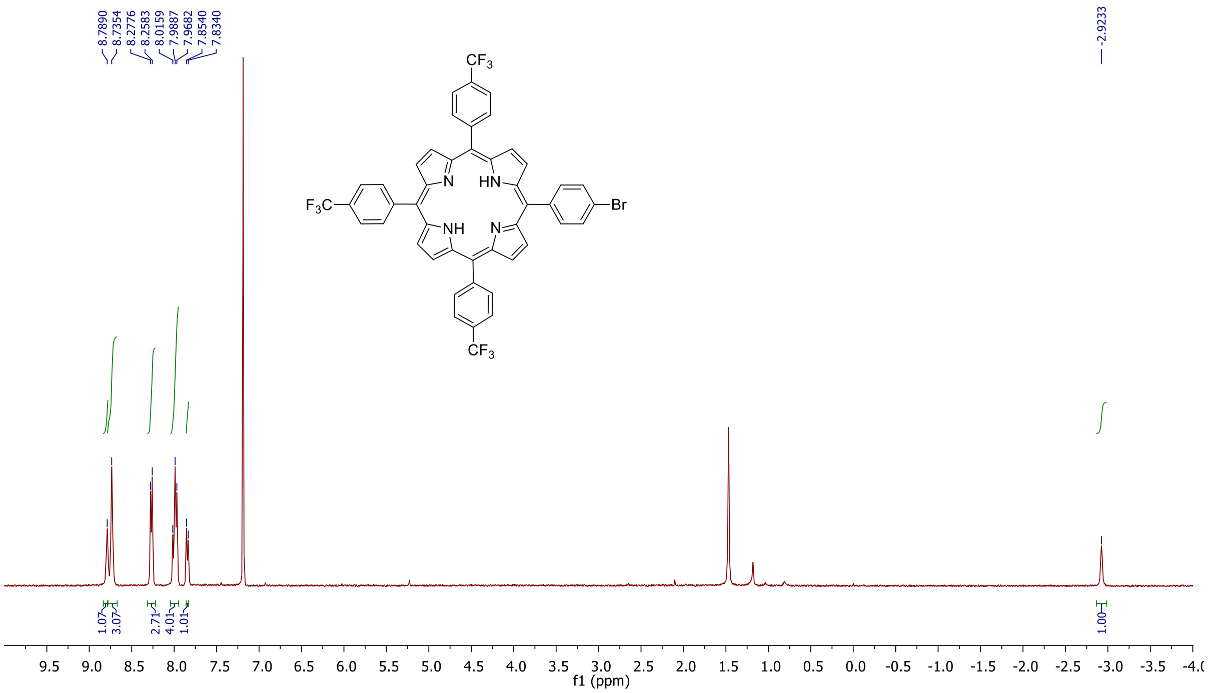

Figure S36. ^{1}**H NMR of 6c (CDCl$_3$)**

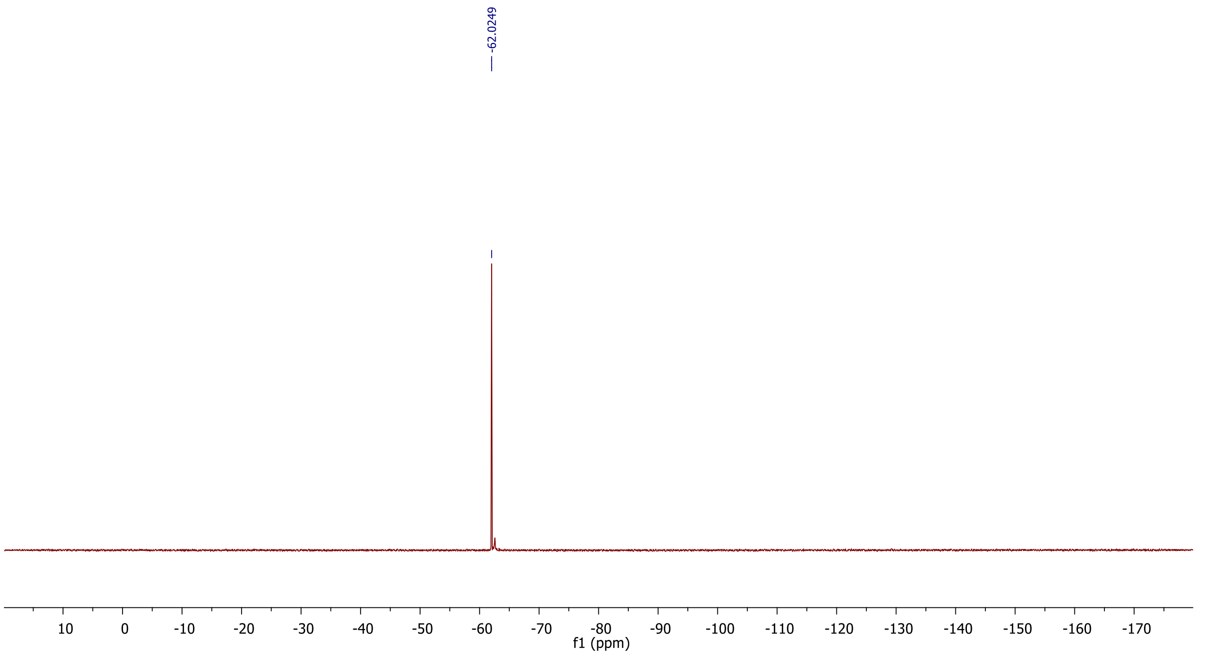

Figure S37. ^{19}**F NMR of 6c (CDCl$_3$)**

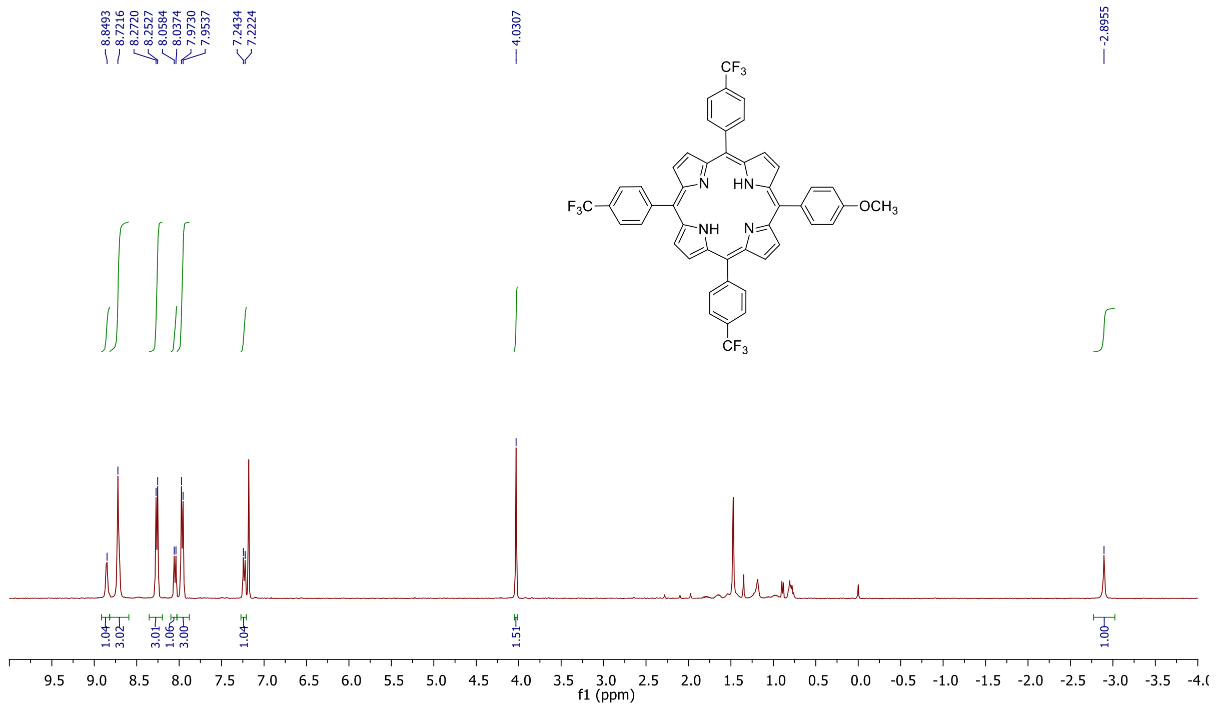

Figure S38. ^{1}H **NMR of 6d ($CDCl_3$)**

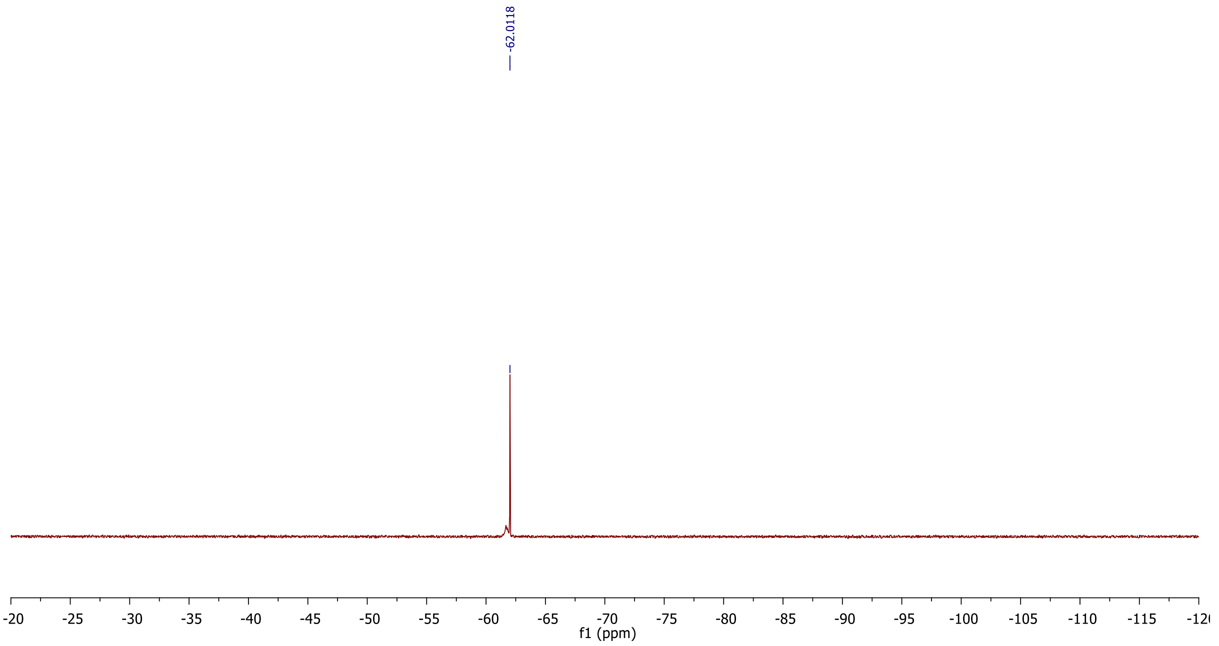

Figure S39. ^{19}F **NMR of 6d ($CDCl_3$)**

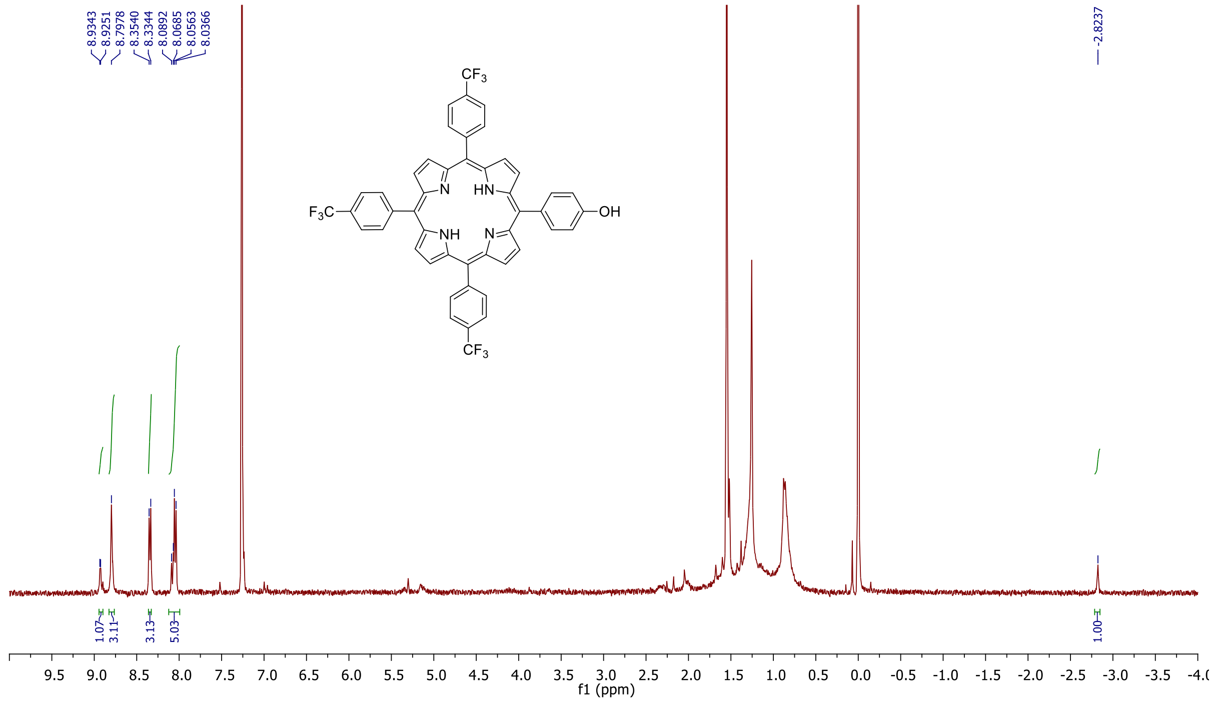

Figure S40. ^{1}H **NMR of 6e ($CDCl_3$)**

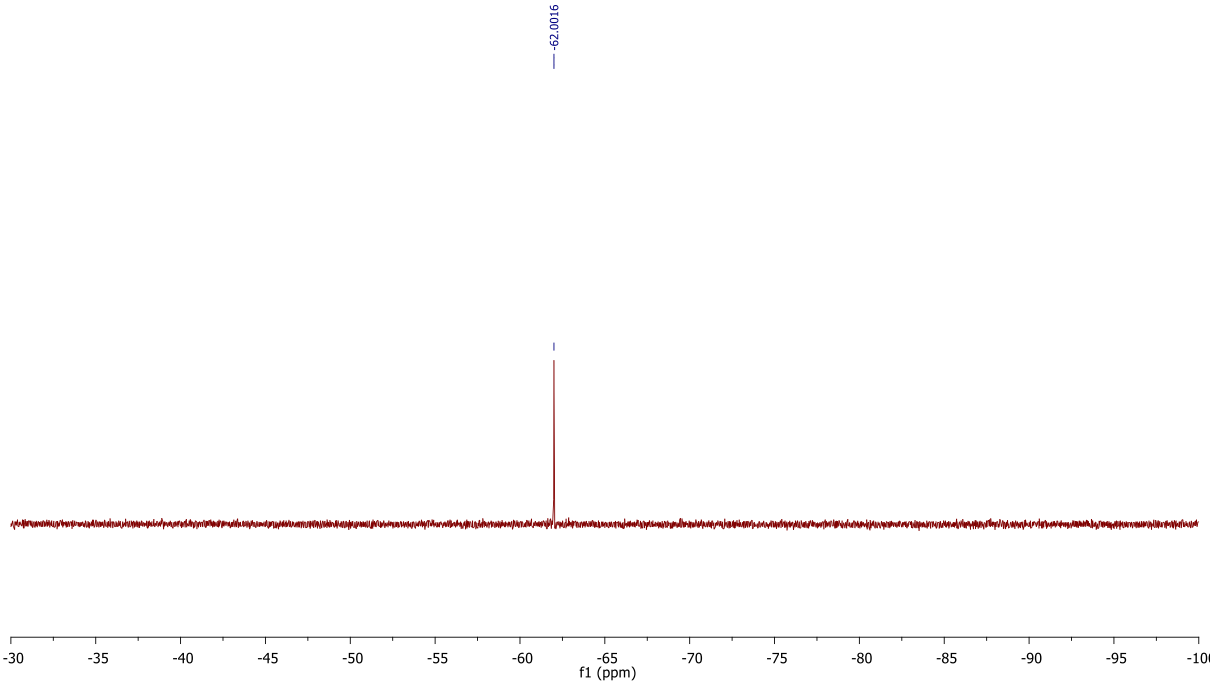

Figure S41. ^{19}F **NMR of 6e ($CDCl_3$)**

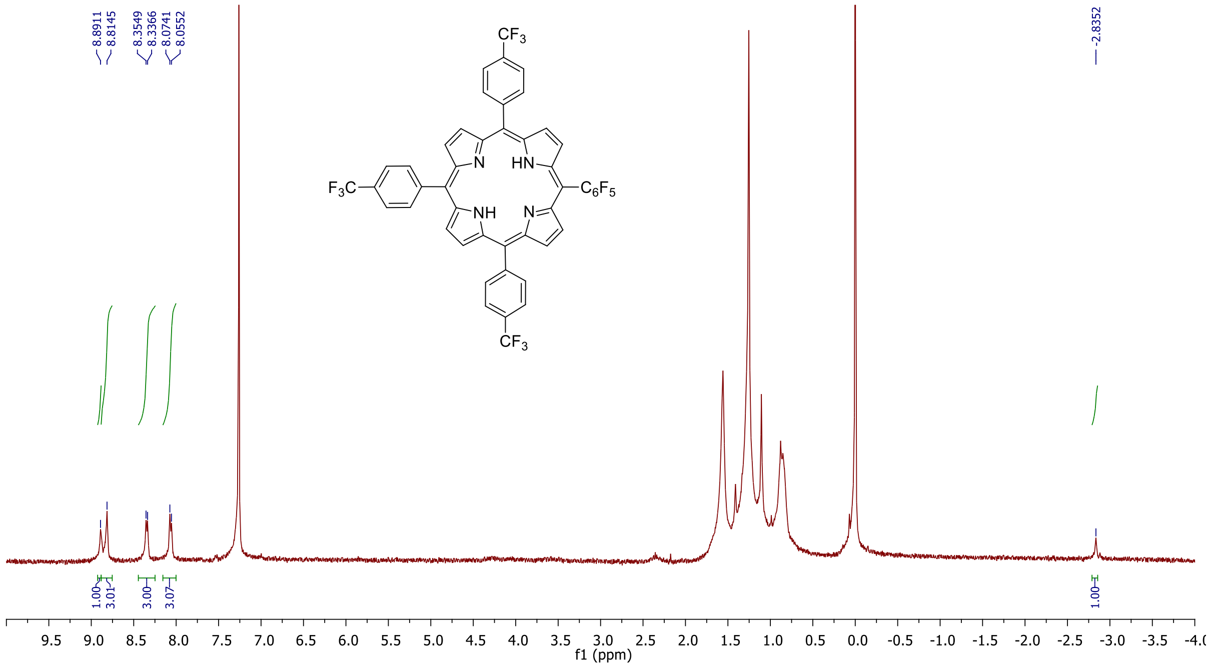

Figure S42. **^{1}H NMR of 6f ($CDCl_3$)**

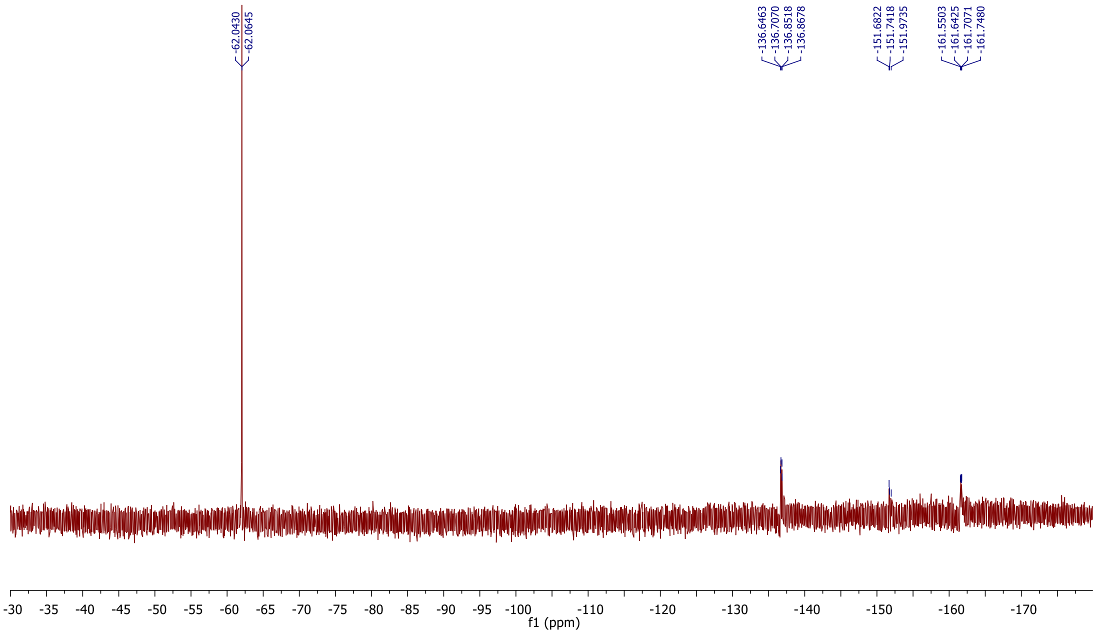

Figure S43. **^{19}F NMR of 6f ($CDCl_3$)**

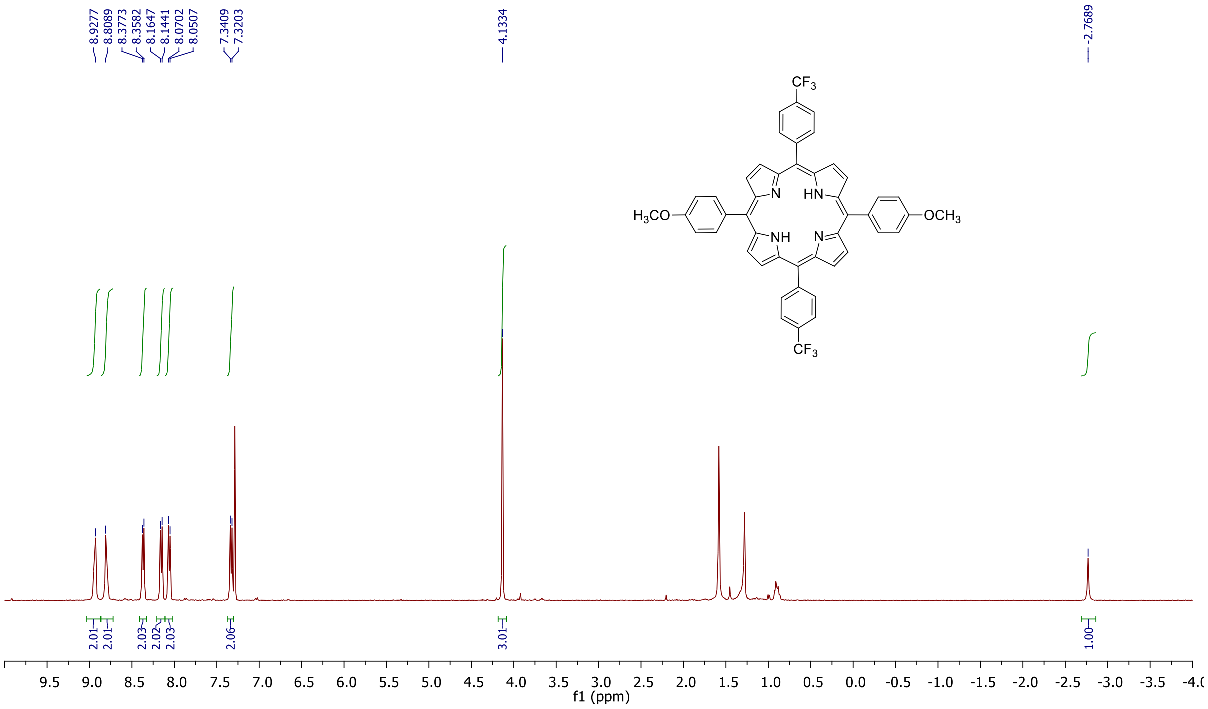

Figure S44. ^{1}H **NMR of 7d ($CDCl_3$)**

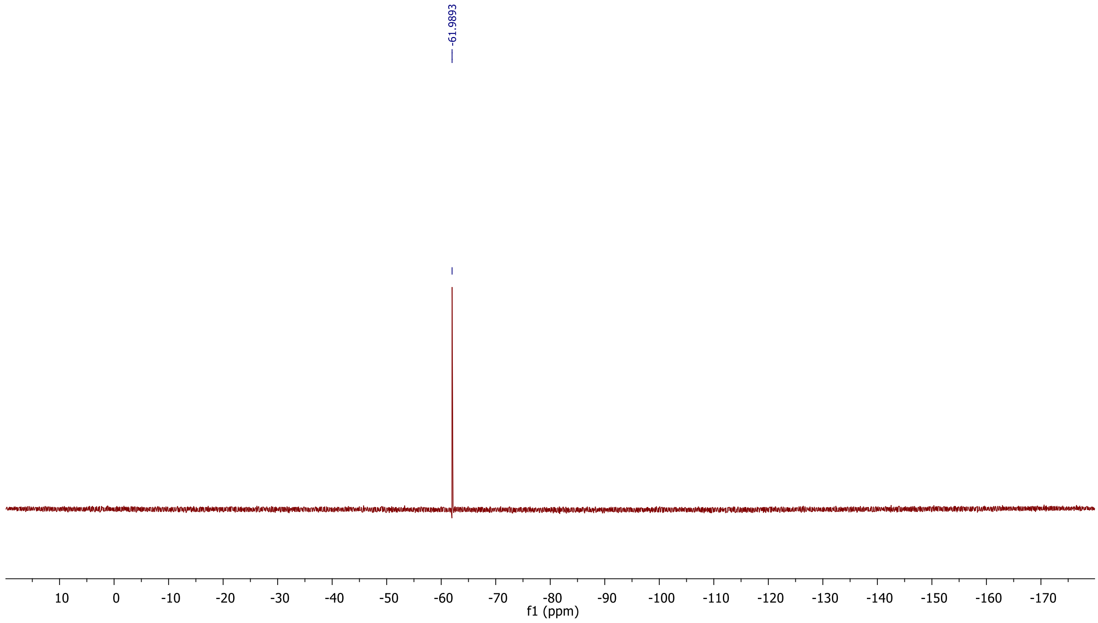

Figure S45. ^{19}F **NMR of 7d ($CDCl_3$)**

5. LC MS-TOF (ESI) spectra of reaction intermediates

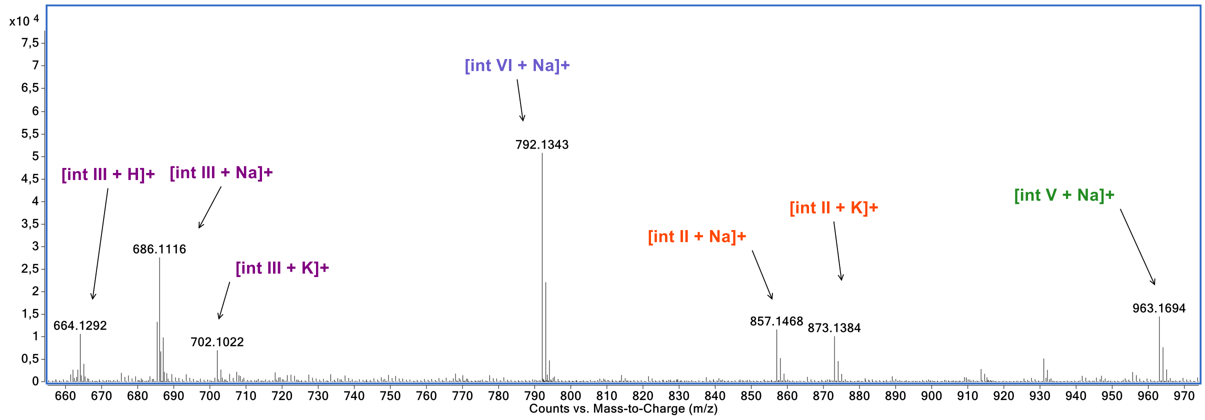

Figure S46. Intermediates **II**, **III**, **V** and **VI** in **1** and **2d** reaction mixture at 0 °C for 2 hours

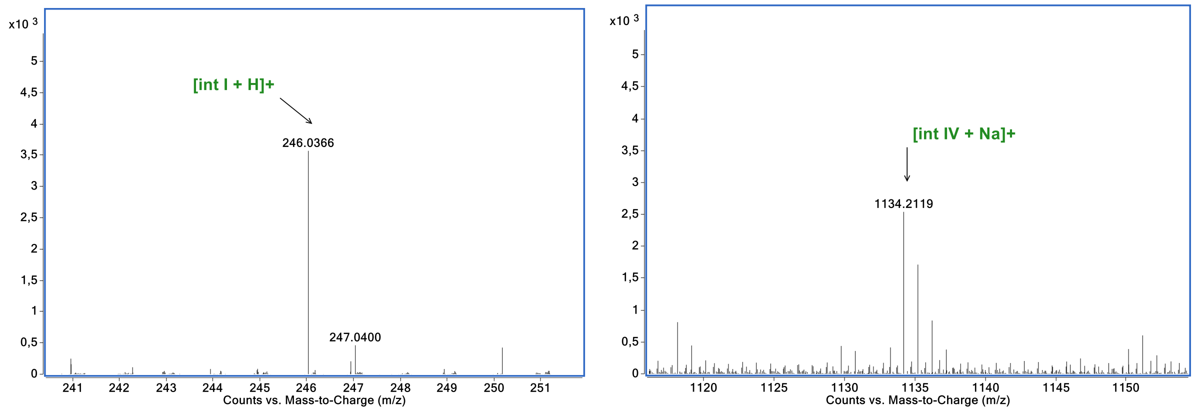

Figure S47. Intermediates **I** and **IV** in **1** and **2d** reaction mixture at 0 °C for 2 hours

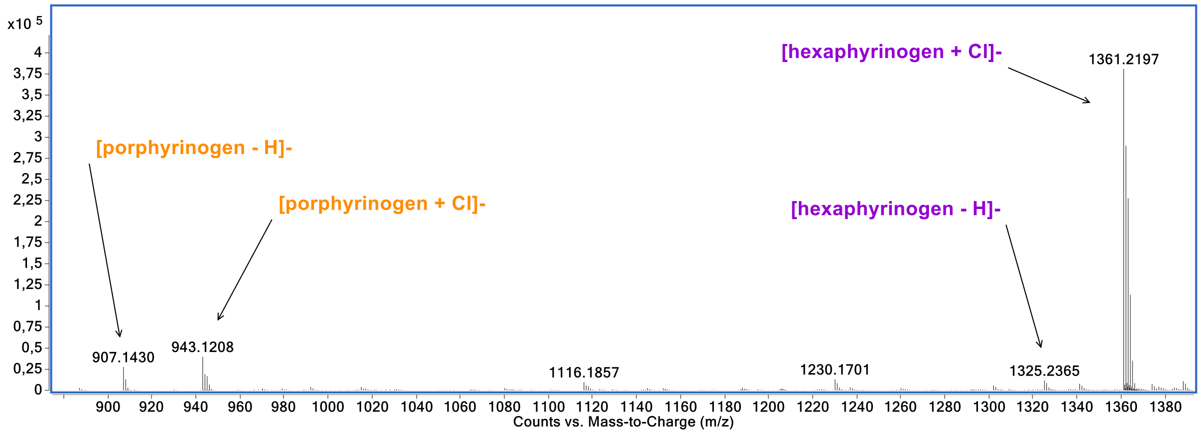

Figure S48. Porphyrinogen and hexaphyrinogen of **1** and **2d** reaction mixture at r.t. for 1 hour

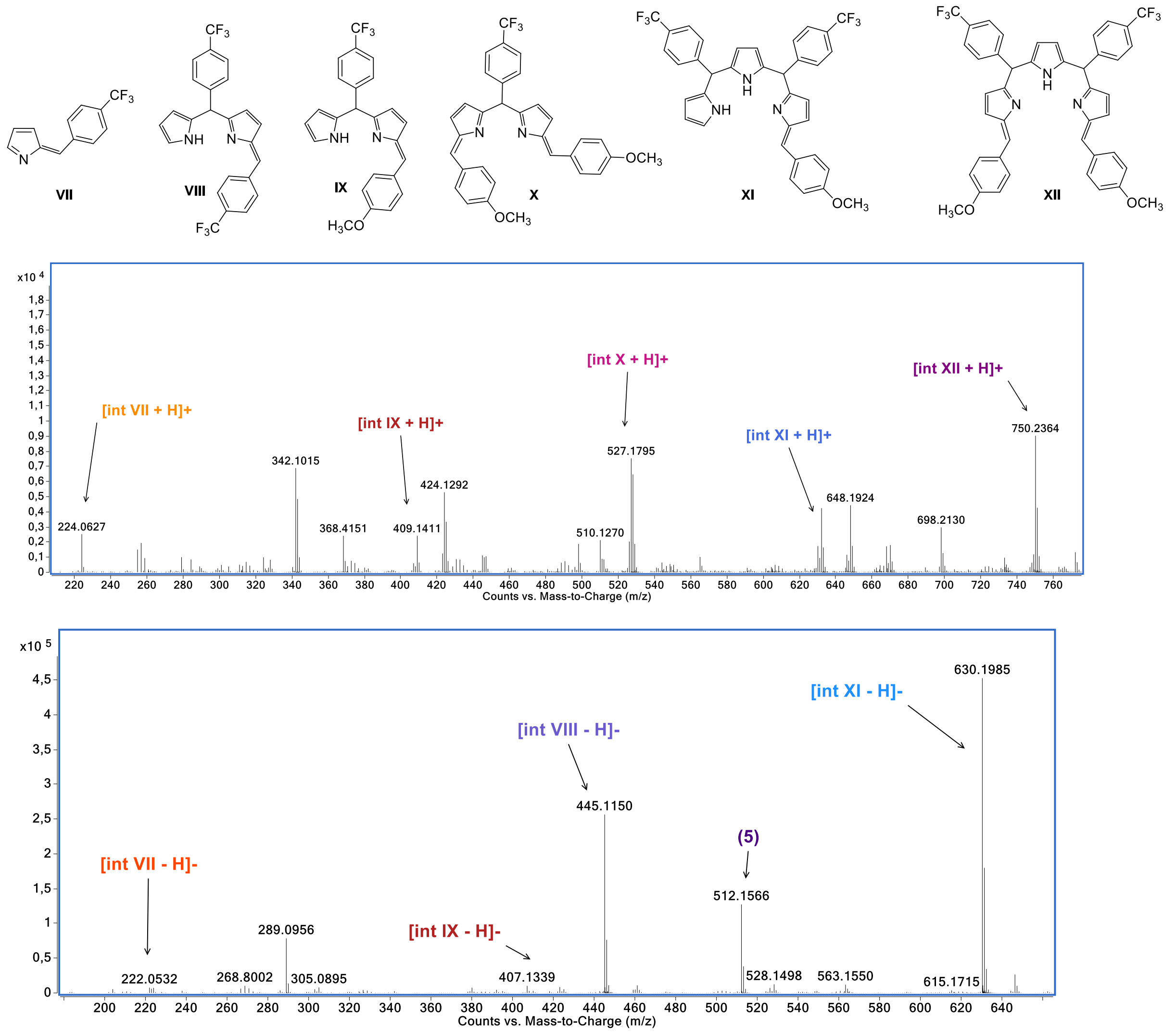

Figure S49. Intermediates **VII** - **XII** in **5** and **2h** reaction mixture at 0 °C for 30 min.

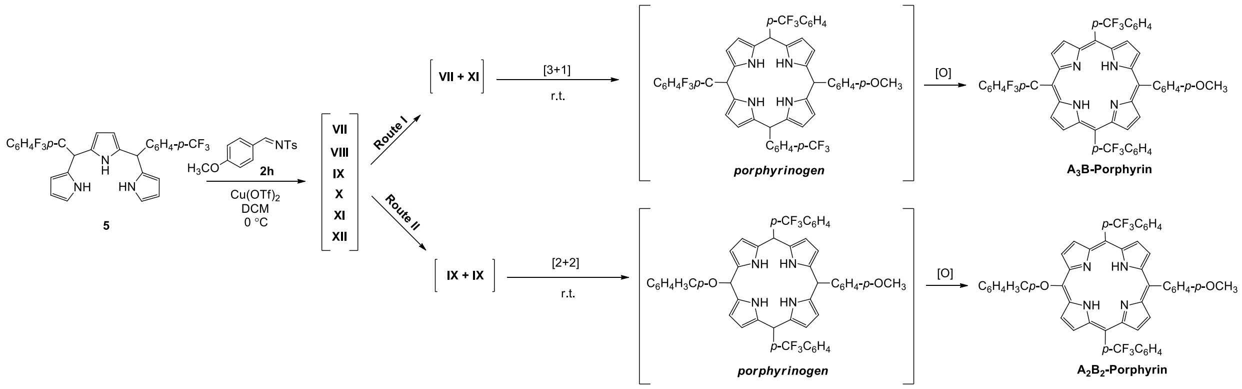

Figure S50. A suggested reaction pathway for the formation of A_3B-porphyrins and A_2B_2-porphyrins

6. LC MS-TOF (ESI) spectra of compounds

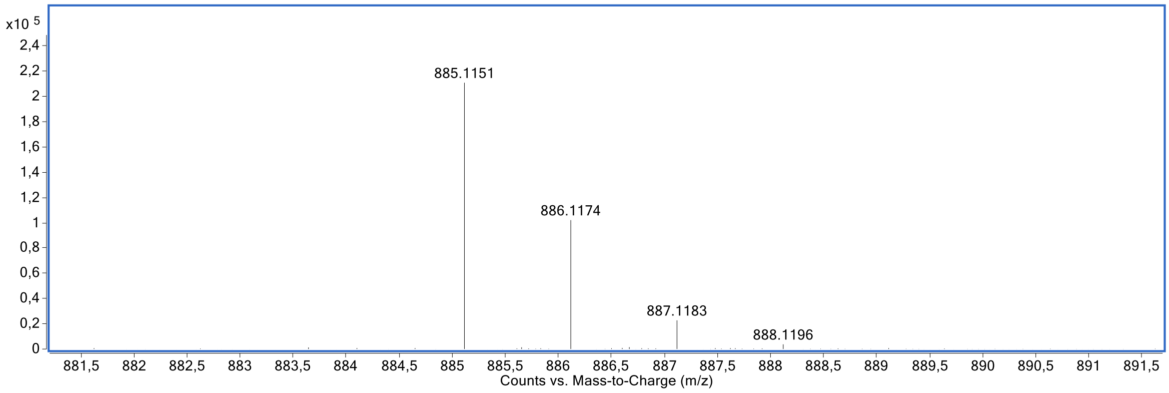

Figure S51. ESI-TOF mass spectrum of **3a**.

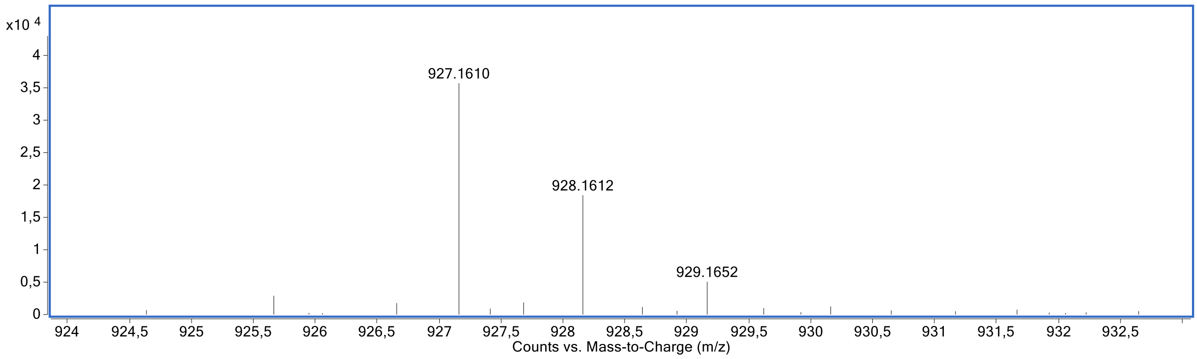

Figure S52. ESI-TOF mass spectrum of **3b**.

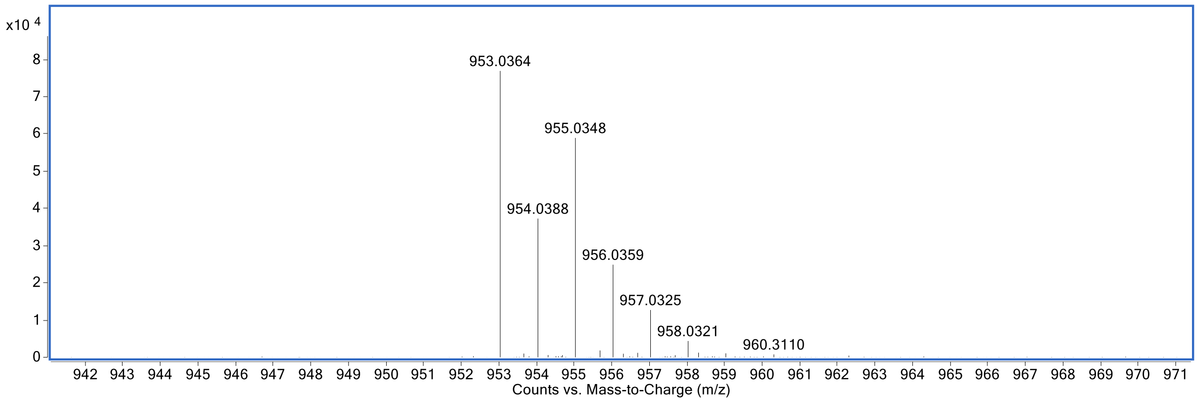

Figure S53. ESI-TOF mass spectrum of **3c**.

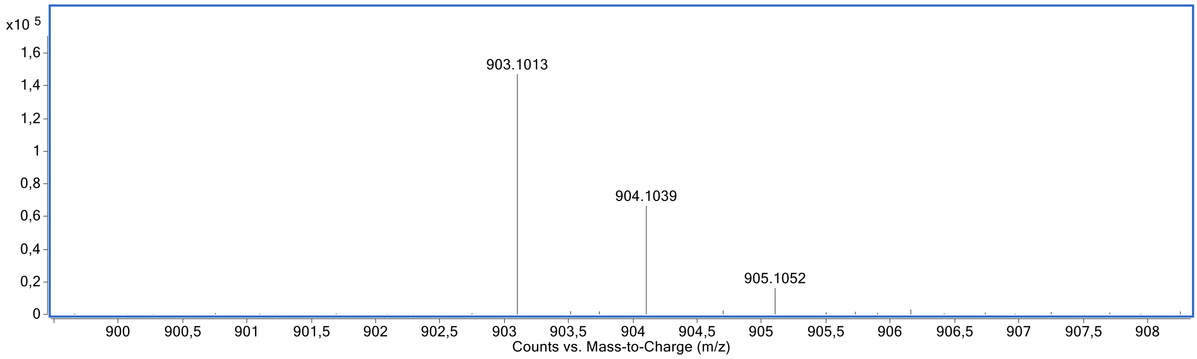

Figure S54. ESI-TOF mass spectrum of **3d**.

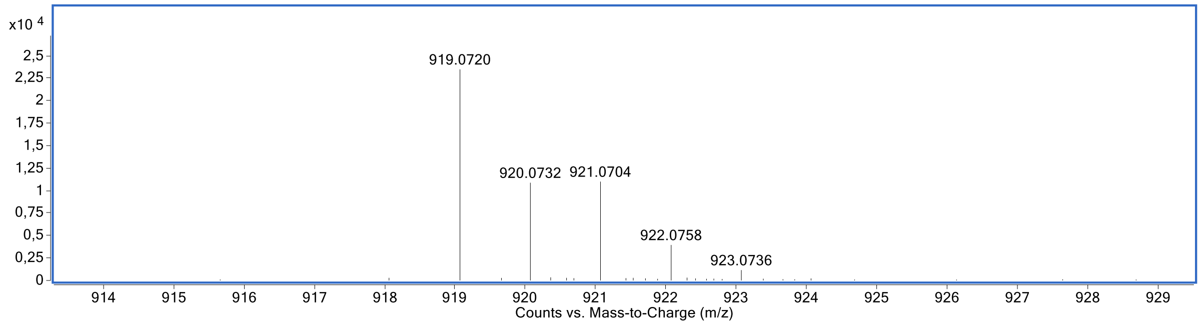

Figure S55. ESI-TOF mass spectrum of **3e**.

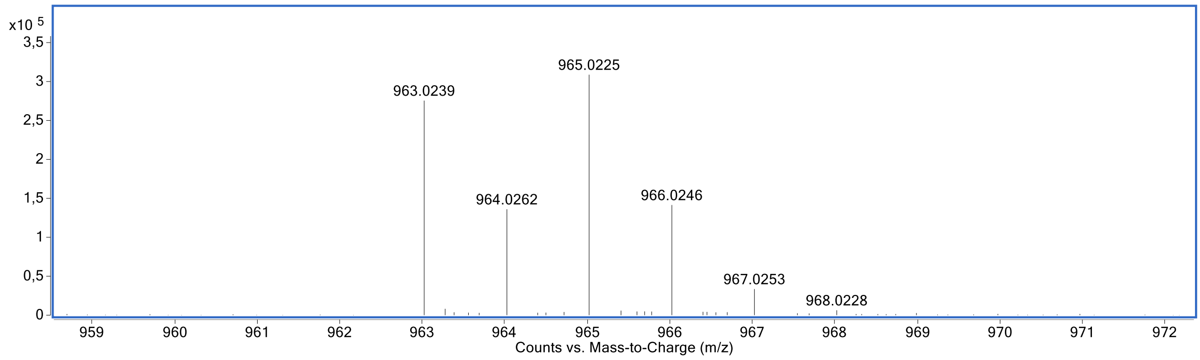

Figure S56. ESI-TOF mass spectrum of **3f**.

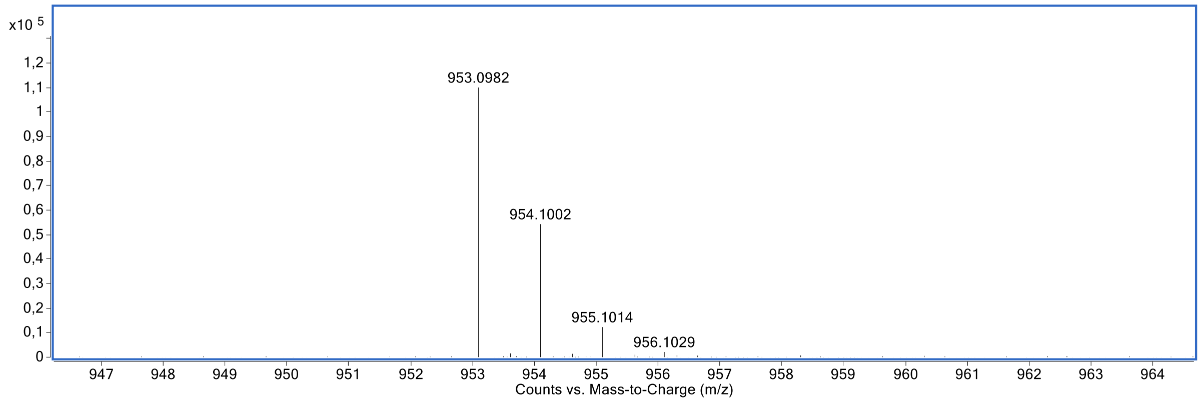

Figure S57. ESI-TOF mass spectrum of **3g**.

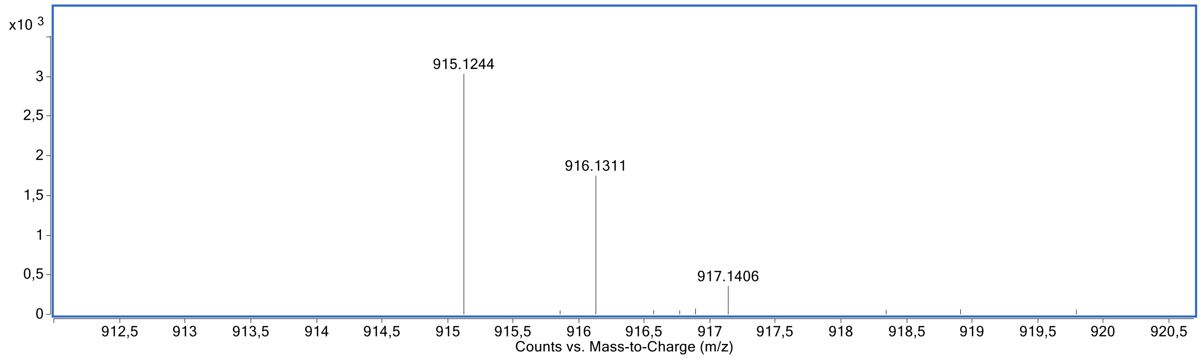

Figure S58. ESI-TOF mass spectrum of **3h**.

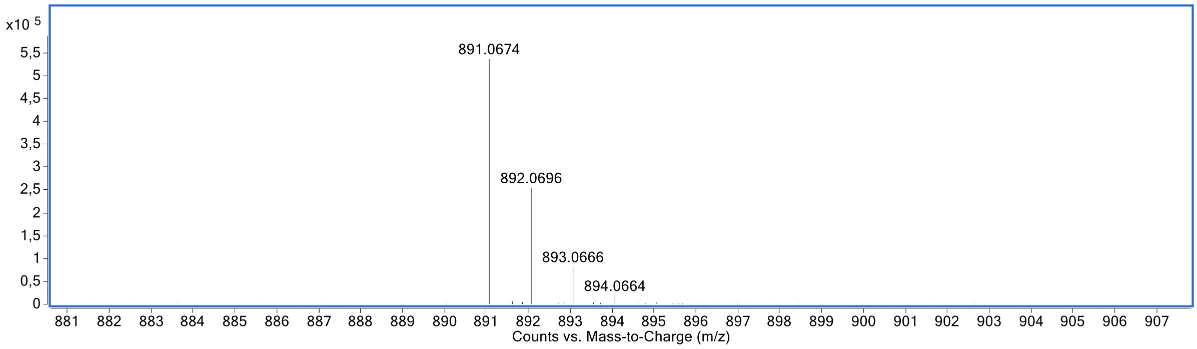

Figure S59. ESI-TOF mass spectrum of **3j**.

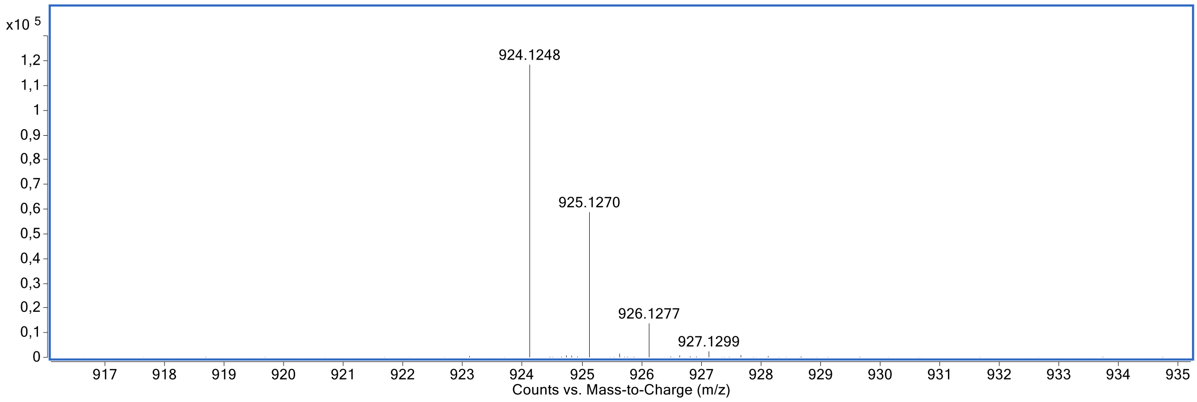

Figure S60. ESI-TOF mass spectrum of **3k**.

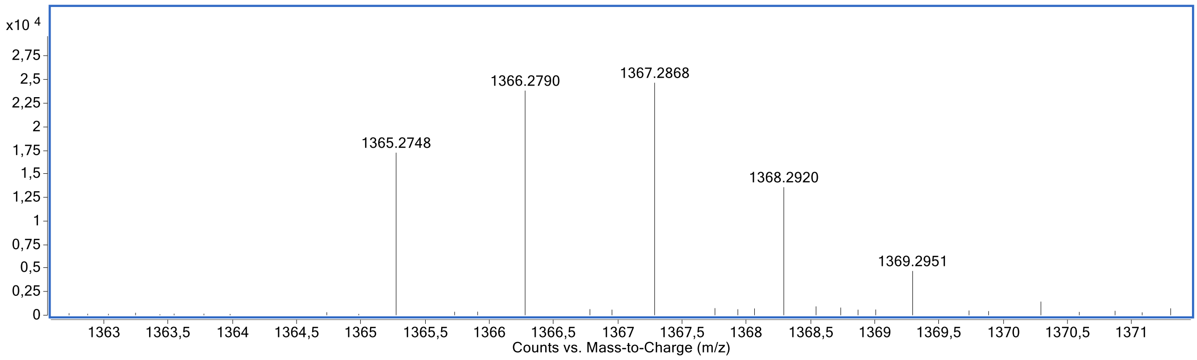

Figure S61. ESI-TOF mass spectrum of **4b**.

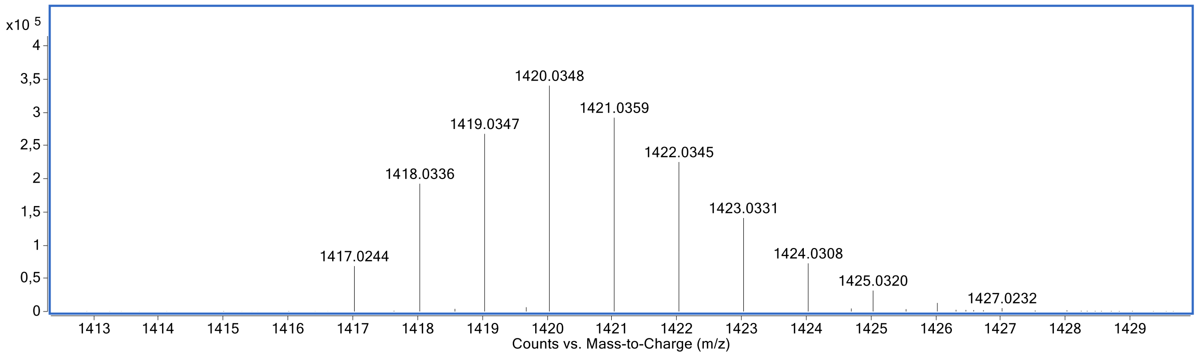

Figure S62. ESI-TOF mass spectrum of **4c**.

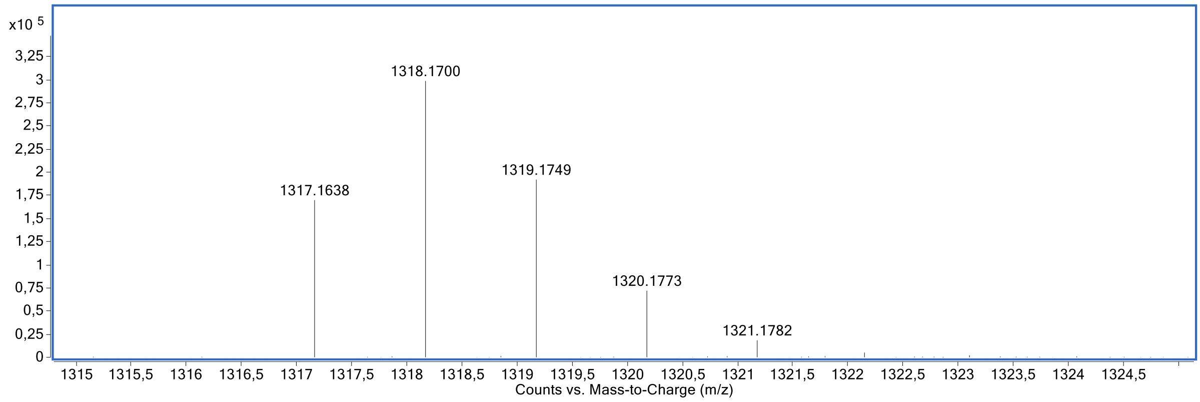

Figure S63. ESI-TOF mass spectrum of **4d**.

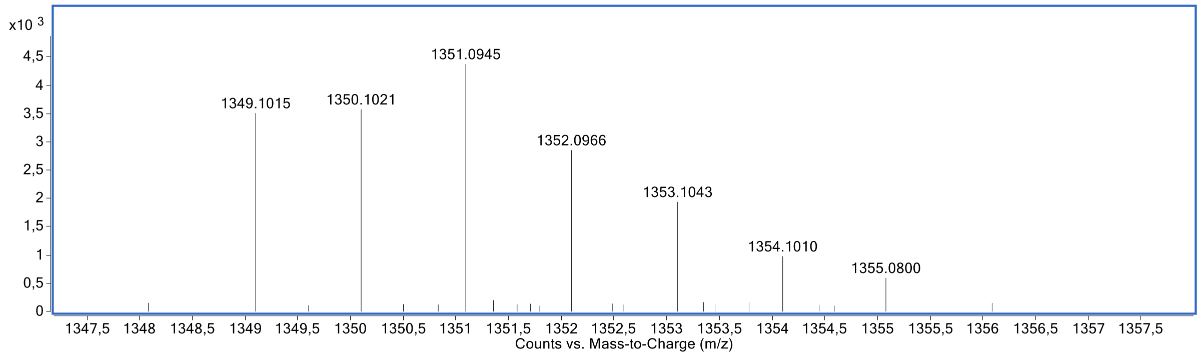

Figure S64. ESI-TOF mass spectrum of **4e**.

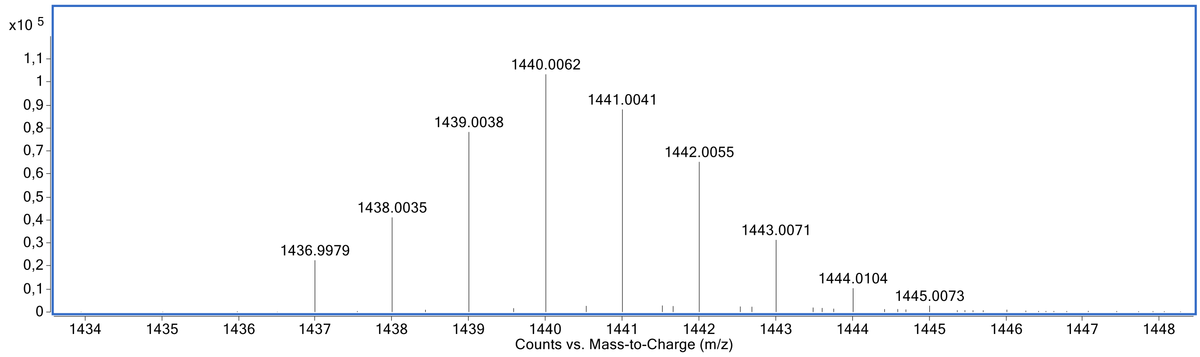

Figure S65. ESI-TOF mass spectrum of **4f**.

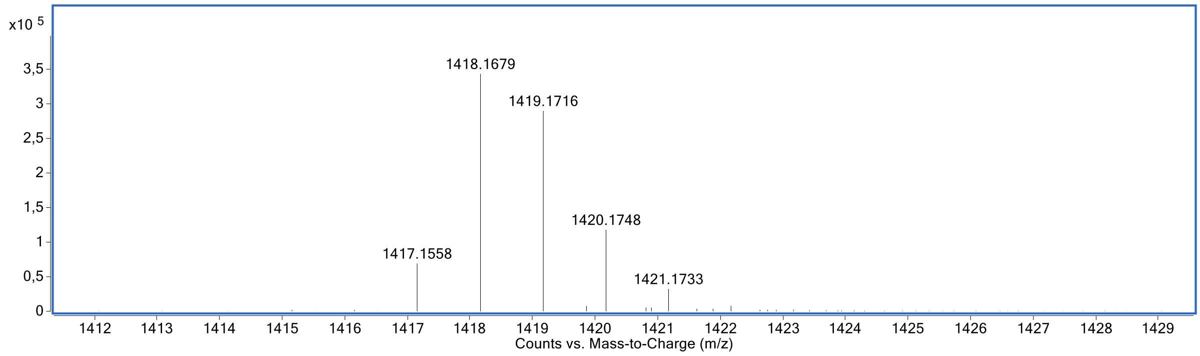

Figure S66. ESI-TOF mass spectrum of **4g**.

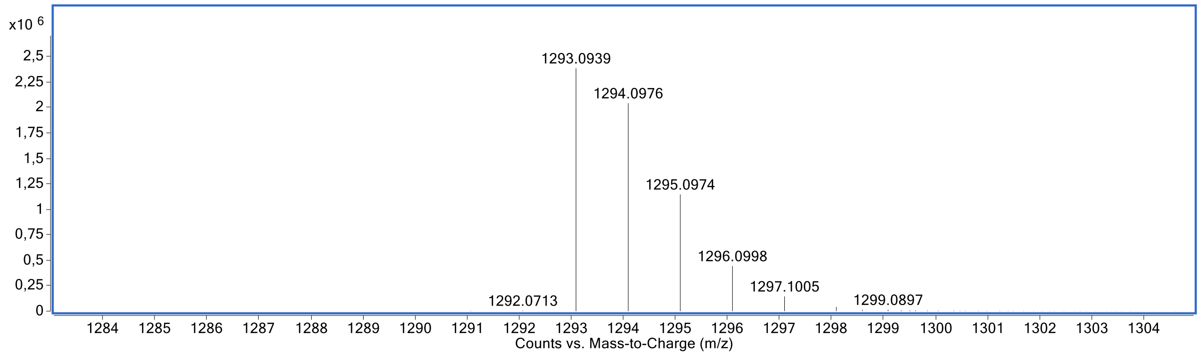

Figure S67. ESI-TOF mass spectrum of **4j**.

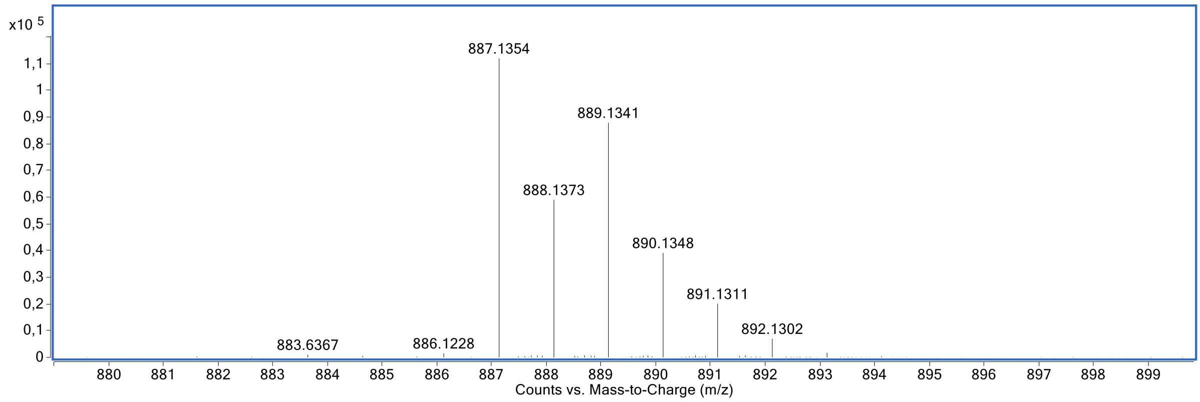

Figure S68. ESI-TOF mass spectrum of **6a**.

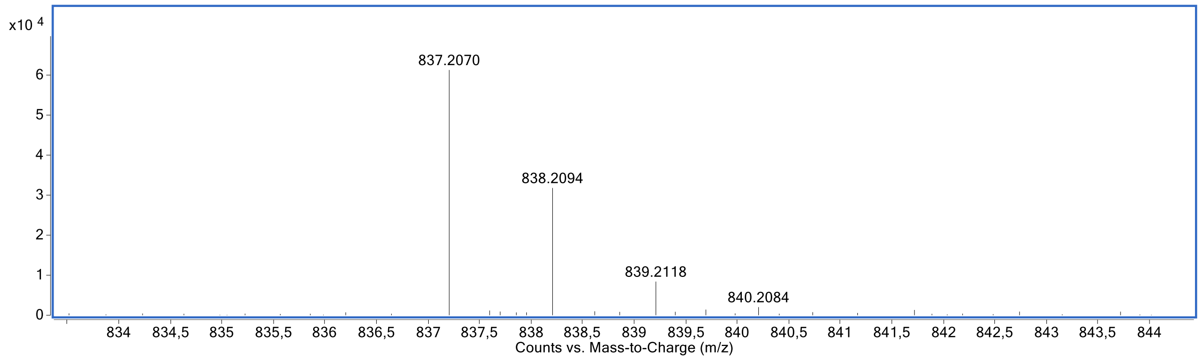

Figure S69. ESI-TOF mass spectrum of **6b**.

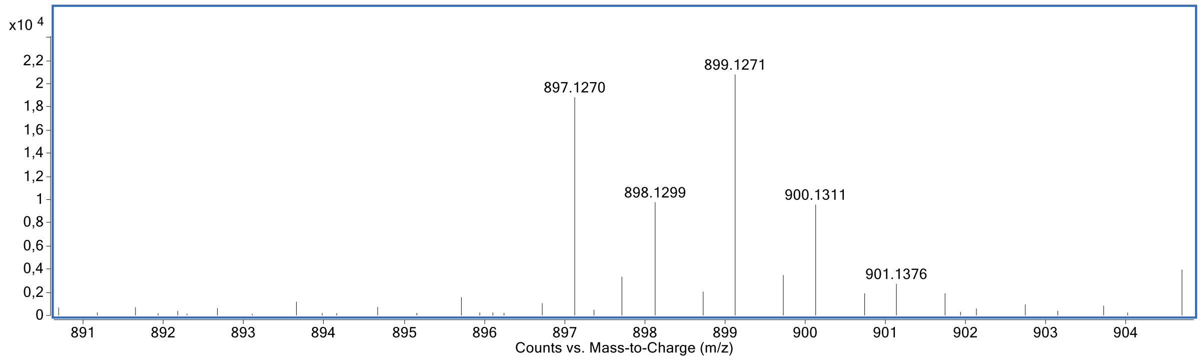

Figure S70. ESI-TOF mass spectrum of **6c**.

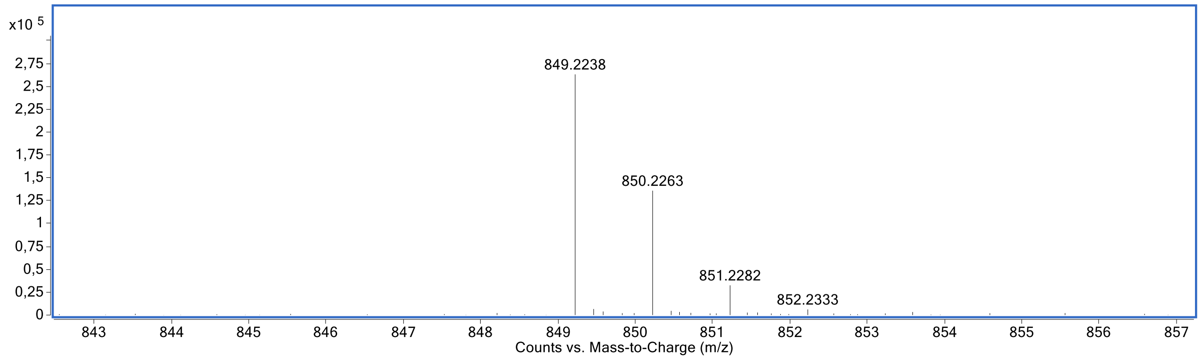

Figure S71. ESI-TOF mass spectrum of **6d**.

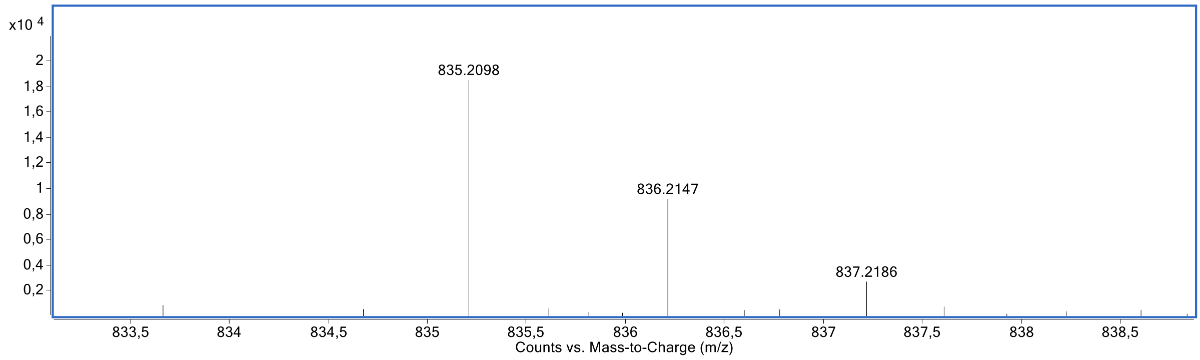

Figure S72. ESI-TOF mass spectrum of **6e**.

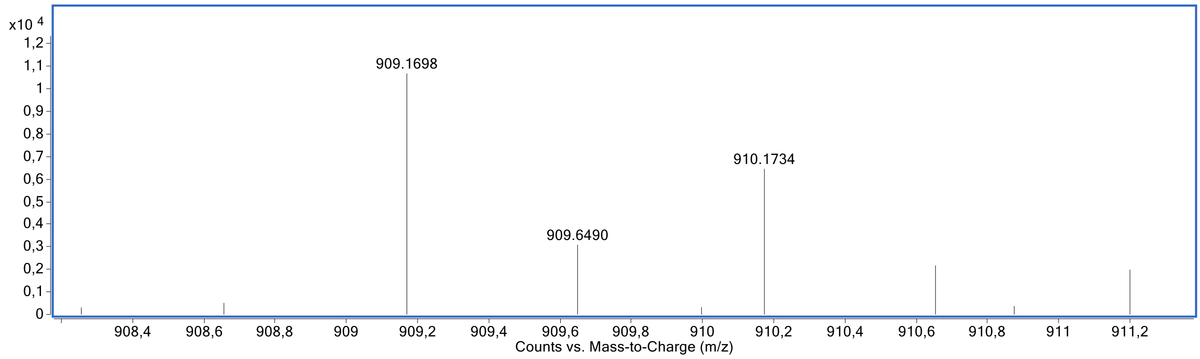

Figure S73. ESI-TOF mass spectrum of **6f**.

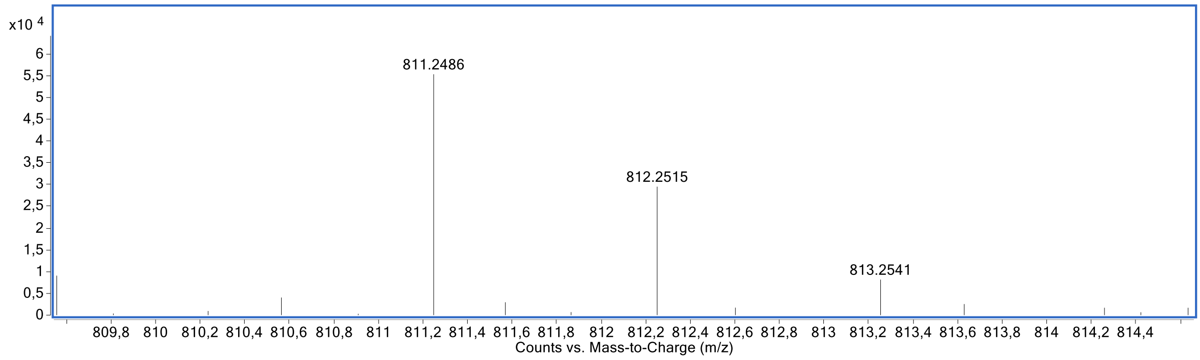

Figure S74. ESI-TOF mass spectrum of **7d**.

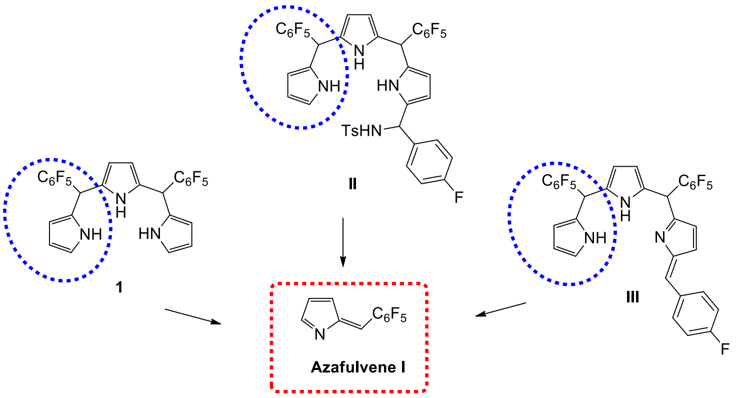

Figure S75. Possible fragmentation for the formation of azafulvene

References

[1] M. Suzuki, A. Osuka, *Chem. Eur. J.* **2007**, *13*, 196 – 202.

[2] G. R. Fulmer, A. J. M. Miller, N. H. Sherdan, H. E. Gottlieb, A. Nudelman, B. M. Stoltz, J. E. Bercaw, K. I. Goldberg, *J. Org. Chem.* **1997**, *62*, 7512 – 7515.
